# Characterizing cellular heterogeneity in fibrotic hypersensitivity pneumonitis by single-cell transcriptional analysis

**DOI:** 10.1038/s41420-022-00831-x

**Published:** 2022-01-28

**Authors:** Junyi Wang, Lei Zhang, Li Luo, Ping He, Anying Xiong, Manling Jiang, Yao Liu, Shengbin Liu, Qin Ran, Dehong Wu, Ying Xiong, Xiang He, Guoping Li

**Affiliations:** 1 Laboratory of Allergy and Precision Medicine, Chengdu Institute of Respiratory Health, the Third People’s Hospital of Chengdu, Affiliated Hospital of Southwest Jiaotong University, Chengdu, China; 2 Department of Pulmonary and Critical Care Medicine, Chengdu Third People’s Hospital Branch of National Clinical Research Center for Respiratory Disease, Affiliated Hospital of ChongQing Medical University, Chengdu, China; 3 State Key Laboratory of Quality Research in Chinese Medicine, Macau University of Science & Technology, Taipa, Macau SAR, China; 4 Department of Pulmonary and Critical Care Medicine, Sichuan Friendship Hospital, Chengdu, China

**Keywords:** Acute inflammation, Allergy

## Abstract

Fibrotic hypersensitivity pneumonitis (FHP) remains one of fatal interstitial pulmonary disease. Comprehensively dissecting the cellular heterogeneity of FHP paves the way for developing general gene therapeutic solutions for FHP. Here, utilizing an integrated strategy based on scRNA-seq, scTCR-seq, and bulk RNA-seq analysis of FHP profiles, we identified ten major cell types and 19 unique subtypes. FHP exhibited higher features of EMT and inflammation-promoting than normal control. In distinct subsets of lung macrophages in FHP, FN1^high^, PLA2G7^high^, and MS4A6A^high^ macrophages with predominant M2 phenotype exhibited higher activity of inflammatory responses and para-inflammation than other macrophages. KRT17^high^ basal-like epithelial cells were significantly increased in FHP, and showed higher ability to induce EMT. We identified roles for ACTA2^high^, COL1A1^high^, and PLA2G2A^high^ fibroblasts in FHP, which were significantly related to interstitial fibrosis. NK cells and KLRG1^+^ effector CD8^+^ T cells had greater activity in inflammation-promoting. Our results provide a comprehensive portrait of cellular heterogeneity in FHP, and highlight the indispensable role of cell subpopulations in shaping the complexity and heterogeneity of FHP. These subpopulations are potentially key players for FHP pathogenesis.

## Introduction

Hypersensitivity pneumonitis (HP) is a complex syndrome with pulmonary alveolar unit inflammation or interstitial fibrosis caused by the inhalation of a variety of antigens in susceptible and sensitized individuals [1]. HP was divided into two categories including fibrotic and non-fibrotic HP in Official ATS/JRS/ALAT Clinical Practice Guideline [2]. Compared with the incidence of idiopathic pulmonary fibrosis (IPF) which is approximately 4.6–16.3 per 100,000 person-years, the incidence of HP remains under-recognized [3]. Despite recent advances in diagnosis, classification, and therapeutic management of HP, FHP remains difficult to diagnose because of non-specific clinical syndrome and high-resolution computed tomography (HRCT) features. The exposure of antigens has not been clearly identified in many HP cases [3, 4]. Bronchoalveolar lavage fluid (BALF) lymphocytosis and low CD4/CD8 ratio are also not consistently in fibrotic HP [3, 5, 6].

FHP is a lethal interstitial lung disease, and yet it remains understudied and poorly understood compared with IPF. The pathological features of chronic hypersensitivity pneumonitis include alternating normal alveoli and fibroblastic foci, a nonspecific interstitial pneumonia-like pattern, and centrilobular fibrosis [7]. Specific and sensitive biochemical markers for diagnosis, treatment guidance, and prognosis monitoring of FHP are urgently needed [8]. Genome-guided therapies are still unavailable for worsening Fibrotic HP patients, who suffer extremely poor clinical prognosis. Undoubtedly, an urgent need exists to characterize core molecular features of fibrotic HP for the discovery of emerging biomarkers and therapeutic targets. The latest advances in single-cell RNA sequencing technologies offer an opportunity to comprehensively understand the regulatory network and cellular heterogeneity of FHP at high resolution. These technologies have already identified disease-associated cell subsets in rheumatoid arthritis pathogenesis [9].

In this present study, through the integration of the expression profiling of surgical lung biopsy specimens from a patient with FHP and published study databases including both bulk RNA-seq and scRNA-seq in Gene Expression Omnibus (GEO), we identified the contributions of specific cell subsets relevant to FHP. We also uncovered the dynamic changes of specific cell subsets and identified a number of novel functional candidates in FHP by performing integrated analysis of expression profiling of 82 chronic hypersensitivity pneumonitis (CHP) lung samples from published studies.

## Results

### Single-cell transcriptional profiling of lung cells

**Fig. 1:**
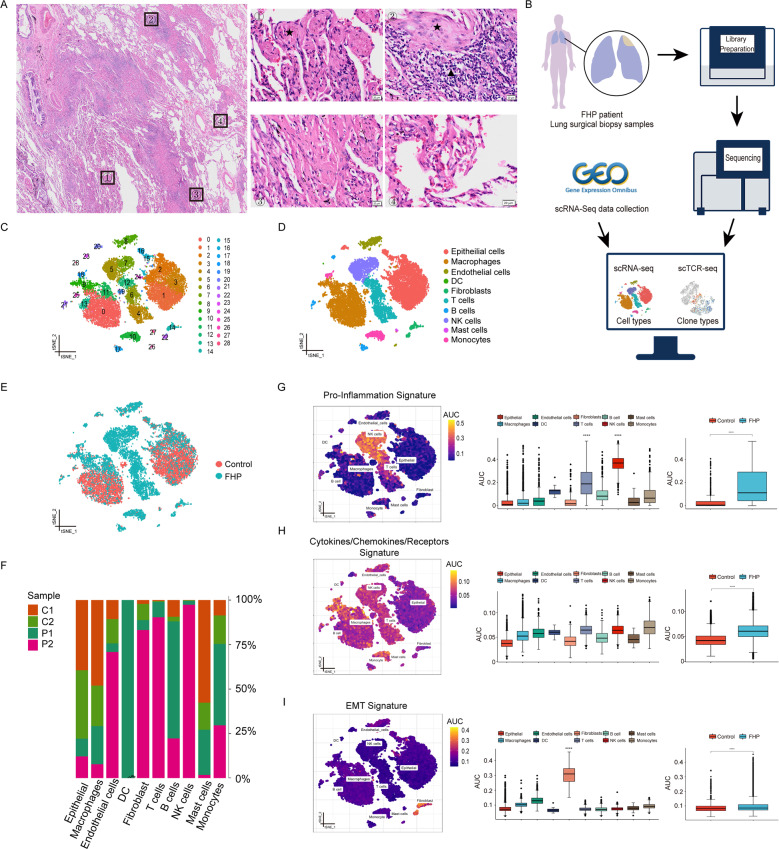
Study design and single-cell transcriptional profiling of lung cells. **A** H&E stanning of biopsy specimens in the left upper lobe from the patient with FHP showing massive fibroblast foci (black star) and mono-nuclear cell infiltration (black triangle). Scale bar = 20 μm. **B** Schematic of the overall study design. The scRNA-seq and scTCR-seq were applied to surgical lung biopsy specimens of the FHP patient, and the output data were integrated with single-cell transcriptome data from an FHP patient and two transplant donors in a publicly available dataset for analysis. **C** t-SNE visualization of 29 cell clusters. **D** t-SNE visualization of epithelial cells, macrophages, endothelial cells, dendritic cells, fibroblasts, T cells, B cells, NK cells, mast cells, and monocytes. DC dendritic cells. **E** The t-SNE map showing the distribution of cells between control and FHP group. **F** Relative percentage of sample origins across cell types. **G**–**I** AUCell analyses of the relative gene set enrichment scores in Pro-inflammation signature (**G**), Cytokines/Chemokines/Receptors signature (**H**), and EMT (**I**) signature. The t-SNE maps showing AUC scores of selected gene signatures (left); the box plots showing AUC scores of selected gene signatures in each cell type (middle) or sample group (right). *P < 0.05, **P < 0.01, ***P < 0.001, ****P < 0.0001.

We collected fresh specimens from a patient with pathologically confirmed FHP through surgical lung biopsy (Fig. 1A and Supplementary Fig. 1). scRNA-seq and scTCR-seq was performed using 10× Genomics platform. We integrated multiple data from GEO data repository including an FHP patient (GSM3489192) (P1) and two transplant donors (GSM3489182, GSM3489185) (C1, C2) with the sequencing data of the patient at our hospital (P2) (Fig. 1B). A total of 17,755 lung cells were analyzed, with 8761 cells from donors and 8994 cells from patients with FHP. Twenty-nine cell clusters were identified with graph-based clustering analysis (Fig. 1C). Ten major cell types were manually annotated based on canonical marker genes (Fig. 1D). We identified epithelial cells (expressing SFTPA1), macrophages (expressing C1QB, C1QA, and C1QC), endothelial cells (expressing VWF and CLDN5), dendritic cells (DC) (expressing LILRA4 and GZMB), fibroblasts (expressing COL1A1 and COL1A2), T cells (expressing CD3E and IL7R), B cells (expressing IGHG3 and IGLC2), natural killer (NK) cells (expressing GNLY and NKG7), mast cells (expressing MS4A2), and monocytes (expressing S100A8 and FCN1) (Supplementary Fig. 2A). Gene Ontology (GO) and Kyoto Encyclopedia of Genes and Genomes (KEGG) enrichment analyses revealed features of these cells (Supplementary Fig. 2B, C), which were consistent with previous single-cell analysis of interstitial lung diseases [10, 11].

Compared with donors, patients with FHP exhibited expanded populations of fibroblasts, T cells and NK cells (Fig. 1E, F and Supplementary Fig. 3A, B). Enrichment analyses of inflammatory response and immune related signatures using AUCell (Fig. 1G–I and Supplementary Fig. 4A–D) found the greater enrichment of Inflammatory Response signature in macrophages and monocytes. Fibroblasts displayed greater enrichment of epithelial mesenchymal transition (EMT) signature. Macrophages and fibroblasts had significantly higher enrichment of Cytokine signatures. NK cells and T cells showed greater enrichment of Inflammation-promoting signatures. Also, we found that FHP had higher enrichment in Inflammation-promoting, EMT, and Cytokines/Chemokines/Receptors signatures than normal control. Taken together, FHP exhibited higher enrichment of inflammatory response, inflammation-promoting, and EMT signatures than normal control.

### Single-cell RNA-seq analysis reveals disease-specific macrophage subpopulations in FHP lungs

**Fig. 2:**
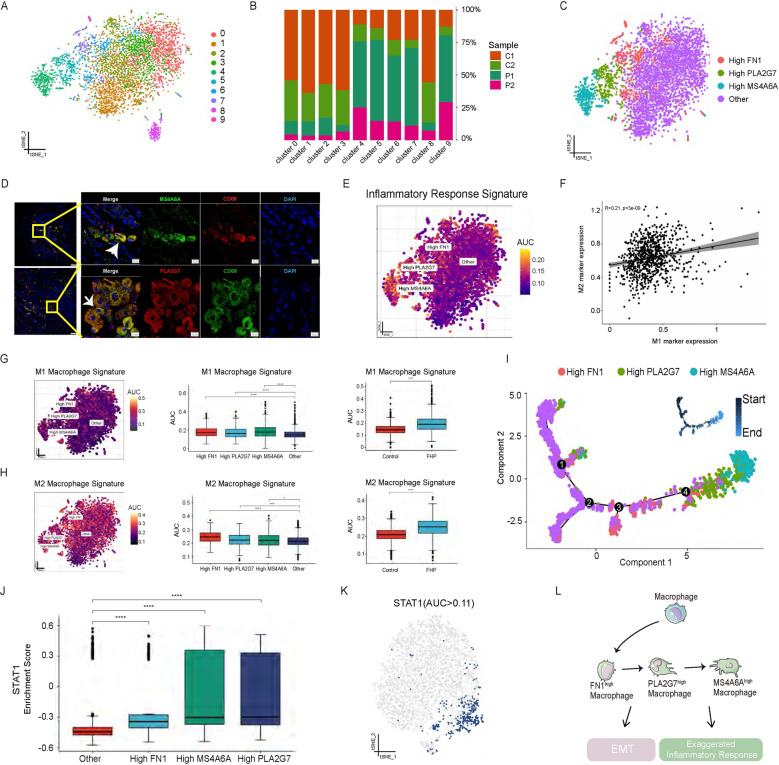
Single-cell RNA-seq analysis reveals disease-specific macrophage subpopulations in FHP lungs. **A** t-SNE visualization of ten macrophage subclusters. **B** Relative percentage of sample origins across macrophage subclusters. **C** t-SNE visualization of FN1^high^ macrophages, PLA2G7^high^ macrophages, MS4A6A^high^ macrophages, and other macrophages. **D** Multiplex immunofluorescence images of CD68, PLA2G7, and MS4A6A in lung tissues from the patient with FHP. Scale bar = 10 μm. White arrow, CD68^+^ MS4A6A^+^ cells(upper)/CD68^+^ PLA2G7^+^ cells(lower). **E** AUCell analysis of the relative gene set enrichment scores in Inflammatory response signature. **F** Scatter plot showing the correlation between M1 and M2 marker gene expression. **G**, **H** AUCell analyses of the relative gene set enrichment scores in M1 (**G**) and M2 (**H**) macrophage signatures. The t-SNE maps showing AUC scores of selected gene signatures (left); the box plots showing AUC scores of selected gene signatures in each cell type (middle) or sample group (right). **I** Trajectory analysis of FN1^high^ macrophages, PLA2G7^high^ macrophages, MS4A6A^high^ macrophages and other macrophages using Monocle 2. **J** STAT1 motif showed greater enrichment of regulon activity for PLA2G7^high^ and MS4A6A^high^ macrophages using SCENIC analysis. **K** t-SNE visualization of AUC values of STAT1 motif in macrophages. **L** Schematic developmental trajectories of macrophage subpopulations in FHP lungs. *P < 0.05, **P < 0.01, ***P < 0.001, ****P < 0.0001.

Macrophages have a crucial role in the development of ILD and pulmonary fibrosis [12]. To identify the macrophage subpopulations overabundant in FHP, macrophages were divided into ten subclusters (cluster 0-9) (Fig. 2A and Supplementary Fig. 5A). We further analyzed the difference of these subclusters in FHP and normal lungs. We found that cluster 4, 5, 6, 7, and 9 were prominent macrophage subclusters in FHP subjects (Fig. 2B and Supplementary Fig. 5B). In contrast, cluster 0, 1, 2, and 3 were predominant macrophage subsets in the normal lungs. Next, we identified three predominant macrophage subtypes in FHP: PLA2G7^high^ (expressing PLA2G7), FN1^high^ (expressing FN1), and MS4A6A^high^ macrophages (expressing MS4A6A) (Fig. 2C and Supplementary Fig. 5C, D). GO enrichment analysis indicated that FN1^high^ macrophages were enriched in neutrophil activation. PLA2G7^high^ and MS4A6A^high^ macrophages were involved in neutrophil activation, myeloid leukocyte migration and leukocyte chemo taxis (Supplementary Fig. 6A).

Using AUCell analysis, we found an increase of inflammatory response, para-inflammation, and EMT signatures in FHP compared to those in normal control (Supplementary Fig. 6B–D). Moreover, we identified 140 upregulated and 73 downregulated genes through differential gene-expression (DEG) analysis of macrophages between FHP and normal control (Supplementary Fig. 7A). The expression levels of CCL4, CCL2, CCL18, PLA2G7, SPP1 MS4A6A, FN1, and STAT1 in FHP macrophages were higher than those in macrophages from normal control (Supplementary Fig. 7B). Cytokines and chemokines expression analyses showed that macrophages from FHP displayed higher expression levels of CCL4, CCL18, CXCL10, CCL5, CCL3, and TNF than those from normal control (Supplementary Fig. 7C). GO enrichment analysis indicated that upregulated DEGs of FHP-specific macrophages were predominantly enriched in leukocyte chemotaxis, leukocyte migration, and myeloid leukocyte migration (Supplementary Fig. 7D). To explore the potential biological function of PLA2G7 in FHP, we defined the PLA2G7^high^ macrophages based on PLA2G7 expression levels greater than 1 (Supplementary Fig. 8A). PLA2G7^high^ macrophages exhibited higher expression levels of chemokines (CCL18, CXCL10, and CCL2), IL1RN, cysteine cathepsins (CTSB), lipid transport-related genes (APOE) and TYMP compared with PLA2G7^low^ macrophages (Supplementary Fig. 8B). PLA2G7^high^ macrophages also showed stronger activities of leukocyte migration and cell chemotaxis (Supplementary Fig. 8C).

Next, we performed multiplex immunofluorescence stain on tissue with CD68, PLA2G7, and MS4A6A antibodies. The results revealed that the infiltrations of PLA2G7^high^ and MS4A6A^high^ macrophages existed in lungs with FHP (Fig. 2D). In addition, we also found that FHP-specific macrophage subsets highly expressed CCL4 (Supplementary Fig. 9A–C), implicating the importance of chemotactic function in the development of fibrotic hypersensitivity pneumonitis. We next found the greater enrichment of Inflammatory Response signature in PLA2G7^high^ and MS4A6A^high^ macrophages (Fig. 2E).

Macrophages were defined as classically-activated M1 and alternatively-activated M2 phenotypes depending on surface protein markers [10]. We assessed the enrichment of M1 and M2 signature gene sets using AUCell. FN1^high^, PLA2G7^high^, and MS4A6A^high^ macrophages were found to show significantly higher enrichment of M2 and M1 gene signatures than other macrophages (Fig. 2G, H). FHP showed significantly higher enrichment of M2 and M1 gene signatures than normal control. There was a positive correlation between M1 and M2 markers (Fig. 2F). Those results indicated that FHP-specific macrophages displayed as a mixed state of M1 and M2 phenotypes. We next sought to investigate the developmental trajectories of macrophage subpopulations using Monocle 2 during FHP [13]. PLA2G7^high^ and MS4A6A^high^ macrophages were found at later developmental time points in FHP (Fig. 2I and Supplementary Fig. 10A–C). FN1^high^ macrophages were connected to early time points by a “bridge” of macrophages defined as other cells. In addition, PPARG_extended motifs displayed higher gene regulatory activity for other and FN1^high^ macrophages by using Single-Cell Regulatory Network Inference and Clustering (SCENIC) [14] (Supplementary Fig. 11A). STAT1 motif was highly activated in PLA2G7^high^, MS4A6A^high^, and FN1^high^ macrophages (Fig. 2J, K and Supplementary Fig. 11B, C). Taken together, our findings revealed that FHP samples were characterized by high abundance of FN1^high^, PLA2G7^high^, and MS4A6A^high^ macrophage subpopulations. Specifically, these types of cells mainly drove inflammatory responses and EMT in FHP (Fig. 2L).

### Single-cell transcriptional analysis reveals heterogeneity of alveolar epithelial cells in FHP patients

**Fig. 3:**
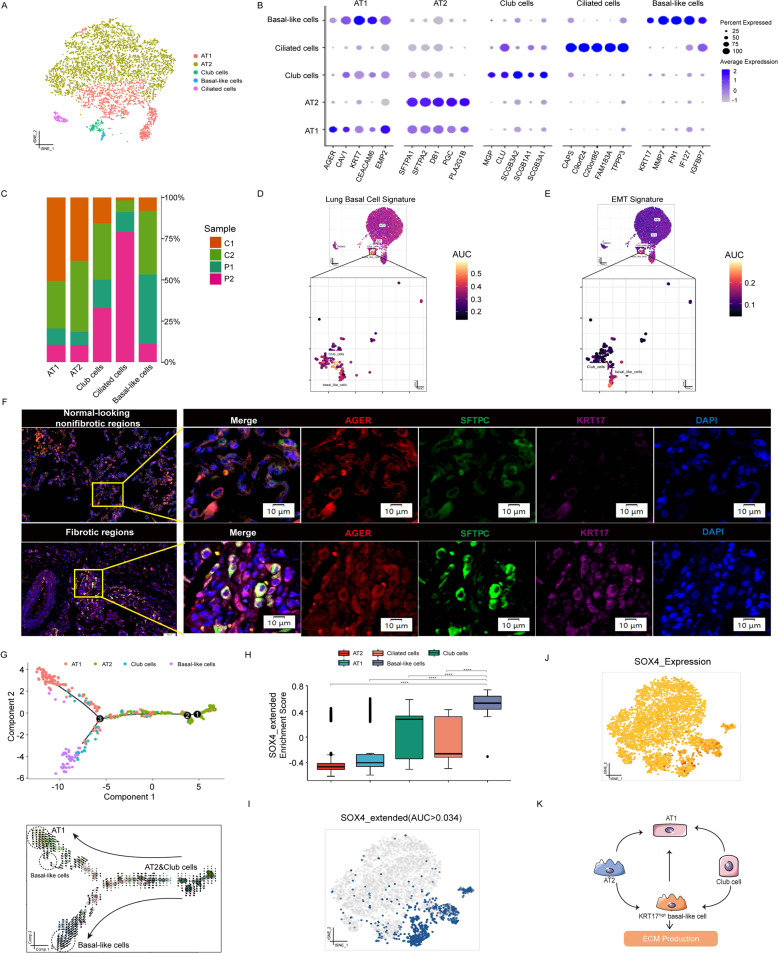
Single-cell transcriptional analysis reveals heterogeneity of alveolar epithelial cells in FHP patients. **A** t-SNE visualization of AT1 cells, AT2 cells, club cells, ciliated cells, and KRT17^high^ basal-like cells. AT1: alveolar type I cells. AT2: alveolar type II cells. **B** The expression levels of top five marker genes in cell subtypes. **C** Relative percentage of sample origins across cell subtypes. **D**, **E** AUCell analyses of the relative gene set enrichment scores in Lung Basal Cell signature (**D**) and EMT signature (**E**). **F** Multiplex immunofluorescence images of AGER, SFTPC, and KRT17 in lung samples from the patient with FHP. Scale bar = 10 μm. **G** Trajectory analysis of AT1 cells, AT2 cells, club cells, and KRT17^high^ basal-like cells using Monocle 2 (upper) and Velocyto R package (lower). **H** SOX4_extended motif showed greater enrichment of regulon activity for KRT17^high^ basal-like cells using SCENIC analysis. **I** t-SNE visualization of AUC values of SOX4_extended motif in alveolar epithelial cells. **J** t-SNE visualization of expression levels of SOX4 in alveolar epithelial cells. **K** Schematic developmental trajectories of KRT17^high^ basal-like cell in FHP lungs. *P < 0.05, **P < 0.01, ***P < 0.001, ****P < 0.0001.

Alveolar regeneration plays an important role in human lung fibrosis [15, 16]. We identified 13 subclusters (cluster 11 likely represented cell doublets), which were manually annotated based on canonical marker genes, typically assessing the top five differentially expressed genes across clusters (Fig. 3A, B and Supplementary Fig. 12A, B). We identified alveolar type 1 (AT1) cells (expressing AGER and KRT7), alveolar type 2 (AT2) cells (expressing SFTPA1 and SFTPA2), club cells (expressing SCGB3A2 and SCGB1A1), and ciliated cells (expressing TPPP3 and CAPS). We also defined a KRT17^high^ basal-like cell subpopulation with high expression of KRT17 (Fig. 3A, B). FHP patients exhibited increased proportions of KRT17^high^ basal-like cells (Fig. 3C and Supplementary Fig. 12C). Besides, we found that KRT17^high^ basal-like cells exhibited higher expression levels of COL1A1, FN1, COL6A2, and MMP7 (Supplementary Fig. 12D). Patients with FHP showed higher expression levels of VEGFA, KRT8, KRT18, KRT7, and MMP7 than normal control.

We next assessed the enrichment of gene sets with cell type signatures and hallmark gene signatures (http://www.gsea-msigdb.org/) using AUCell. We found the greater activity of lung basal cell and EMT gene sets in KRT17^high^ basal-like cells (Fig. 3D, E and Supplementary Fig. 13A, B). To explore the potential biological function of KRT17 in FHP, we here defined KRT17^high^ cell subpopulation based on KRT17 expression greater than 1 (Supplementary Fig. 14A). KRT17^high^ cells showed stronger activities of ECM organization, extracellular structure organization, cell-substrate adhesion, and regulation of actin filament-based process than KRT17^low^ cells (Supplementary Fig. 14B, C). Enrichment in processes regarding epithelial layer development was also observed in KRT17^high^ cells. The presence of KRT17^high^ basal-like epithelial cells were further validated in fibrotic areas of FHP lung by multiplex immunofluorescence staining (Fig. 3F).

We next sought to investigate the developmental trajectories of KRT17^high^ basal-like cells using Monocle 2 and Velocyto R package. KRT17^high^ basal-like cells and AT1 cells were found at later developmental time points in FHP. AT2 cells and club cells were connected to early time points. RNA velocity analysis demonstrated that KRT17^high^ basal-like cells and AT1 cells have higher transcriptional activity than other cells (Fig. 3G and Supplementary Fig. 15). Using SCENIC and GSVA analysis, we also found that SOX4_extended motif had a key role in transcriptional regulation of KRT17^high^ basal-like cells (Fig. 3H–J and Supplementary Fig. 16A, B). Together, our results indicated that KRT17^high^ basal-like epithelial cells were a crucial factor in determining the prognosis of EMT in FHP (Fig. 3K).

### Single-cell transcriptional analysis unravels heterogeneity of fibroblasts during FHP

**Fig. 4:**
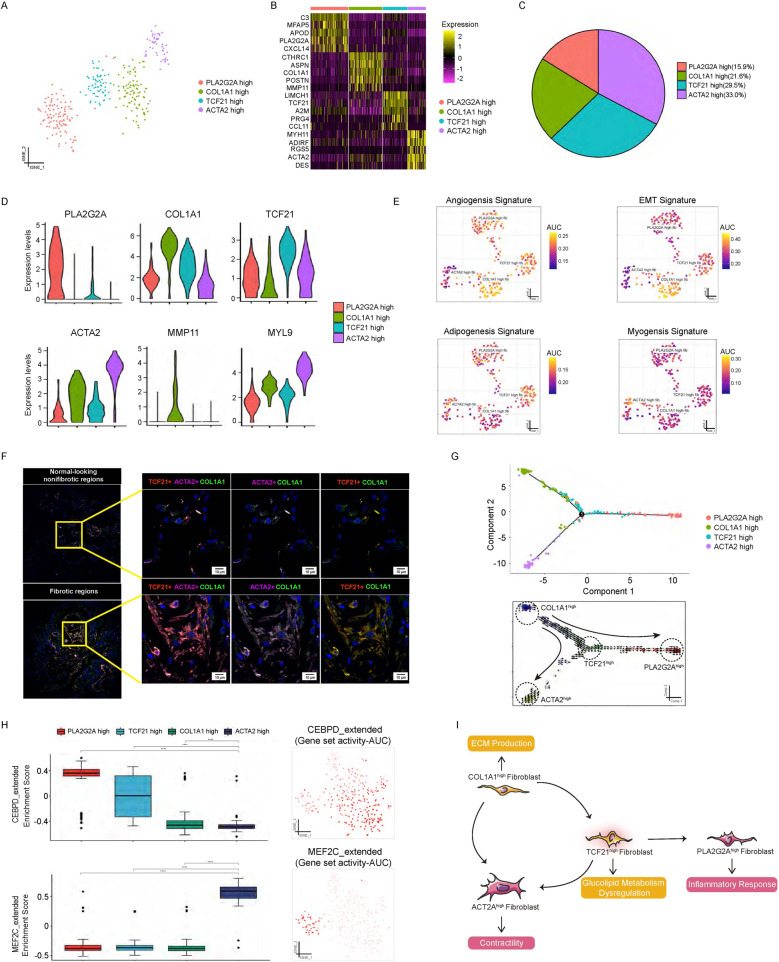
Single-Cell transcriptional analysis unravels heterogeneity of fibroblasts during FHP. **A** t-SNE visualization of PLA2G2A^high^ fibroblasts, COL1A1^high^ fibroblasts, TCF21^high^ fibroblasts, and ACTA2^high^ fibroblasts. **B** The heatmap showing the expression levels of top five marker genes in fibroblast subpopulations. **C** Relative percentage of fibroblast subpopulations in lung from the patient with FHP. **D** Violin plots displaying the expression levels of the representative genes in each fibroblast subpopulation. **E** Relative gene set enrichment scores of subpopulations calculated by AUCell analysis. **F** Multiplex immunofluorescence images of TCF21, ACTA2, and COL1A1 in lung tissues from FHP. Scale bar = 10 μm. **G** Trajectory analysis of PLA2G2A^high^ fibroblasts, COL1A1^high^ fibroblasts, TCF21^high^ fibroblasts, and ACTA2^high^ fibroblasts using Monocle 2 (upper) and Velocyto R package (lower). **H** CEBPD_extended motif showed greater enrichment of regulon activity for PLA2G2A^high^ fibroblasts using SCENIC analysis (left panel). MEF2C_extended motif showed greater enrichment of regulon activity for ACTA2^high^ fibroblasts (left panel). t-SNE visualization of AUC values of CEBPD_extended and MEF2C_extended motifs in fibroblasts (right panel). **I** Schematic developmental trajectories of fibroblast subpopulations in FHP lungs. *P < 0.05, **P < 0.01, ***P < 0.001, ****P < 0.0001.

Pulmonary fibrosis is characterized by lung fibroblasts differentiation to myofibroblasts and excessive extracellular matrix accumulation [17]. Here, we identified four subclusters, which were manually annotated based on typically assessing the top five differentially expressed genes across clusters (Fig. 4A–D and Supplementary Fig. 17A, B). Cluster 0 was identified as PLA2G2A^high^ fibroblasts (expressing PLA2G2A, APOD, and C3). Cluster 1 was identified as COL1A1^high^ fibroblast based on high expression of COL1A1 genes. Cluster 2 was identified as interstitial fibroblasts (TCF21^high^ fibroblasts) based on high expression of TCF21. Cluster 3 was identified as myofibroblasts (ACTA2^high^ fibroblasts) with higher levels of ACTA2 and MYH11.

We next identified the active gene sets in cell subtypes using AUCell. We found the greater activity of myogenesis in hallmark gene sets in ACTA2^high^ fibroblasts. COL1A1^high^ fibroblast showed greater activity of EMT. TCF21^high^ fibroblasts had significantly higher activity of adipogenesis and glycolysis than other cell types. PLA2G2A^high^ fibroblasts exhibited greater activity of angiogenesis and Inflammatory Response signatures (Fig. 4E and Supplementary Fig. 17C). The presence of ACTA2^high^ and TCF21^high^ fibroblasts were further validated in fibrotic areas of FHP lung by multiplex immunofluorescence staining (Fig. 4F).

We next sought to investigate the developmental trajectories of fibroblast subtypes using Monocle 2 and Velocyto R package. ACTA2^high^ fibroblasts and PLA2G2A^high^ fibroblasts were found at later developmental time points in FHP. COL1A1^high^ fibroblasts were connected to early time points. TCF21^high^ fibroblasts were showed at middle developmental time points in FHP (Fig. 4G and Supplementary Fig. 18A, B). Using SCENIC and GSVA analysis, we also found that NR2F2_extended, MEF2C and MEF2C_extended were specific motifs that had key roles in transcriptional regulation of ACTA2^high^ fibroblasts (Fig. 4H and Supplementary Fig. 19). CEBPD and CEBPD-extended motifs were highly activated in PLA2G2A^high^ fibroblasts. Together, our results indicated that FHP patients showed high cell abundances of ACTA2^high^ fibroblasts, COL1A1^high^ fibroblasts, TCF21^high^ fibroblasts, and PLA2G2A^high^ fibroblasts (Fig. 4I).

### Single-Cell transcriptional analysis unveils functional heterogeneity of T cells and NK cells in patients with FHP

**Fig. 5:**
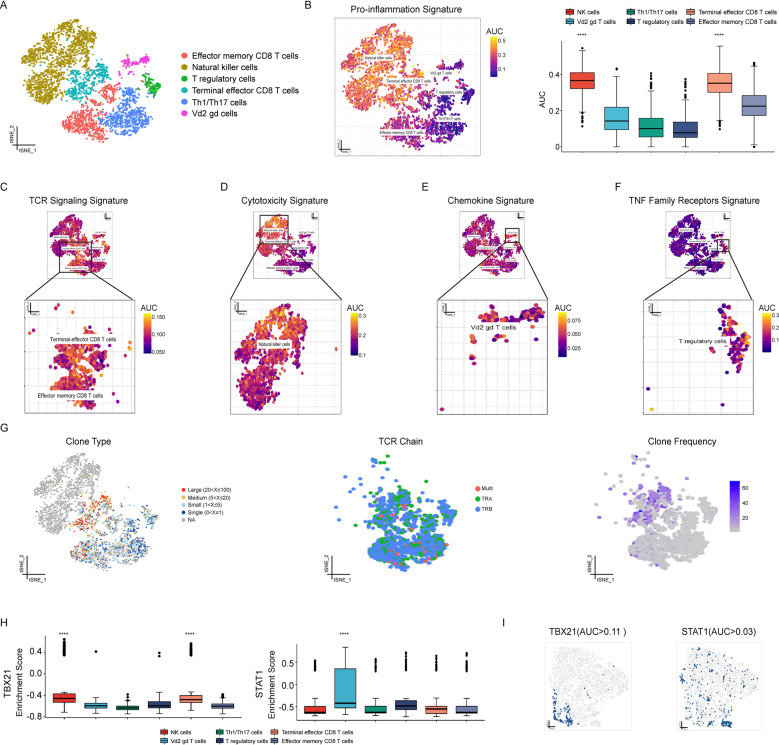
Single-cell transcriptional analysis unveils functional heterogeneity of T cells and NK cells in patients with FHP. **A** t-SNE visualization of effector memory CD8^+^ T cells, NK cells, T regulatory cells, KLRG1^+^ CD8^+^ terminal effector T cells, Th1/Th17 cells, and Vd2 gamma delta T cells. **B** AUCell analysis of the relative gene set enrichment scores in Pro-inflammation signature. The t-SNE map showing AUC scores of selected gene signature (left); the box plot showing AUC scores of selected gene signature in each cell type (right). **C**–**F** AUCell analyses of the relative gene set enrichment scores in TCR Signaling signature (**C**), Cytotoxicity signature (**D**), Chemokine signature (**E**), and TNF Family Receptors signature (**F**). **G** t-SNE visualization of TCR clone type. Cells are colored based on clone size (left), TCR chains (middle), or clone frequency (right). **H** TBX21 motif showed greater enrichment of regulon activity for NK cells and terminal effector CD8^+^ T cells using SCENIC analysis (left); STAT1 motif showed greater enrichment of regulon activity for Vd2 gamma delta T cells using SCENIC analysis (right). **I** t-SNE visualization of AUC values of TBX21 (left) or STAT1 (right) in T cells and NK cells. *P < 0.05, **P < 0.01, ***P < 0.001, ****P < 0.0001.

Patients with HP have significantly higher numbers of NK cells and CD8^+^ cells in BALF [18]. T cells and NK cells were integrated and reclustered to allow clearer identification of subpopulations for further analysis. We identified six major cell subtypes based on SingleR in combination with manual adjustment with canonical markers and top three differentially expressed genes across clusters (Fig. 5A and Supplementary Fig. 20A–C). Effector memory CD8^+^ T cells (CD8^+^ Tem) (expressing CD8B and GZMK), NK cells with (expressing FCGR3A), T regulatory cells (Tregs) (expressing RGS1 and TNFRSF4), KLRG1^+^ terminal effector CD8^+^ T cells (KLRG1^+^ effector CD8^+^ T cells) (expressing CD8A and KLRG1), Th1/Th17 cells (expressing IL7R), and Vd2 gamma delta T cells (γδ T cell) (expressing IGKC and GNB2L1) were identified.

AUCell analysis with inflammation-promoting genes showed greater enrichment of Pro-inflammatory signatures in NK cells and KLRG1^+^ effector CD8^+^ T cells compared to all other T cells (Fig. 5B). Tregs, Th1/Th17 cells and γδ T cells displayed lower Pro-inflammatory signatures. Next, we assessed immune related gene signatures using AUCell (Fig. 5C–F and Supplementary Fig. 20D–H). We found NK cells with higher activity of Cytotoxicity signature, γδ T cells with greater activity of Chemokine Receptor signature, Tregs with greater enrichment of TNF Family Member Receptors signature, CD8^+^ Tem and KLRG1^+^ CD8^+^ effector T cells with greater enrichment of TCR Signaling Pathway signatures. We also observed that KLRG1^+^ effector CD8^+^ T cells showed high proportions of clonal cells and high degrees of clonal expansion by using scTCR-seq (Fig. 5G). Using SCENIC and GSVA analysis, we also found that TBX21 motif had a key role in transcriptional regulation of NK cells and KLRG1+ effector CD8+ T cells (Fig. 5H, I and Supplementary Fig. 20I). STAT1 motif was highly activated in γδ T cells. Overall, these results demonstrated that different subsets of T cells and NK cells played varying roles in inflammatory responses during FHP.

### Integrated analysis in scRNA-seq and bulk RNA-seq data

**Fig. 6:**
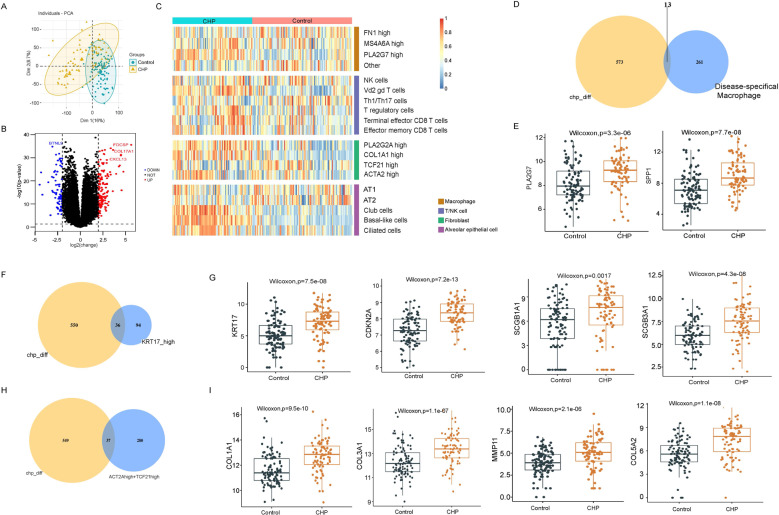
Integrated analysis in scRNA-seq and bulk RNA-seq data. **A** 2-dimensional plot of PCA analysis. **B** Volcano plot showing DEGs between CHP lungs and control lungs. **C** Heatmap for ssGSEA score across two sample groups according to top 20 marker genes of cell subtypes based on scRNA-seq analysis. **D**, **F**, **H** Venn diagrams showing overlapping genes between DEGs in CHP lungs and DEGs in specific cell subtypes. **E**, **G**, **I** The box plots displaying expression levels of selected genes between CHP and control lungs.

To better assess these subpopulations identified in our studies, we used ssGSEA to quantify the level of subpopulations in CHP samples from GSE150910. The gene signatures were based on top 20 marker genes for each cell subpopulation in scRNA-seq analysis. After normalizing for the total number of cell subpopulation per sample, CHP showed higher enrichment of PLA2G7^high^ macrophage, γδ T cell, CD8^+^ T cell, PLA2G2A^high^ fibroblast, COL1A1^high^ fibroblast, ACTA2^high^ fibroblast, and KRT17^high^ basal-like cell signatures compared with health control (Fig. 6A–C).

Next, we examined the expression levels of common shared DEGs on both bulk RNA-seq data and scRNA-seq data. We found that the highly expressed genes, PLA2G7 and SPP1, in FHP-specific macrophage subpopulations were significantly increased in bulk RNA-seq of CHP lungs (Fig. 6D, E). Moreover, after intersecting with the 130 DEGs in KRT17^high^ basal-like epithelial cells, 36 genes were found in both bulk RNA-seq data and scRNA-seq data (Fig. 6F). Among them, expression levels of ECM-producing genes (COL1A1), secretory cell markers (SCGB1A1), senescence-associated genes (CDKN2A), as well as KRT17 were higher in CHP lungs compared to those of healthy lungs (Fig. 6G and Supplementary Fig. 21A). Afterward, we combined ACTA2^high^ and TCF21^high^ fibroblasts to obtain a total of 317 DEGs. The same method was used to obtain 37 overlapping genes, such as COL1A1, COL3A1, MMP11, and so on (Fig. 6H, I and Supplementary Fig. 21B). These results indicated that FHP was in an active EMT and inflammatory response with greater abundance of PLA2G7^high^ macrophages, γδ T cell, CD8^+^ T cell, PLA2G2A^high^ fibroblast, COL1A1^high^ fibroblast, ACTA2^high^ fibroblast, and KRT17^high^ basal-like epithelial cells in both bulk RNA-seq and scRNA-seq data.

### Single-cell transcriptional analysis reveals the cell-cell crosstalk network in FHP

**Fig. 7:**
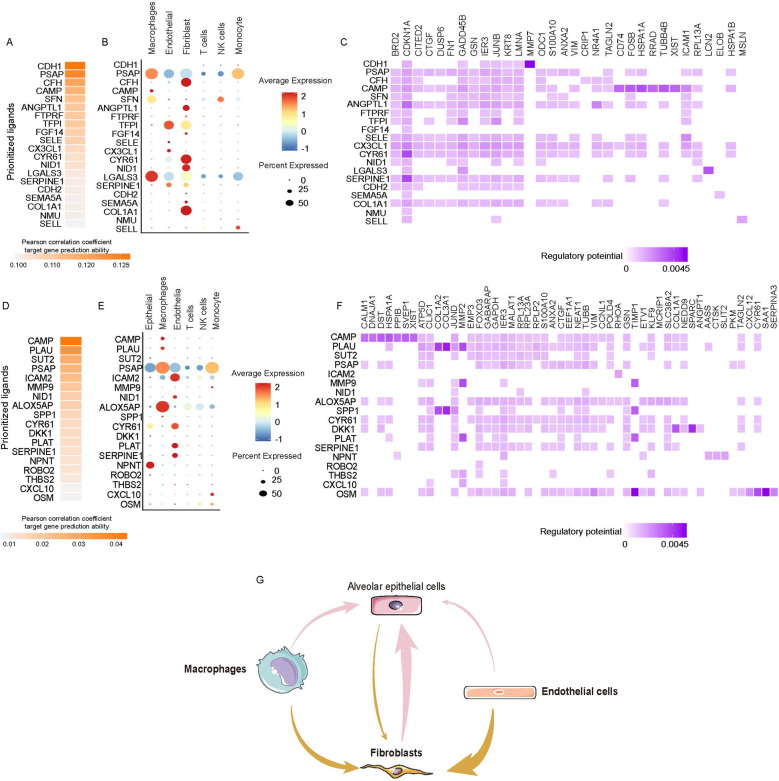
Single-cell transcriptional analysis reveals the cell-cell crosstalk network in FHP. **A** Ligand activity prediction by NicheNet showing the top 20 ligands best predicting the entire DEGs in epithelial cells between FHP and control lungs. **B** The bubble plot showing expression patterns of the predicted ligands on macrophages, endothelial cells, fibroblasts, T cells, NK cells and monocytes. **C** Ligand–target matrix displaying the regulatory potential of predicted ligands on target genes from the epithelial cells in FHP lungs. **D** The top 20 ligands best predicting the entire DEGs in fibroblasts between FHP and normal lungs. **E** The bubble plot showing expression patterns of the predicted ligands on epithelial cells, macrophages, endothelial cells, T cells, NK cells, and monocytes. **C** Ligand–target matrix displaying the regulatory potential of predicted ligands on target genes from the fibroblasts in FHP lungs. **G** Schematic showing the inferred cell-cell interaction network during FHP.

We predicted ligand-target links between interacting cells by combining their expression data using NicheNet R package (https://github.com/saeyslab/nichenetr). Epithelial cells and fibroblasts are the primary effector cells during pulmonary fibrosis including IPF and FHP [11, 19–21]. We designated epithelial cells or fibroblasts as ‘receiver’ to elucidate cell–cell regulatory networks [22]. Figure 7A showed top 20 ligands probable regulating epithelial cells. The heatmap showed the regulatory potential between ligands and target genes expressed in epithelial cells (Fig. 7C). Interesting, we found the increased expression levels of COL1A1, NID1, CYR61, ANGPTL1, and CFH in fibroblasts had the regulatory potential for KRT8 which highly expressed in basal-like cells (Fig. 7B, C). We also found higher expression levels of CAMP, PLAU, PSAP, ALOX5AP, and SPP1 in macrophages showed the regulatory potential for fibroblasts (Fig. 7D–F). Increased SPP1 and PLAU in macrophages showed higher regulatory potential for COL1A2 and COL3A1. Increased MMP9 in monocyte, increased PLAU deriving from macrophages, and increased PLAT deriving from endothelia showed the regulatory potential for MMP2 and MMP3. Overall, these data inferred a sophisticated and extensive cell–cell interaction network during FHP (Fig. 7G).

## Discussion

Integrating bulk RNA-seq and scRNA-seq data to study the characteristics of diseases has gradually become more popular and common. Through this method, researchers can obtain more reliable and meaningful results in cellular heterogeneity. In this study, we identified ten major cell types and 19 subtypes at the single cell level. We found that EMT and inflammatory responses were important characteristic in progressive fibrosis in FHP. In distinct subsets of lung in FHP, macrophages and monocytes showed the greater activity of inflammatory responses. Fibroblasts exhibited greater activity of EMT. Macrophages and fibroblasts had significantly higher activity of cytokine signatures. We observed that fibroblasts had the greater regulatory potential for KRT17^high^ basal-like epithelial cells with increased expression levels of COL1A1, NID1, CYR61, ANGPTL1, and CFH. We also observed that macrophages, monocytes and endothelia cells had the great regulatory potential for fibroblasts through SPP1, PLAU, MMP9, PLAU, and PLAT.

Lung macrophages have distinct M1 and M2 cell subtypes in pulmonary fibrosis diseases. Enhanced macrophage M2 program is associated with fibrotic remodeling of lungs [23]. MS4A6A and FN1 have been linked to the M2 macrophage phenotype [24, 25]. Pro-inflammatory macrophages in lungs emerging with progression of COVID-19 show predominantly increased PLA2G7 expression [26]. Alveolar macrophages from ILD patients express higher levels of CHI3L1, MARCKS, IL1RN, PLA2G7, MMP9, and SPP1 [10]. SPP1^high^ macrophages exist in normal lungs while tend to increase dramatically in fibrotic lungs [27]. We here evaluated the role of lung macrophages in FHP through AUCell. Patients with FHP had a prominent high score of inflammatory responses and EMT. Meanwhile, we found that PLA2G7^high^, FN1^high^, and MS4A6A^high^ macrophages were closely related to inflammatory responses and para-inflammation. Moreover, we also elucidated the developmental trajectories of PLA2G7^high^ and MS4A6A^high^ macrophages in FHP. STAT1 had higher transcription factor activity in PLA2G7^high^ and MS4A6A^high^ macrophages. The results demonstrated that the inflammatory responses and EMT in FHP may relate to PLA2G7^high^ and MS4A6A^high^ macrophages, and STAT1 is able to regulate polarizations of these cell type.

Terminal differentiation from KRT8^+^ transitional cells to alveolar type-1 cells can cause aberrant persistence of regenerative intermediate stem cell states in human lung fibrosis [16]. The loss of Cdc42 function in alqaveolar stem cells (AT2 cells) leads to progressive lung fibrosis [28]. We here identified the cell subtypes of alveolar epithelial cells including AT1, AT2, club cells, ciliated cells, and KRT17^high^ basal-like cells. We found KRT17^high^ basal-like cells showed greater AUC value of EMT with high expression levels of KRT17, COL1A1, FN1, COL6A2, VEGFA, KRT8, KRT18, KRT7, and MMP7. These genes were highly expressed in FHP lungs. Previous study has revealed the differentiation trajectories from AT2 to KRT5^-^/KRT17^+^ cell state in human IPF [29]. We found that basal-like cells retain a higher transcriptional activity of KRT17 gene including splicing and un-splicing RNA. Moreover, we illustrated the differentiation trajectories of AT2 and club cells into KRT17^high^ basal-like cells. In addition, we also found that SOX4 regulates KRT17^high^ basal-like cells in FHP.

Studies of IPF have suggested pathological functions of abnormal fibroblasts. Fibrotic lungs contain a myofibroblast phenotype enriched with collagens and ACTA2. Some fibroblast phenotypes that exhibit increased expression of chemokines [30]. However, little is known about the mechanisms of fibroblast subpopulations underlying the progressive fibrosis of HP. Here, we identified ACTA2 ^high^ fibroblasts, COL1A1^high^ fibroblasts, TCF21^high^ fibroblasts and PLA2G2A^high^ fibroblasts. Moreover, we demonstrated the differentiation trajectories of COL1A1^high^ fibroblasts to ACTA2^high^ fibroblasts or PLA2G2A^high^ fibroblasts. COL1A1^high^ fibroblasts and PLA2G2A^high^ fibroblasts were related to EMT, angiogenesis, and inflammatory responses. ACTA2^high^ fibroblasts were enriched with myogenesis. TCF21^high^ fibroblasts showed greater activity of adipogenesis and glycolysis. Thus, our findings showed ACTA2^high^ fibroblasts, COL1A1^high^ fibroblasts, and PLA2G2A^high^ fibroblasts drives progressive fibrosis in HP.

Immune-checkpoint inhibitor associated HP has been identified as potentially severe events through inhibiting the cytotoxic T-lymphocyte antigen (CTLA)−4 pathways and programmed cell death protein 1 (PD1) in patients with cancer [31–34]. CD28/B7 antagonist decreases the extent of lung damage and inflammatory cell infiltrations, and affects the HP progression and the lung T cell subset kinetics in mice [35]. The results indicate that T cells and immune-checkpoint pathway may be related to HP pathogenesis. Previous work on Tregs and γδ T cells exhibited different transcriptional changes in response to regenerative or fibrogenic environmental causes [36]. We found that CHP had higher abundance of regulatory T cells, γδ T cells, GZMK^+^ CD8^+^ effector memory T cells, KLRG1^+^ effector CD8^+^ T cells through ssGSEA in GSE150910 databases. KLRG1^+^ effector CD8^+^ T cells showed developmental plasticity through regulating KLRG1 [37]. Here, AUCell analysis suggested that NK cells and KLRG1^+^ CD8+ effector cells showed greater enrichment of Inflammation-promoting signature. Tregs were related to inflammatory responses with low inflammation-promoting activity. γδ T cells were related to chemokine-production with higher expression levels of LTB, IL32, TYMP, SA100A6, CCL14, and CXCL13. Subsequently, integrating analysis further confirmed the higher abundance of Tregs and γδ T cells in CHP and its clinical implications.

**Fig. 8:**
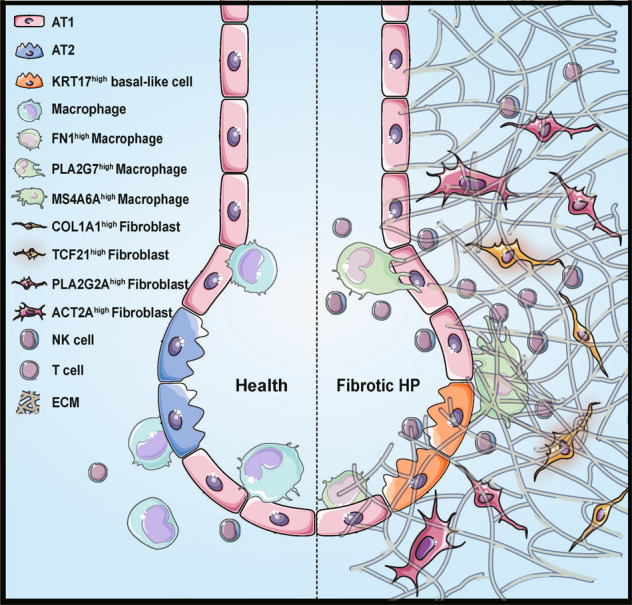
Dynamic cellular changes during FHP. We identified unique cell subpopulations including KRT17^high^ basal-like epithelial cells, FN1^high^ macrophages, PLA2G7^high^ macrophages, MS4A6A^high^ macrophages, ACTA2^high^ fibroblasts, COL1A1^high^ fibroblasts, TCF21^high^ fibroblasts, and PLA2G2A^high^ fibroblasts in FHP lungs. We also observed an expansion of NK cells and CD8^+^ T cells during FHP. These subpopulations are potentially key players for FHP pathogenesis.

In conclusion, our study depicted the cell landscapes of fibrotic hypersensitivity pneumonitis (Fig. 8), highlighting the indispensable role of cell subpopulations in shaping the complexity and heterogeneity of FHP, and identified potential subtype-specific functions and hopefully achieve therapeutic for FHP patients.

## Materials and methods

### Specimens and ethical considerations

Written informed consent form was signed by the subject. The study protocols were approved by the Ethics approval Chengdu Third People’s Hospital Institutional Review Board, and all procedures complied with all relevant ethical regulations. Fresh tissue samples from the lung lesion of the above-described patient were obtained directly from the operating room.

### Sample processing

Single cells were collected from lung tissues as described previously [10]. Briefly, fresh lung tissues were minced to smaller pieces of less than 1 mm in the RPMI 1640 cell culture medium (Gibco) with 10% heat inactivated fetal bovine serum (FBS, Gibco), and enzymatically digested with mix of Dispase II, Pronase, Collagenase A, Collagenase IV, Elastase, and DNase I for 1 h on shaker at 37 °C. The dissociated cells were passed through a 70 μm Cell-Strainer (BD). After removal of the supernatant by centrifugation (350×*g*, 10 min) and depletion of erythrocytes in the pellet with RBC lysis buffer (Thermo Fisher Scientific), cells were counted and critically assessed for cell viability and separation.

### 10× Genomics single-cell RNA/TCR library preparation and sequencing

scRNA-seq library was generated using the Chromium Single Cell 5′ Reagent Kits v2 (10× Genomics) according to the manufacturer’s instructions and targeting 8000–15,000 cells, and coupled scTCR-seq library was generated using Chromium Single Cell V(D)J Enrichment Kit, Human T Cell (10× Genomics). All the following steps were performed using the standard manufacturer’s protocols. cDNA library was sequenced on an NextSeq 2000 platform (Illumina).

### Single-cell RNA-Seq data processing

Low-quality reads were filtered out, and CellRanger v5.0.0 (10× Genomics) was applied to align reads and generate the gene-cell unique molecular identifier (UMI) matrix, using the human reference genome GRCh38. Seurat (version 4.0.3) in R (4.1.0) was used to screen out high-quality cells (500–5000 genes, 1600–25,000 UMIs, and mitochondria content less than 15%) for further analyses, which excluded most of empty wells or doublet cells [38].

### Single-cell RNA-Seq data collection

Single-cell RNA-Seq raw matrices of a total of 11,445 cells from a recent published study on human pulmonary fibrosis [10], GSE122960, covering lung tissues from two lung transplant donors (8761 cells) and a patient with FHP (2684 cells) were accessed from the GEO public database. Low-quality data were excluded using Seurat as described above.

### Integrated single-cell transcriptomic analyses

After normalization with NormalizeData(), FindVariableFeatures() was applied to identify highly variable genes in each sample. Integration was performed by FindIntegrationAnchors() and IntegrateData() function in Seurat with default options. Integrated data was scaled by the ScaleData() function. Principal components analysis and clustering were conducted with 30 PCs and a resolution of 0.8 in FindClusters(). For visualization, the dimensionality was reduced using the t-Distributed Stochastic Neighbor Embedding (t-SNE) with RunTSNE().

### Single-cell differential gene expression analyses and functional enrichment analyses

Single-cell differential gene expression analyses were conducted in MAST R package [39]. Likelihood ratio tests between the full and reduced model formulas were conducted to find DEGs. Multiple testing corrections were performed using Benjamini & Hochberg method. Genes with FDR < 0.05 were identified as DEGs. FindMarkers() and FindAllMarkers()function in Seurat were used to calculate cluster-specific marker genes (min.pct = 0.3, logfc.threshold = 0.25). The clusterProfiler package in R was used to conduct GO or KEGG enrichment analyses.

### Collection of gene signatures and AUC analyses

Cell type gene signatures and hallmark gene signatures were collected from Molecular Signatures Database (http://www.gsea-msigdb.org/), and Immune-related gene signatures were collected from ImmPort Portal (https://www.immport.org/). M1 and M2 macrophage signatures, Cytokines/Chemokines/Receptors signature, HLA signature, Inflammation-promoting signature, MHC signature, and Parainflammation signature are available in supplementary files. GSVA were performed with the GSVA R package v 1.40.1. Calculation and visualization of AUC values were performed via AUCell package v1.14.0 in R.

### Pseudotime trajectory analyses

Monocle R package v 2.20.0 was used for inferring the pseudotime trajectories [13]. We used high variable gene sets between cell clusters detected by Seurat to build pseudotime trajectories. Single cells were projected onto trajectory trees after reducing dimension via method of DDRTree and denoting pseudotime states with orderCells() function. Branch-dependent gene expression patterns were analyzed by BEAM() function.

### RNA velocity analyses

Velocyto R package was used to calculate RNA velocity values for each gene by recounting the spliced and unspliced reads with aligned bam files.

### Cell interaction analyses

NicheNet method was applied for inferring cell communication networks based on official workflow of nichenetr R package [22]. DEGs in receivers between FHP and control lungs were used to predict the top 20 potential regulatory ligands, their target genes on receivers, as well as the possible cell types as sources of ligand expression.

### Transcription factor module analyses

We used SCENIC method to predict active transcription factor modules [14]. After downloading transcription factor motif RcisTarget database for the hg38 human reference genome and excluding genes not in the database, gene coexpression modules were inferred by the R package GENIE, and transcription factor network analysis was implemented in SCENIC R package. Then, regulon activity scoring for TF modules and visualization were performed via AUCell package in R.

### Single-cell TCR-seq analyses

Raw sequencing files were aligned and annotated by using CellRanger v5.0.0 (10× Genomics). After removal of cells lacking a TCR alpha or TCR beta chain, or expressing two or more TCR alpha/TCR beta chains. TCR clonotypes were assigned based on the CDR3α and CDR3β nucleotide sequences.

### Bulk RNA-seq data collection and analyses

Bulk RNA-seq data from GSE150910 with lung samples from chronic hypersensitivity pneumonitis (*n* = 82) and healthy controls (*n* = 103) were accessed from the GEO data repository [40]. Differential expression analysis was implemented in the R package DESeq2 [41]. ssGSEA were performed with the GSVA R package v 1.40.1.

### Immunofluorescence staining

Immunofluorescence staining was done as described previously [42]. Paraffin-embedded lung tissues were sliced into 5 µm slides using standard histological methods. After dewaxing and antigen retrieval, sections were blocked with 10% goat serum (Thermo Fisher Scientific) for 45 min at 37 °C. Specimens were then incubated with primary antibodies including CD68(Affinity, DF7518, 1:200), PLA2G7(Proteintech, 15526-1-AP, 1:200), MS4A6A(Bioss, bs-13692R, 1:200), ACTA2(Affinity, AF1032, 1:200), AGER(Affinity, AF5309, 1:200), TCF21(Affinity, DF13477, 1:200), SFTPC(Affinity, DF6647, 1:200), KRT17(Abbkin, ABM0032, 1:200), and COL1A1(Affinity, AF7001, 1:200) for 1 h at 37 °C. Then the corresponding secondary antibodies including Alexa Fluor ® 488 goat anti-mouse IgG (H&L, 1:1000), Alexa Fluor ® 555 goat anti-Rabbit IgG (H&L, 1:1000) or Alexa Fluor ® 649 goat anti-Rabbit IgG (H&L, 1:1000) were incubated for 1 h at 37 °C, and cell nucleus were stained with 4′-6-diamidino-2-phenylindole dihydrochloride (DAPI) (Biosharp). After using Vector® True VIEW™ Autofluorescence Quenching Kit (Vectorlabs) to diminish autofluorescence, the stained slides were photographed using laser scanning confocal microscope (Olympus), and analyzed with CellSens Dimension Software (Olympus).

### Statistical analysis

The Wilcoxon test was used to compare between two groups. One-way ANOVA was used for comparison of more than two groups. *P* values < 0.05 were considered statistically significant.

## Supplementary information

Supplementary table2-gene signatures

Supplementary file 1-case presentation

Supplementary figure 1

Supplementary figure 2

Supplementary figure 3

Supplementary figure 4

Supplementary figure 5

Supplementary figure 6

Supplementary figure 7

Supplementary figure 8

Supplementary figure 9

Supplementary figure 10

Supplementary figure 11

Supplementary figure 12

Supplementary figure 13

Supplementary figure 14

Supplementary figure 15

Supplementary figure 16

Supplementary figure 17

Supplementary figure 18

Supplementary figure 19

Supplementary figure 20

Supplementary figure 21

Supplementary figure and table legends

Supplementary figures 1-21

Supplementary table1-M1 and M2 signatures

## Data Availability

Some or all data, models, or code generated or used during the study are available from the corresponding author by request.
