# Supplementary material for: Characterizing cellular heterogeneity in fibrotic hypersensitivity pneumonitis by single-cell transcriptional analysis
Source: Cell Death Discov. 2022 Jan 28;8:38. doi: 10.1038/s41420-022-00831-x (PMC8795750; doi:10.1038/s41420-022-00831-x)
Supplement: Supplementary file 2 — Supplementary file 1-case presentation [file 41420_2022_831_MOESM2_ESM.pdf]

Case presentation

A 73-year-old female non-smoker presented with activity-related dyspnea and chest distress for 1 month, and the symptoms could relieve at rest. Two weeks later, she was admitted to our hospital with worsening dyspnea and cough productive with small amounts of yellow sputum. The patient had no fever or rash, and no joint pain, joint swelling, limited motion or morning stiffness. She had no significant medical history, and denied environmental, chemical, and occupational exposures or history of pet contact. Findings from a physical examination were notable for Velcro rales in bilateral lungs. Chest computed tomography revealed diffuse ground-glass opacification, mosaic attenuation, interlobular septal thickening and reticulation in both lungs. The hemogas analysis showed a type I respiratory failure. Lung function testing demonstrated severe decreases in carbonmonoxide diffusion capacity and small airways obstruction, while methacholine bronchial provocation test was negative. Laboratory tests showed an erythrocyte sedimentation (ERS) rate of 120 mm per hour (reference range, 0 to 20), and a serum Krebs Von den Lungen-6 (KL-6) level of 2763 U per microliter (reference range, 0 to 500). She was negative for procalcitonin, C-reactive protein, β-1,3-glucan, sputum cultures, and sputum acid-fast bacilli smears. Also, rheumatoid factor (RF), anti-cyclic peptide containing citrulline (Anti-CCP), antinuclear antibody (ANA) profile, antineutrophil cytoplasmic antibodies (ANCA), myositis specific autoantibodies (MSA), and creatine kinase (CK) were negative. The symptoms were alleviated slightly following treatments with levofloxacin, methylprednisolone, acetylcysteine, and pirfenidone for 12 days. As the disease progressed rapidly and the etiology remained unclear, a surgical lung biopsy using video-assisted thoracoscopy was decided upon after discussion with the patient. In the biopsy sections, we noted significantly dilated bronchioles with neutrophilic exudates and mucus. There was the appearance of squamous metaplasia in some bronchiole epithelia with peribronchiolar lymphocytic infiltrates, and many hyperplastic polypoid connective tissues in the lumen. Focal alveolar epithelial type II cell hyperplasia were observed and alveolar walls were thickened with infiltration of lymphocytes. Additionally, many fibroblastic foci were detected, and multinuclear

giant cells were seen occasionally. Collectively, the patient was ultimately diagnosed with secondary interstitial pneumonia and fibrotic hypersensitivity pneumonia according to the guidance of ATS/JRS/ALAT 2019.
