# Supplementary figures and images for: Characterizing cellular heterogeneity in fibrotic hypersensitivity pneumonitis by single-cell transcriptional analysis
Source: Cell Death Discov. 2022 Jan 28;8:38. doi: 10.1038/s41420-022-00831-x (PMC8795750; doi:10.1038/s41420-022-00831-x)

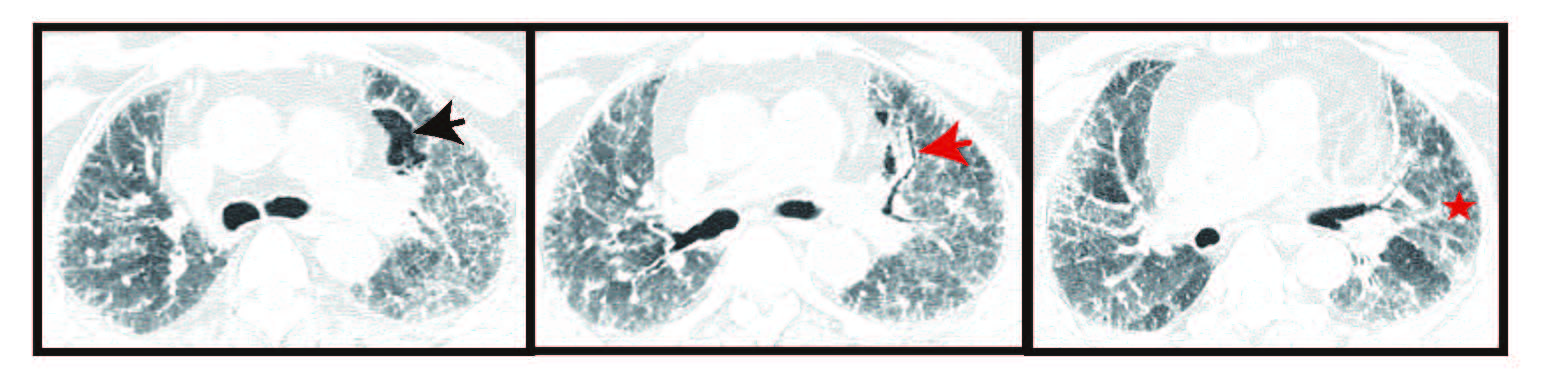

Supplement: Supplementary file 3 — Supplementary figure 1 [file 41420_2022_831_MOESM3_ESM.jpg]

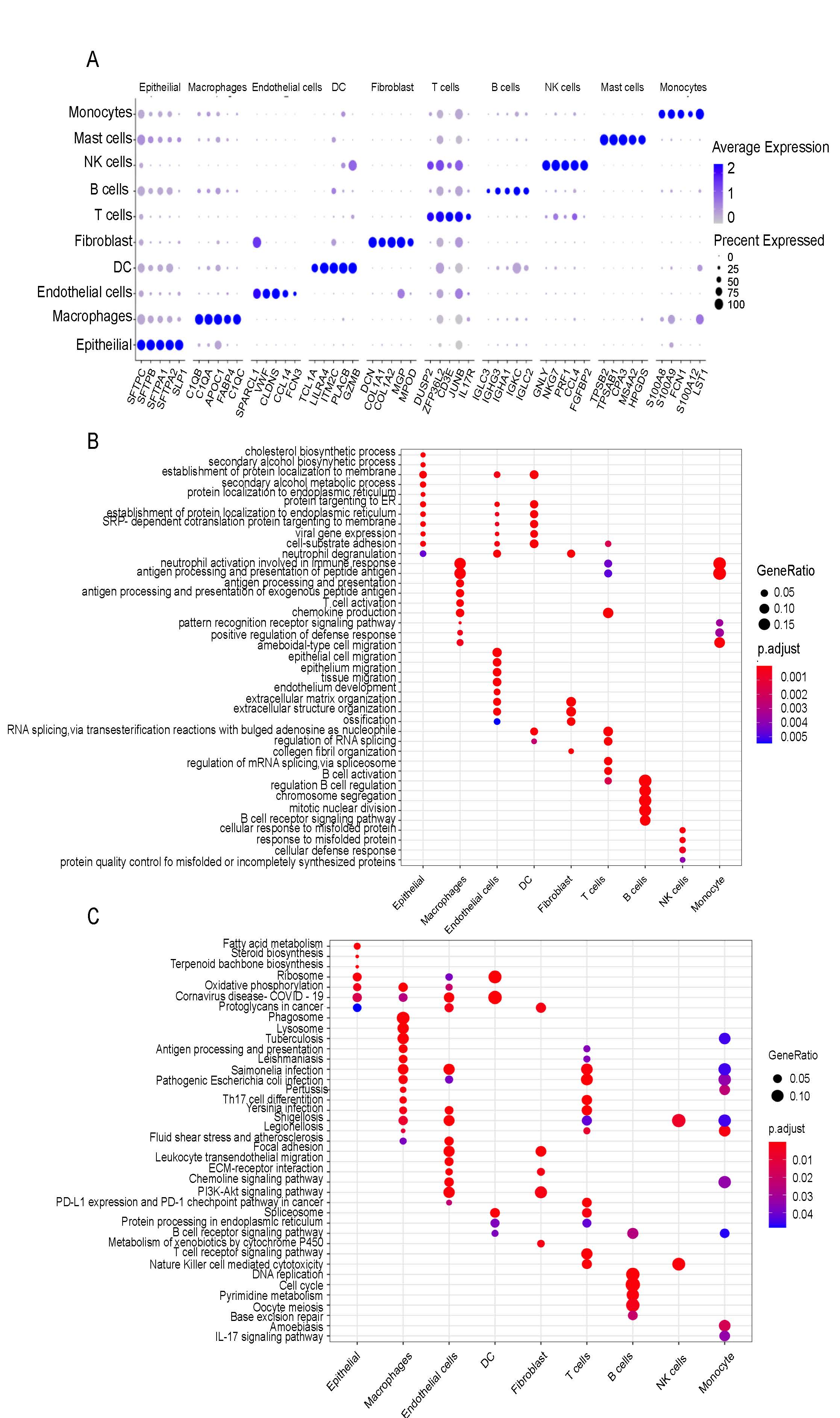

Supplement: Supplementary file 4 — Supplementary figure 2 [file 41420_2022_831_MOESM4_ESM.jpg]

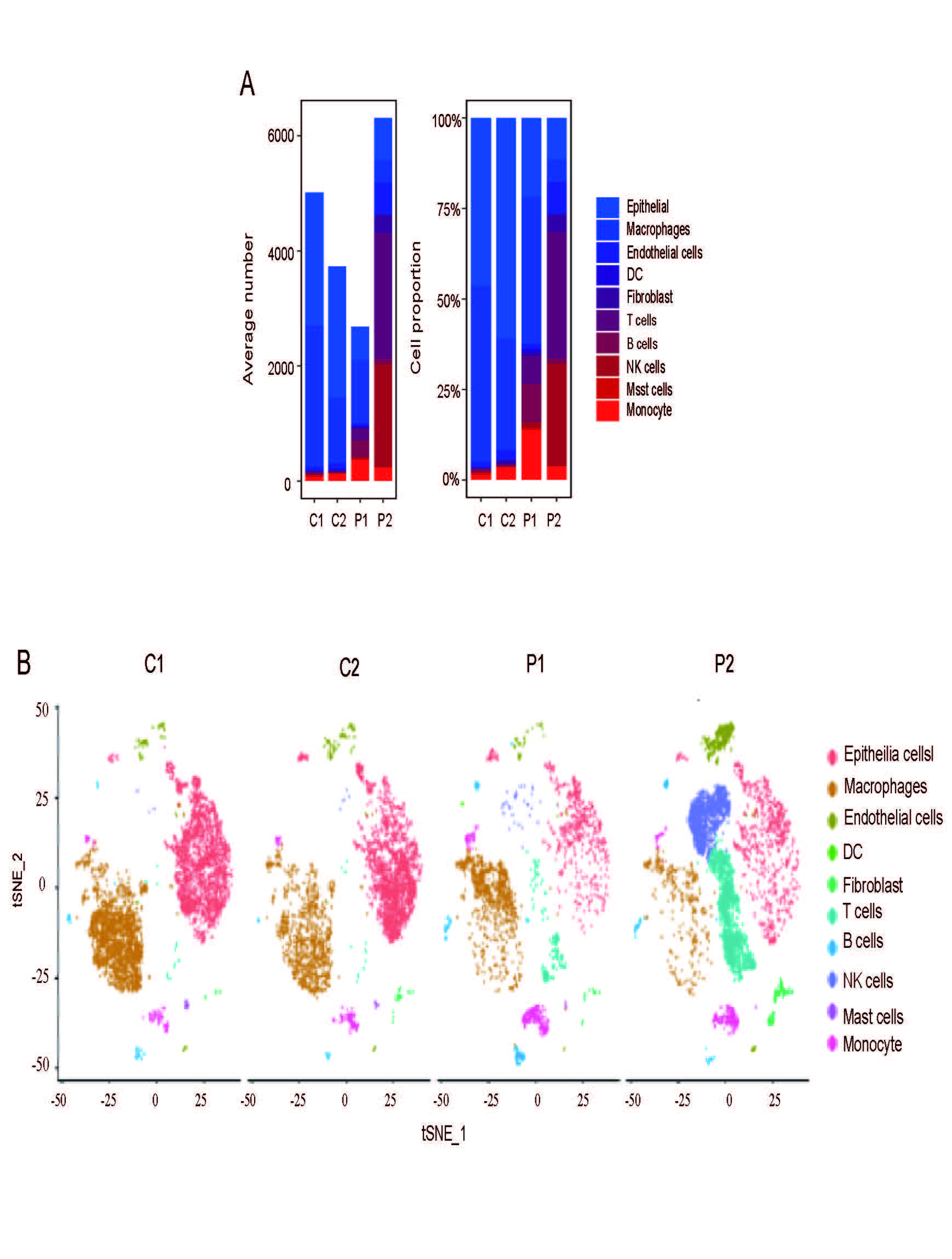

Supplement: Supplementary file 5 — Supplementary figure 3 [file 41420_2022_831_MOESM5_ESM.jpg]

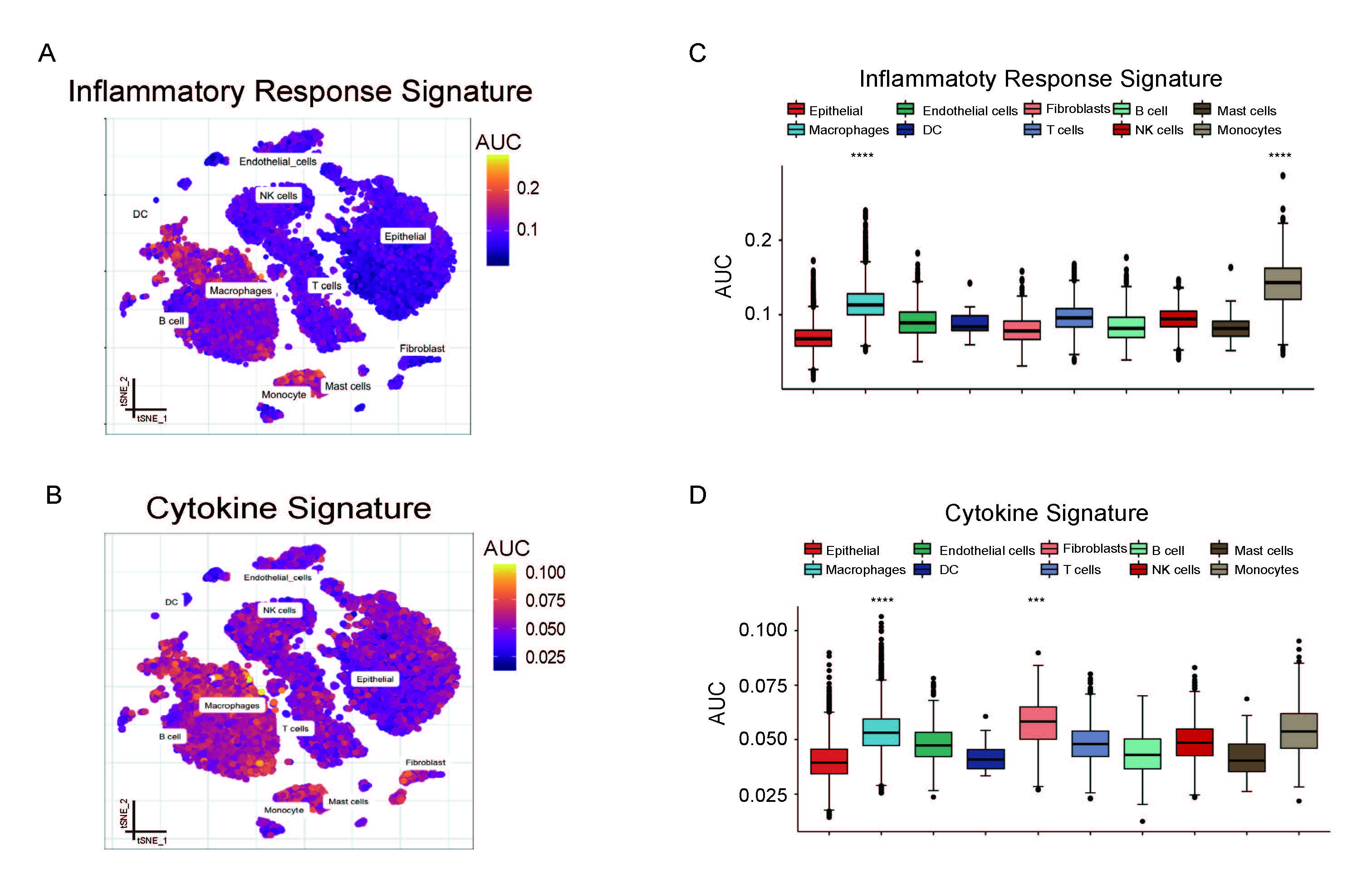

Supplement: Supplementary file 6 — Supplementary figure 4 [file 41420_2022_831_MOESM6_ESM.jpg]

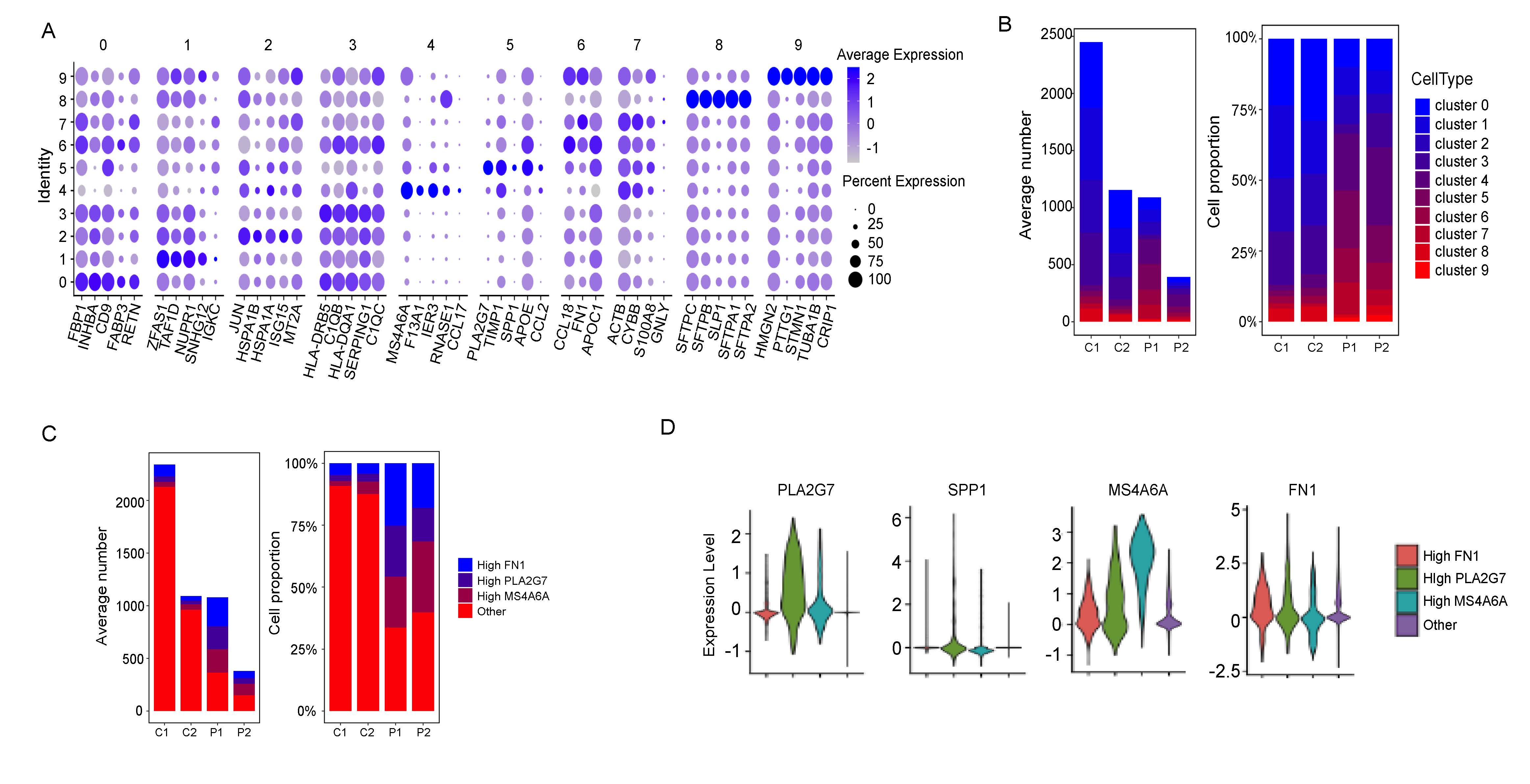

Supplement: Supplementary file 7 — Supplementary figure 5 [file 41420_2022_831_MOESM7_ESM.jpg]

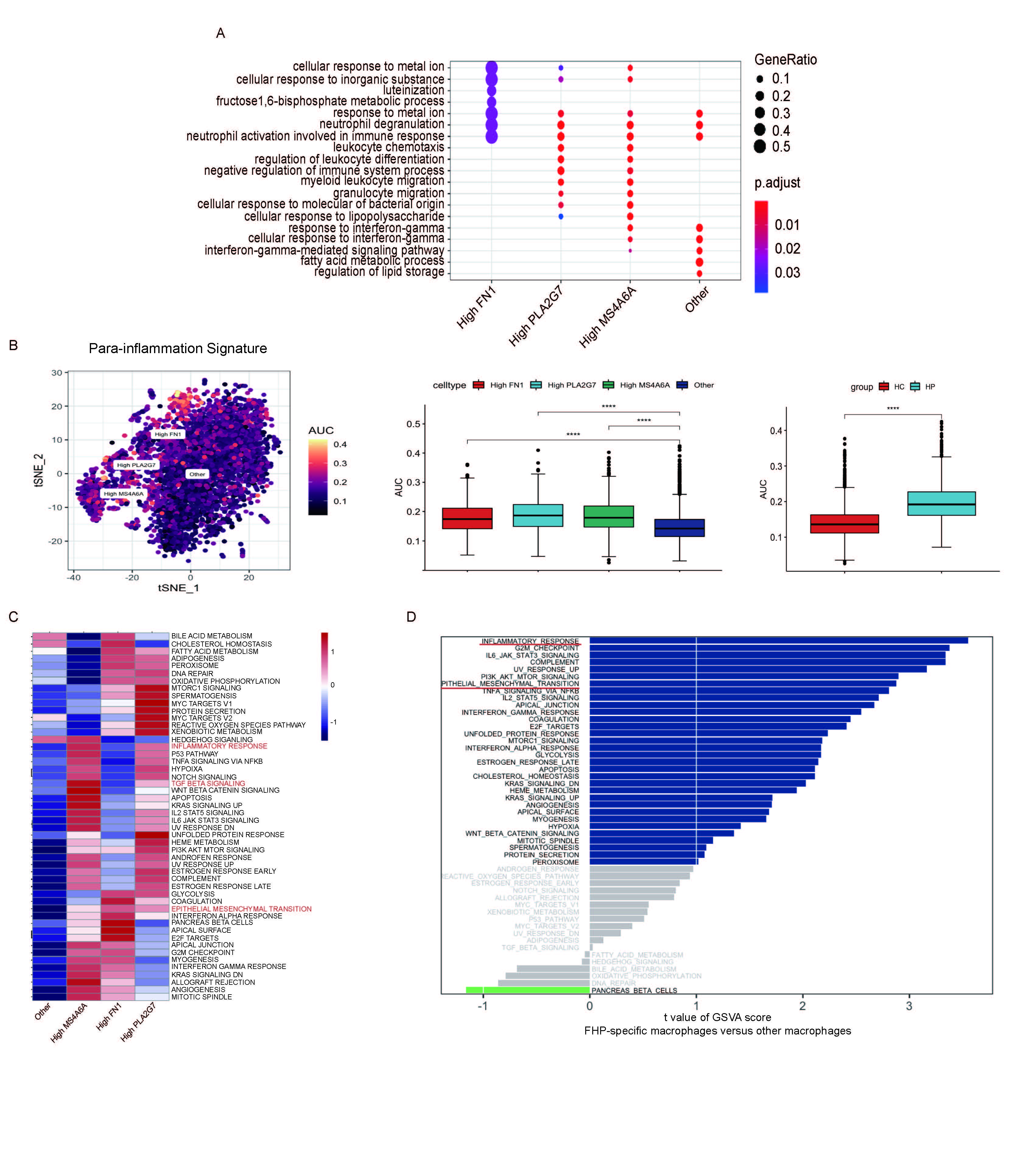

Supplement: Supplementary file 8 — Supplementary figure 6 [file 41420_2022_831_MOESM8_ESM.jpg]

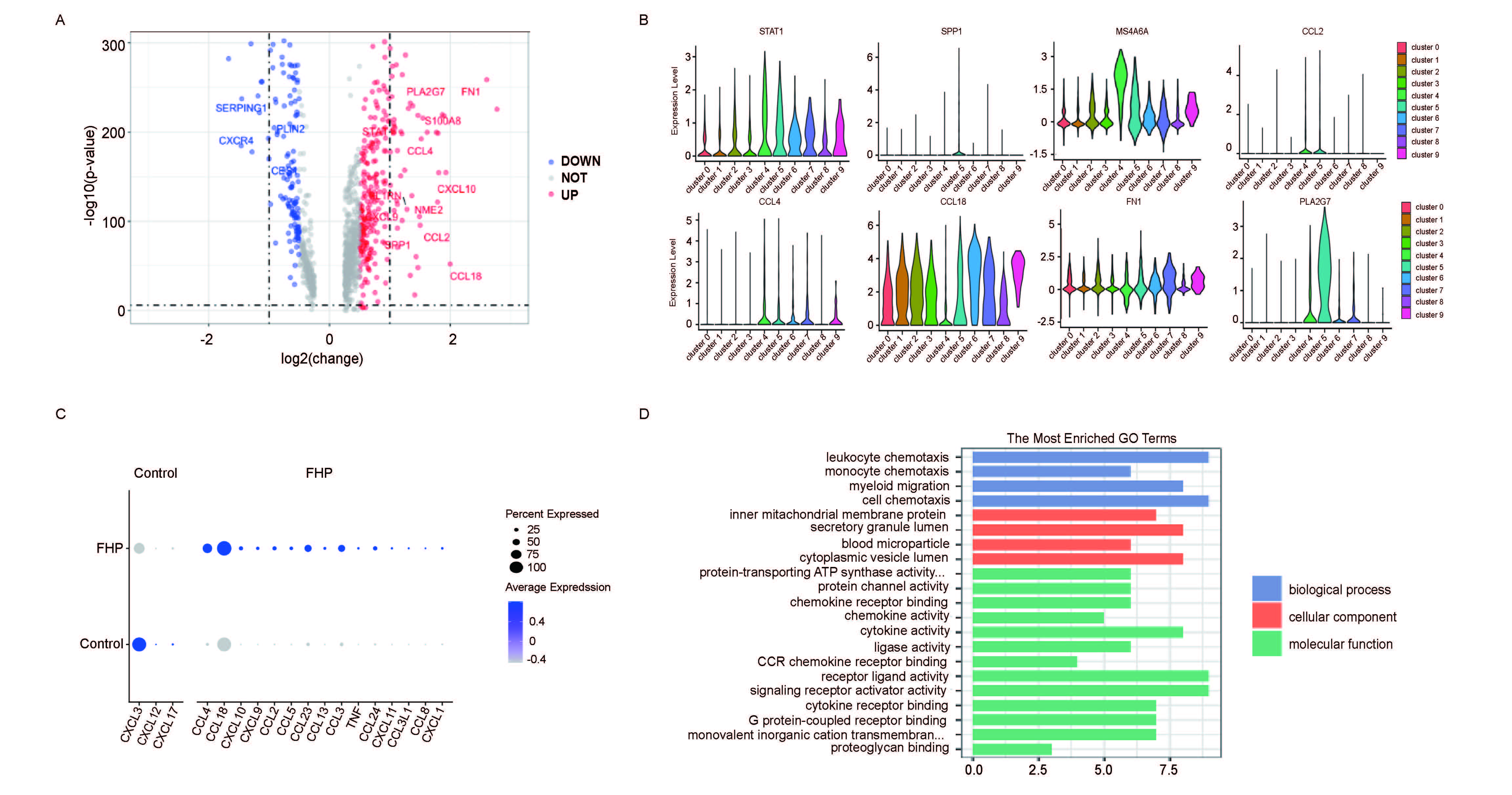

Supplement: Supplementary file 9 — Supplementary figure 7 [file 41420_2022_831_MOESM9_ESM.jpg]

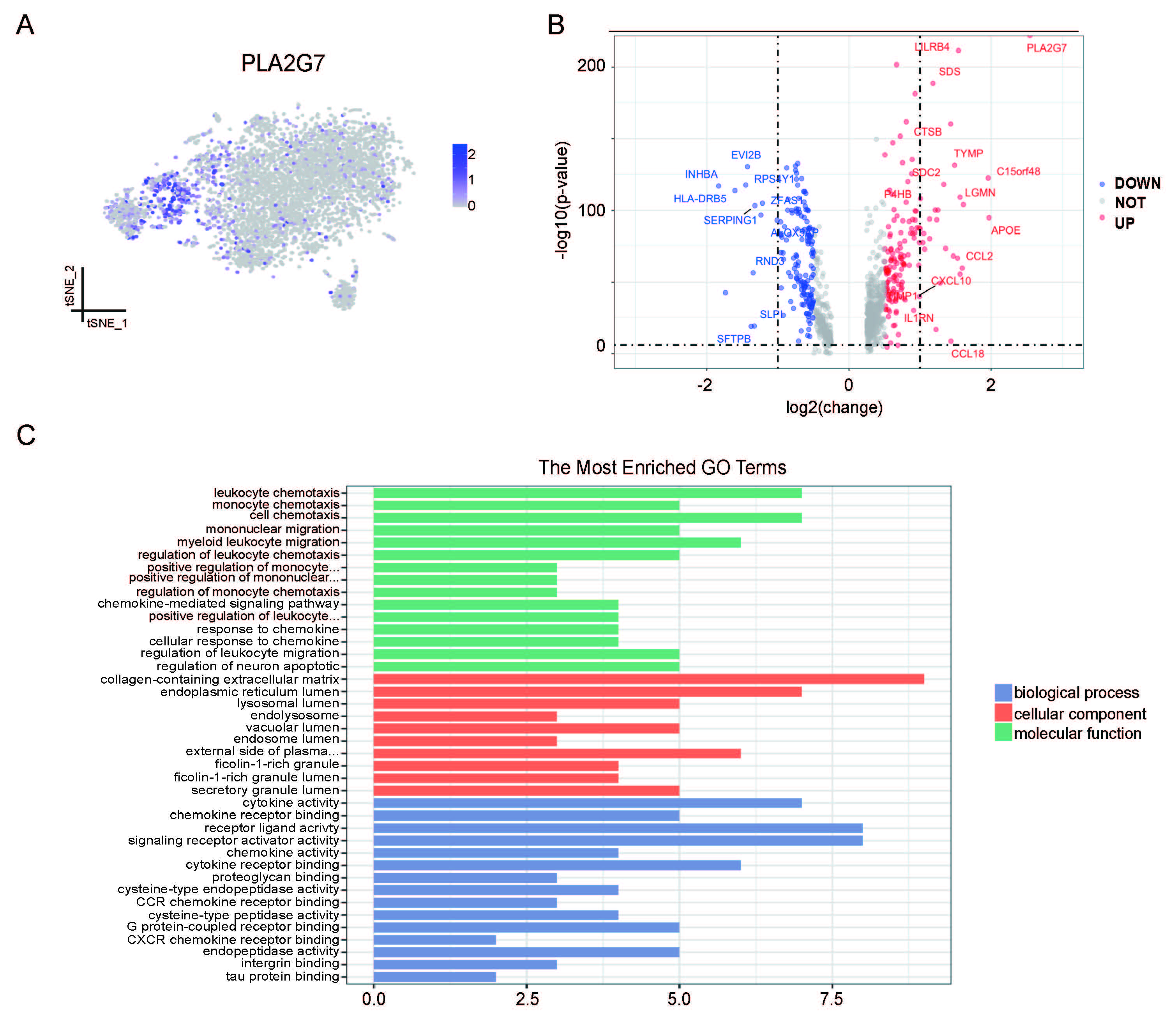

Supplement: Supplementary file 10 — Supplementary figure 8 [file 41420_2022_831_MOESM10_ESM.jpg]

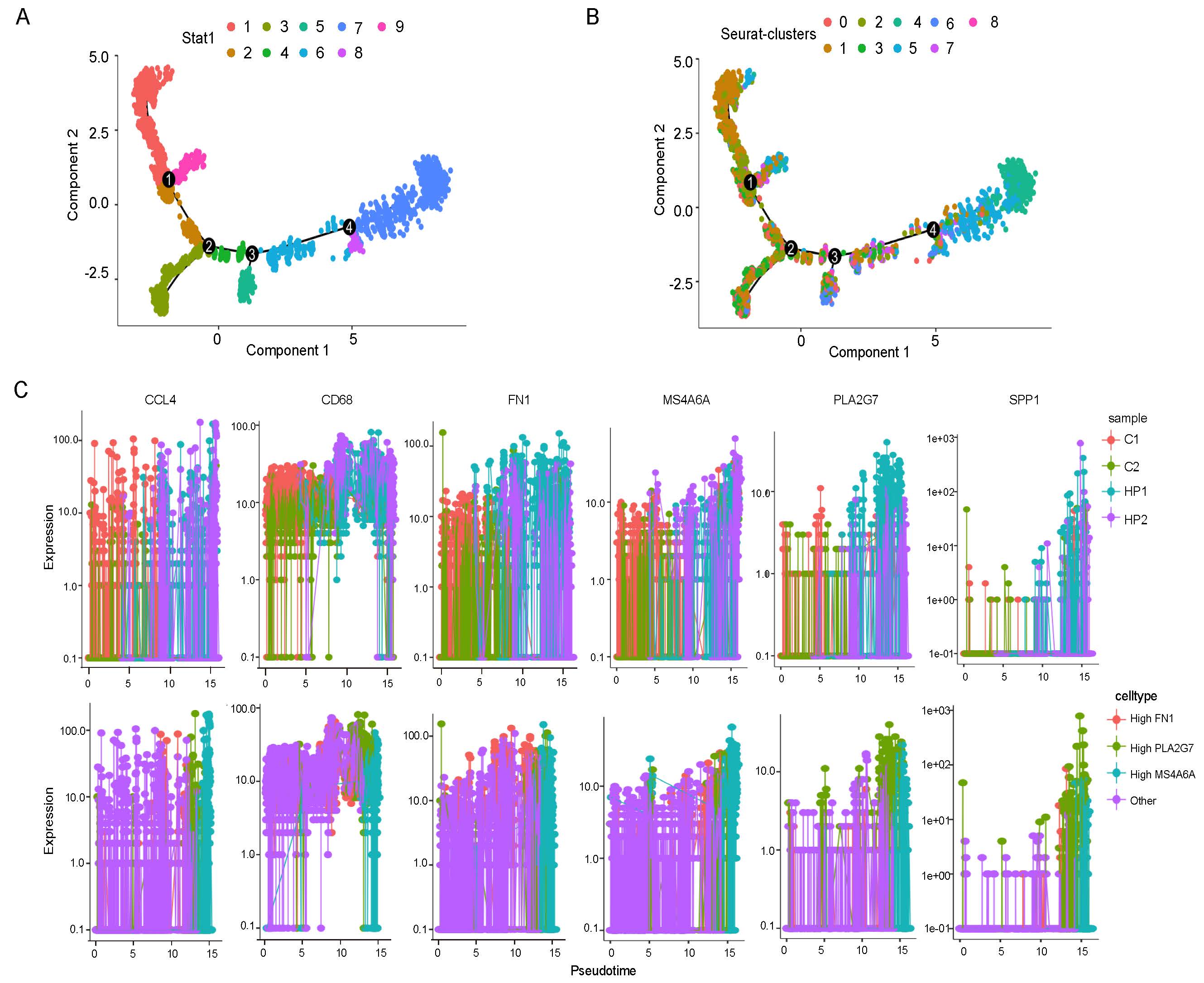

Supplement: Supplementary file 12 — Supplementary figure 10 [file 41420_2022_831_MOESM12_ESM.jpg]

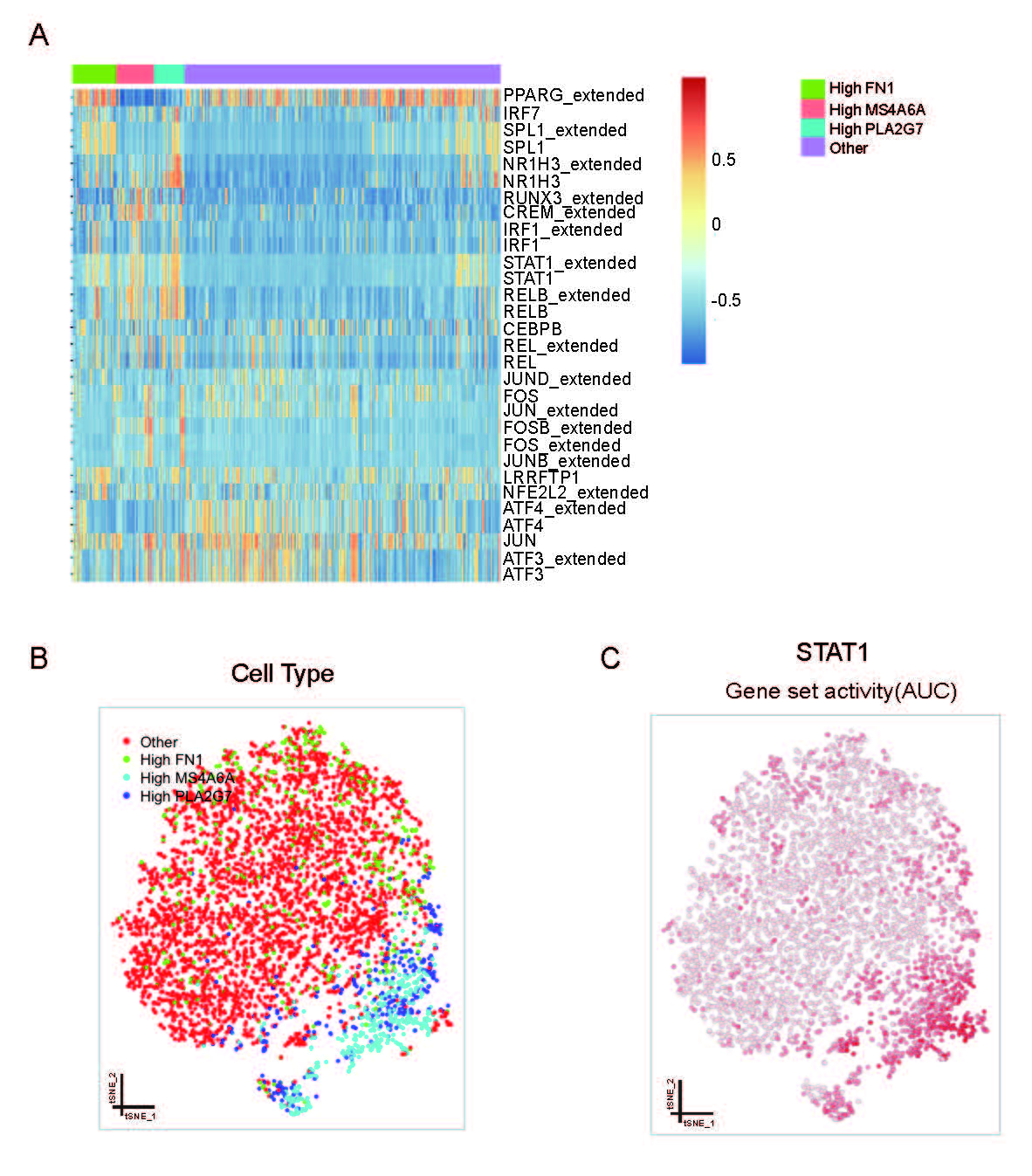

Supplement: Supplementary file 13 — Supplementary figure 11 [file 41420_2022_831_MOESM13_ESM.jpg]

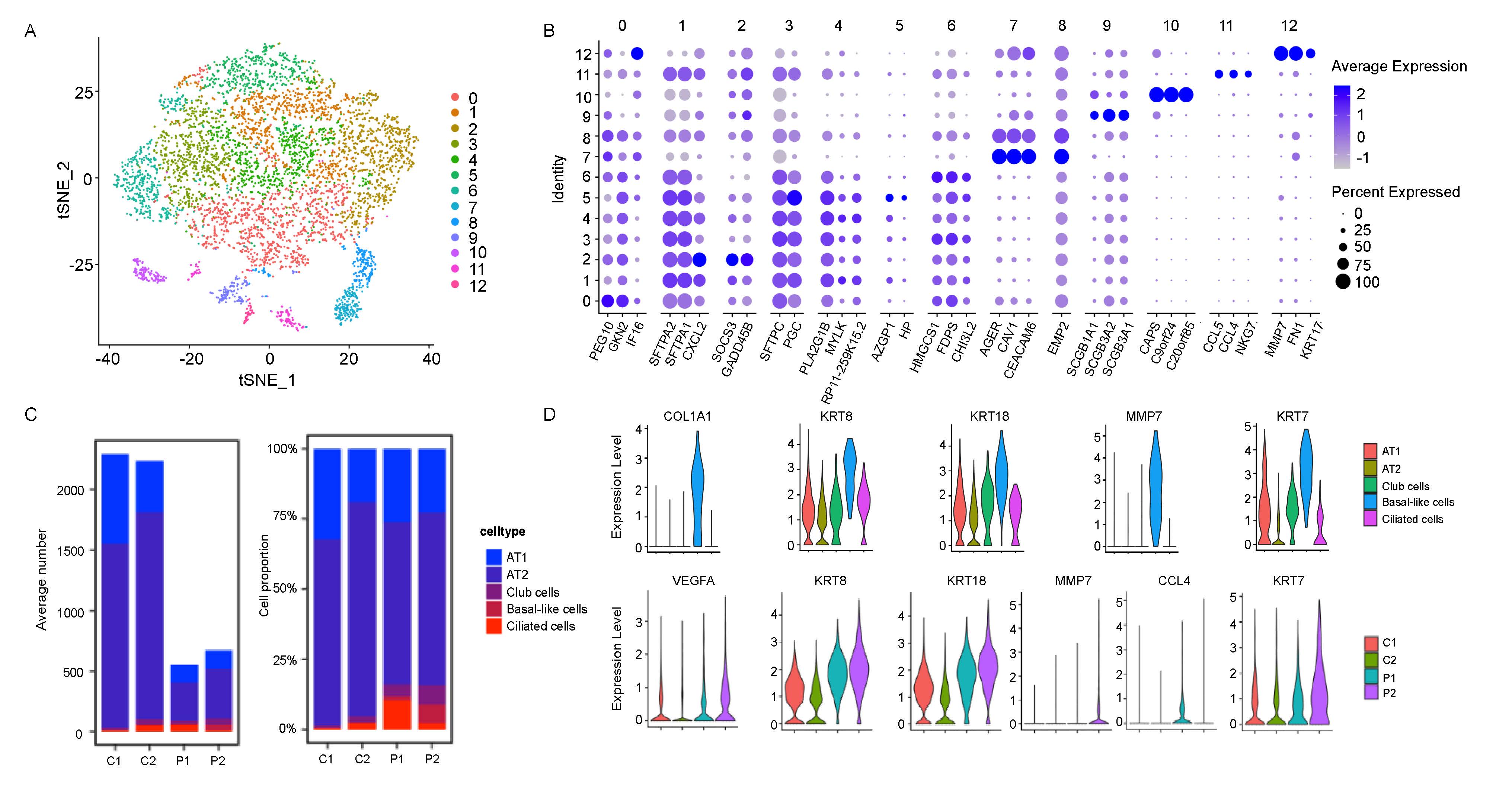

Supplement: Supplementary file 14 — Supplementary figure 12 [file 41420_2022_831_MOESM14_ESM.jpg]

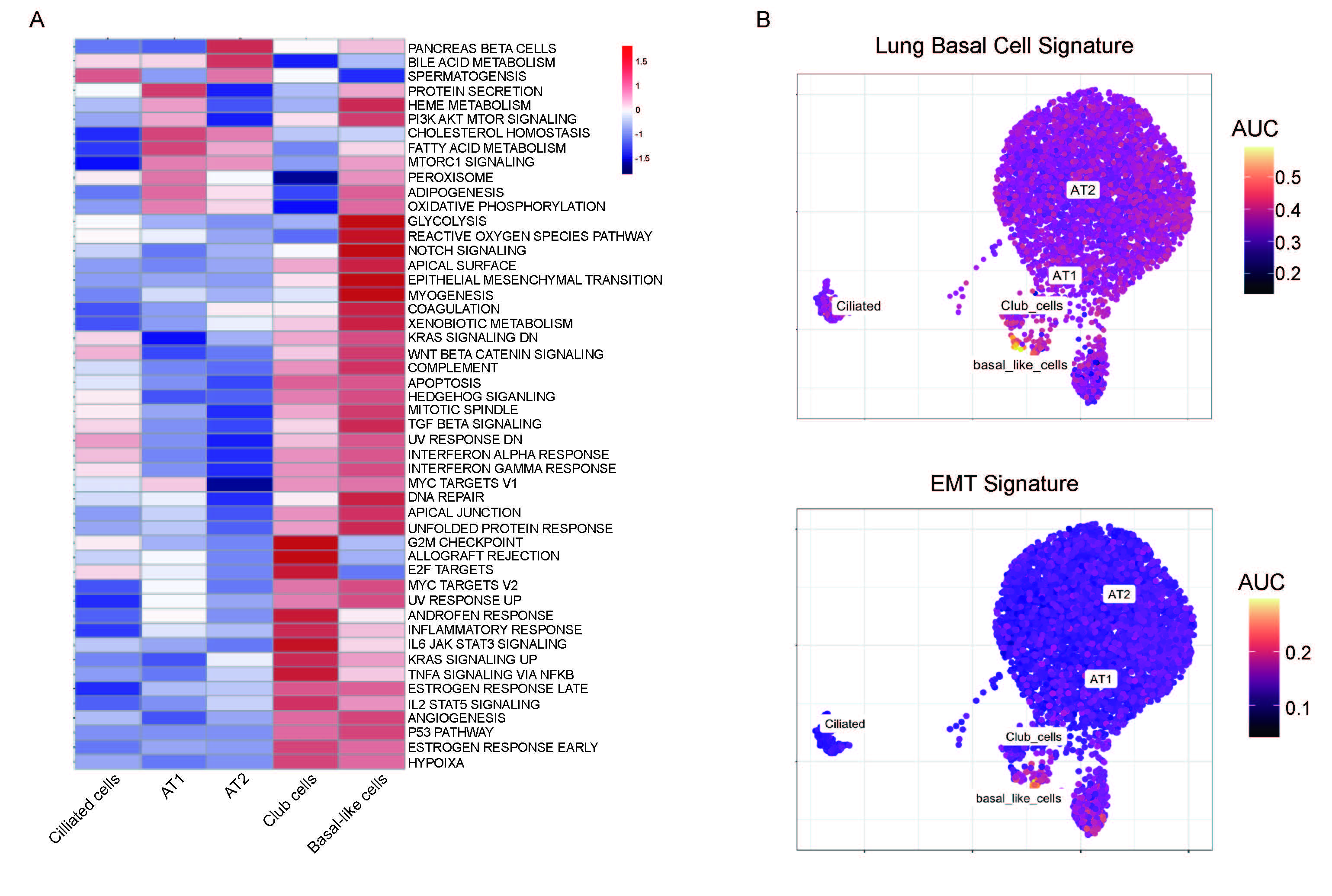

Supplement: Supplementary file 15 — Supplementary figure 13 [file 41420_2022_831_MOESM15_ESM.jpg]

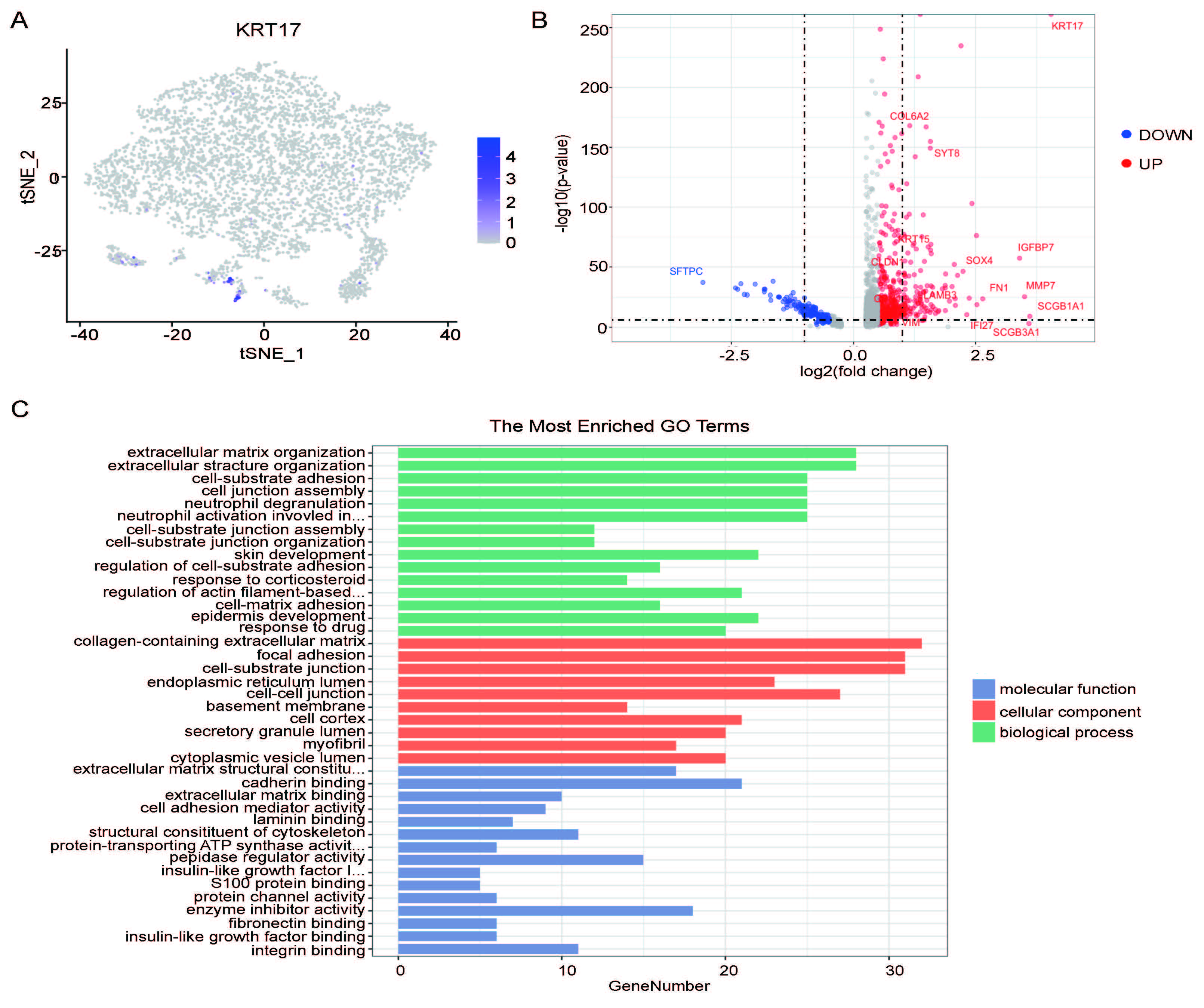

Supplement: Supplementary file 16 — Supplementary figure 14 [file 41420_2022_831_MOESM16_ESM.jpg]

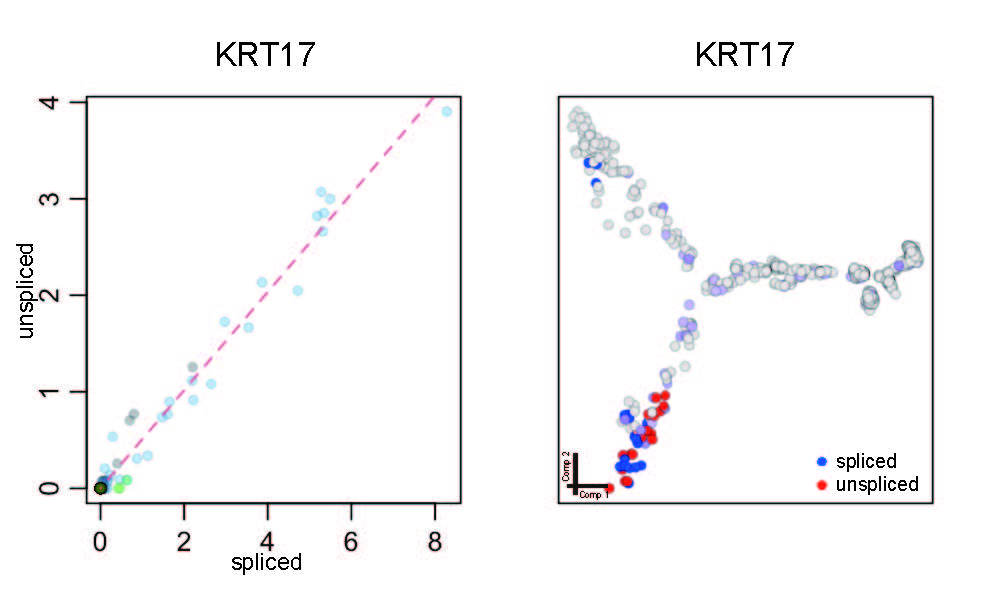

Supplement: Supplementary file 17 — Supplementary figure 15 [file 41420_2022_831_MOESM17_ESM.jpg]

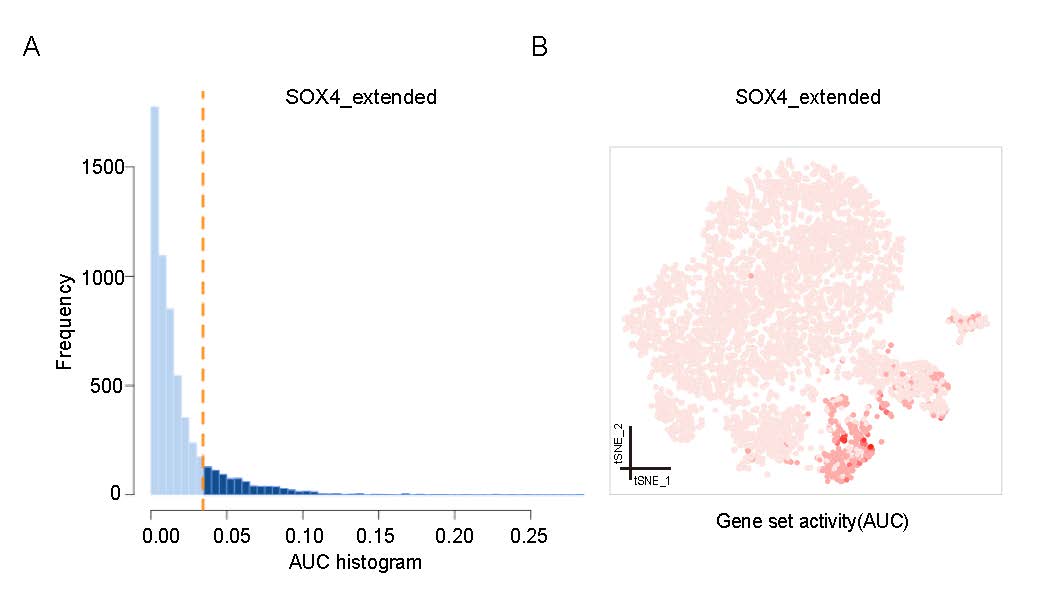

Supplement: Supplementary file 18 — Supplementary figure 16 [file 41420_2022_831_MOESM18_ESM.jpg]

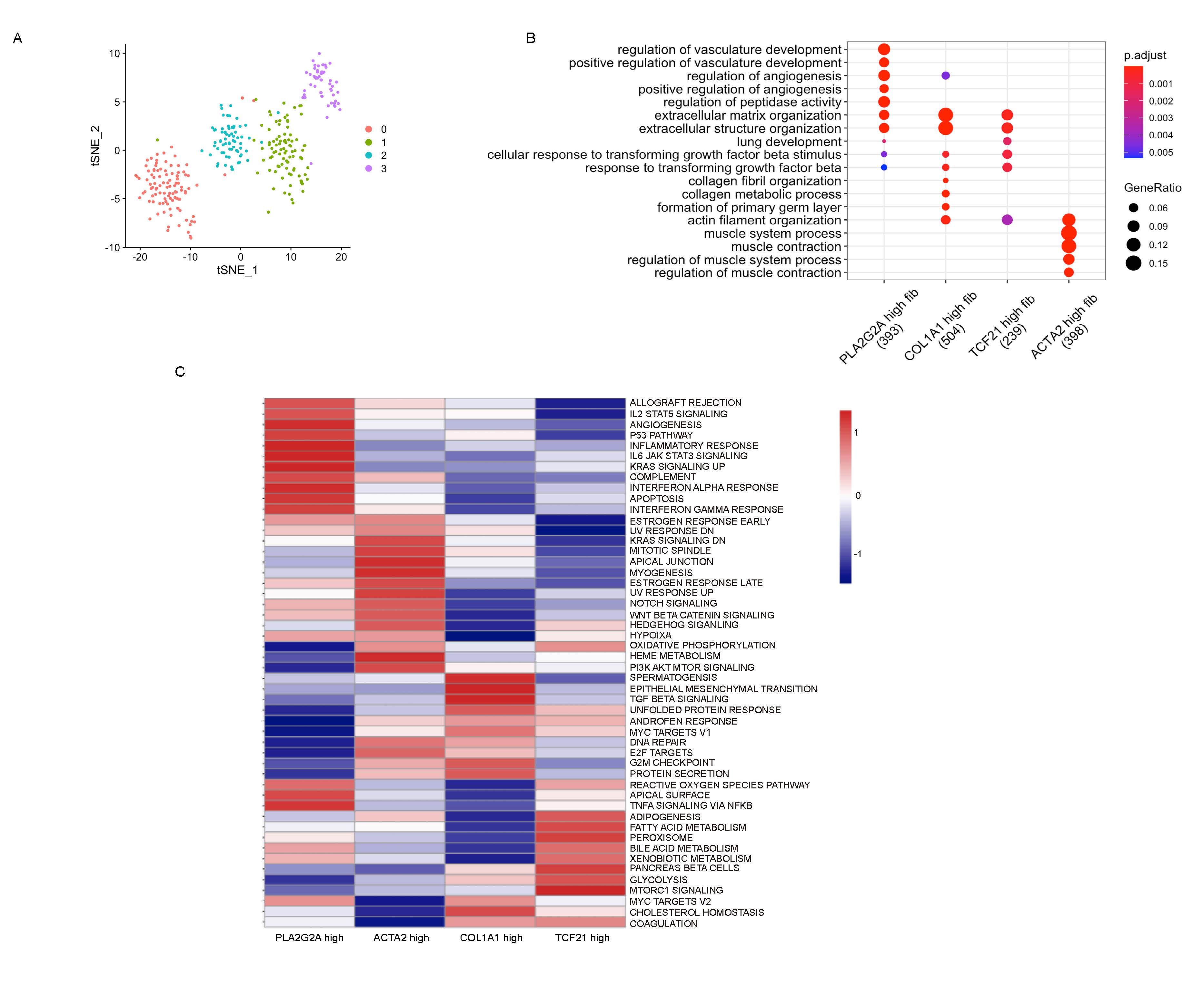

Supplement: Supplementary file 19 — Supplementary figure 17 [file 41420_2022_831_MOESM19_ESM.jpg]

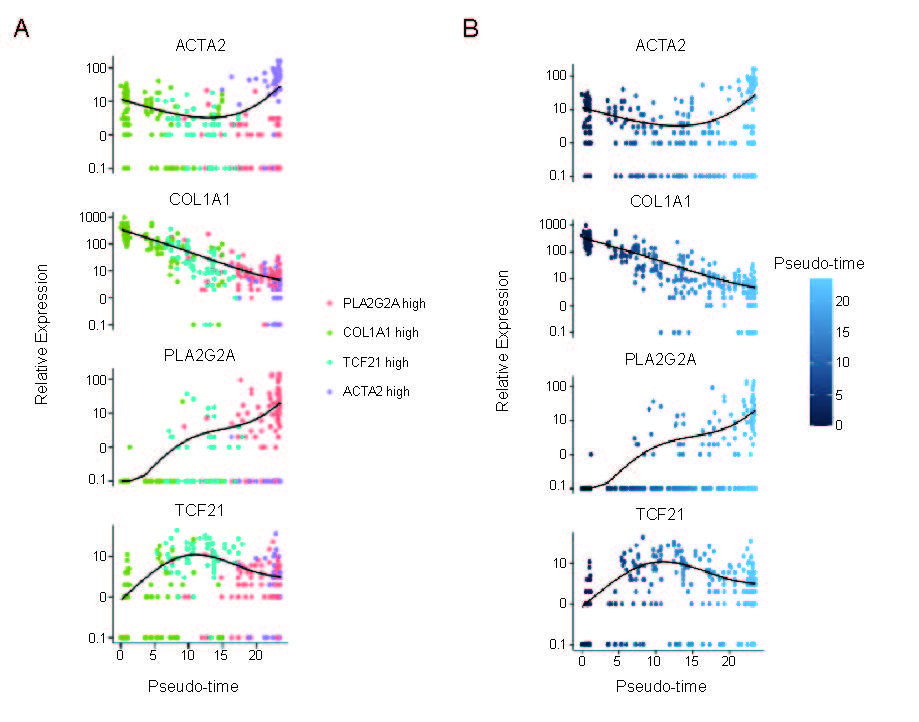

Supplement: Supplementary file 20 — Supplementary figure 18 [file 41420_2022_831_MOESM20_ESM.jpg]

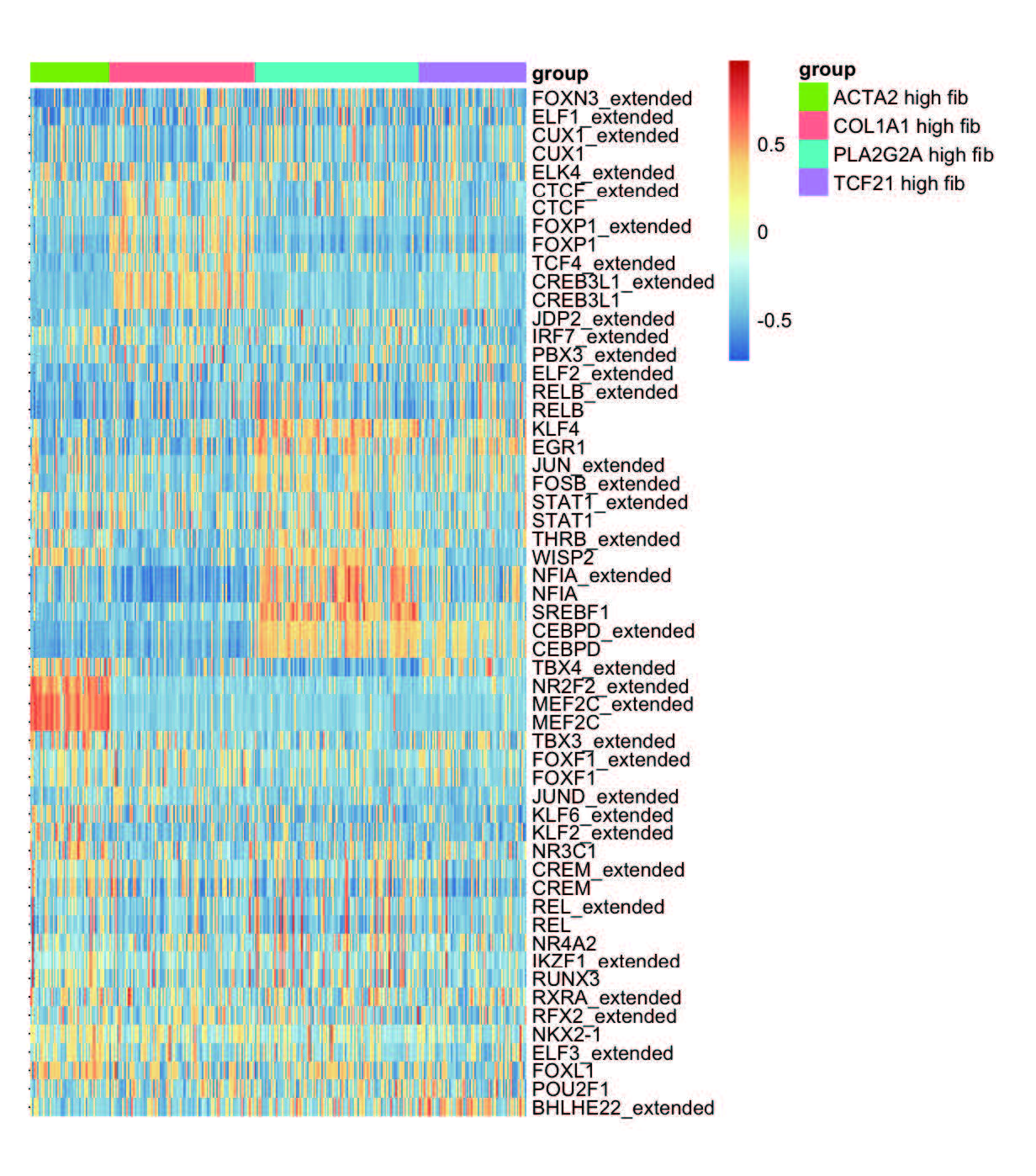

Supplement: Supplementary file 21 — Supplementary figure 19 [file 41420_2022_831_MOESM21_ESM.jpg]

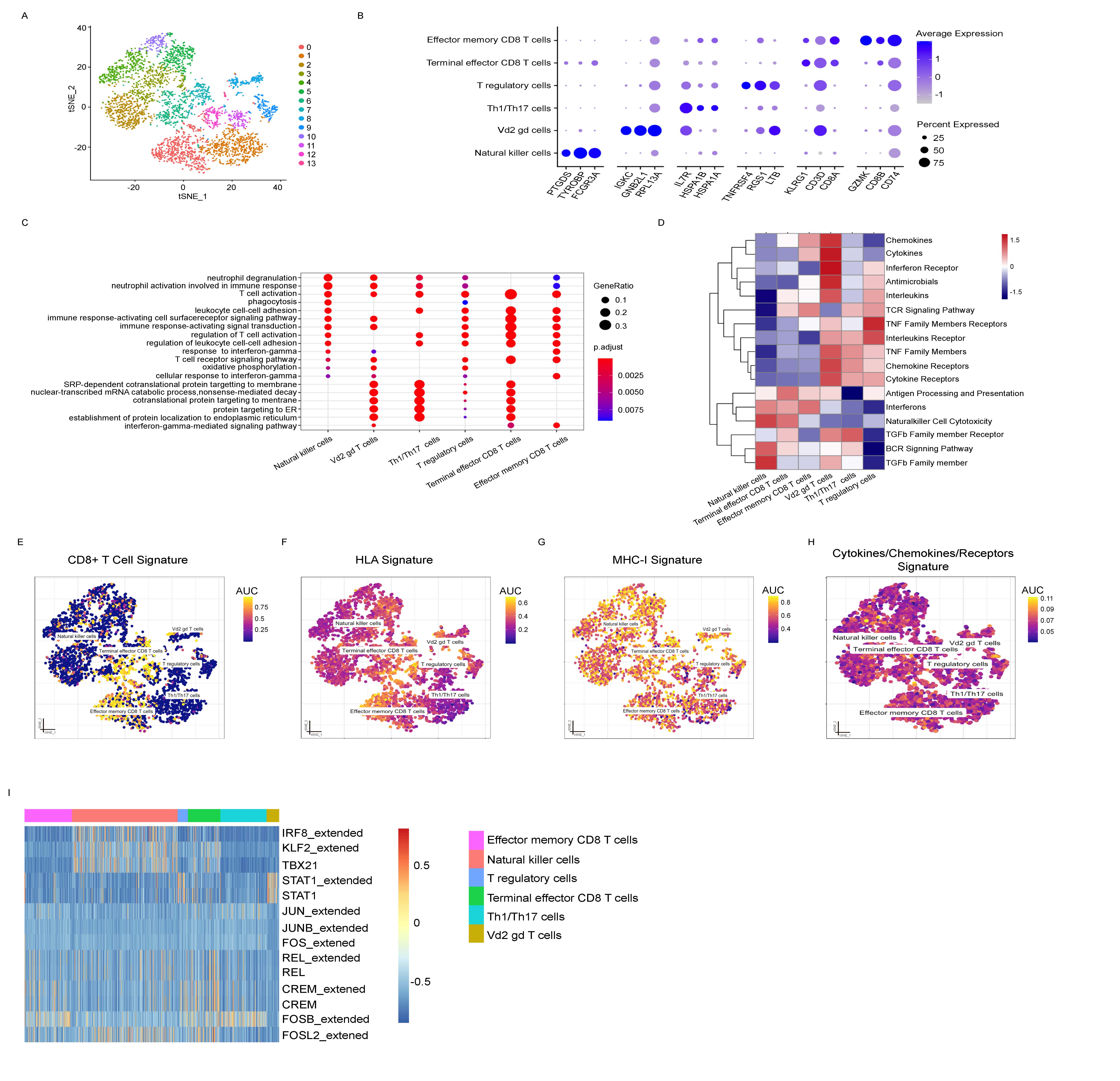

Supplement: Supplementary file 22 — Supplementary figure 20 [file 41420_2022_831_MOESM22_ESM.jpg]

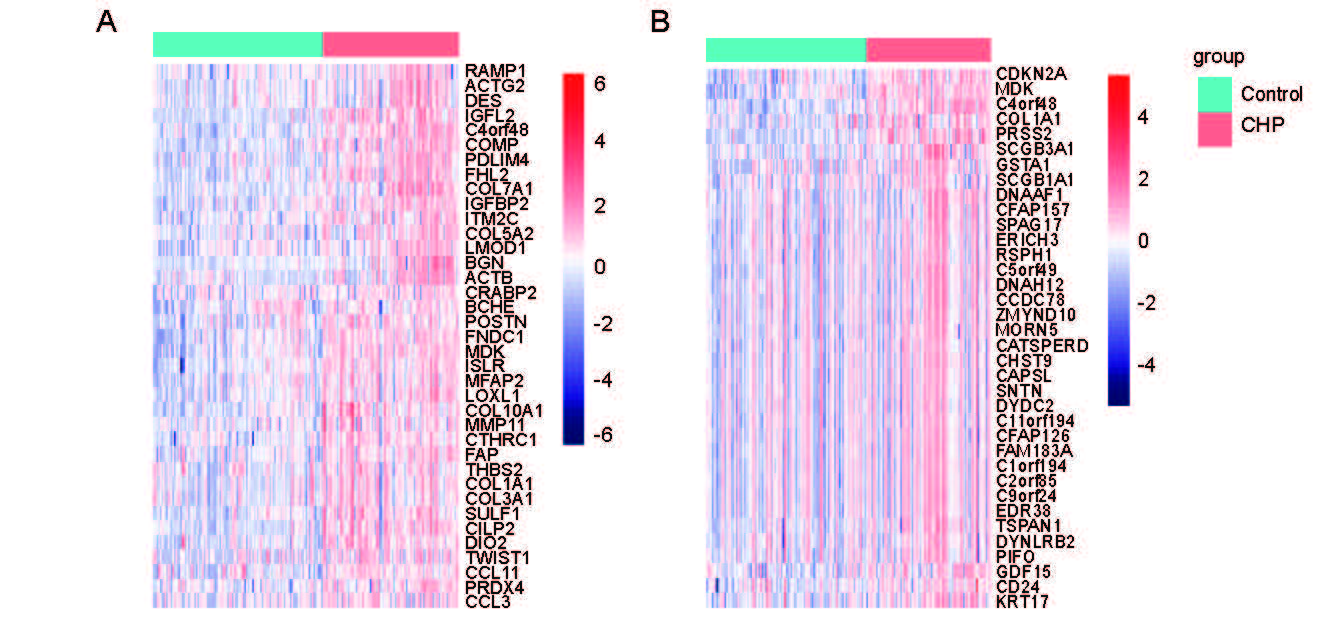

Supplement: Supplementary file 23 — Supplementary figure 21 [file 41420_2022_831_MOESM23_ESM.jpg]
