# Supplementary material for: Characterizing cellular heterogeneity in fibrotic hypersensitivity pneumonitis by single-cell transcriptional analysis
Source: Cell Death Discov. 2022 Jan 28;8:38. doi: 10.1038/s41420-022-00831-x (PMC8795750; doi:10.1038/s41420-022-00831-x)
Supplement: Supplementary file 24 — Supplementary figure and table legends [file 41420_2022_831_MOESM24_ESM.pdf]

Supplementary figure legends

Supplementary figure 1. Study design and single-cell transcriptional profiling of lung cells. Related to Figure 1. Chest computed tomography scan of the patient showing traction bronchiectasis (red arrow), interlobular septal thickening, reticulation, and 'three-density pattern' which consists of high attenuation area (red star), lucent area (black arrow), and normal area.

Supplementary figure 2. Study design and single-cell transcriptional profiling of lung cells. Related to Figure 1. (A) The expression levels of top 5 marker genes in cell types. (B-C) The bubble plots showing GO (B) and KEGG pathway (C) analysis of DEGs in each cell type.

Supplementary figure 3. Study design and single-cell transcriptional profiling of lung cells. Related to Figure 1. (A) Average cell numbers (left) and relative percentage (right) of cell types across samples. (B) The t-SNE maps showing the distribution of cell types across sample origins.

Supplementary figure 4. Study design and single-cell transcriptional profiling of lung cells. Related to Figure 1. (A-B) AUCell analyses of the relative gene set enrichment scores in Inflammatory Response signature(A), and Cytokine signature(B). (C-E) The box plots showing AUC scores of selected gene signatures in each cell type. *P<0.05, **P<0.01, ***P<0.001, ****P<0.0001.

Supplementary figure 5. Single-cell RNA-seq analysis reveals disease-specific macrophage subpopulations in FHP lungs. Related to Figure 2. (A) The expression levels of top 5 marker genes in subclusters. (B-C) Average cell numbers (left) and relative percentage (right) of cell subclusters (B) or macrophage subtypes (C) across samples. (D) Violin plots displaying the expression levels of the representative genes in each macrophage subtype.

Supplementary figure 6. Single-cell RNA-seq analysis reveals disease-specific macrophage subpopulations in FHP lungs. Related to Figure 2. (A) The bubble plot showing GO analysis of DEGs in each macrophage subtype. (B) AUCell analyses of the relative gene set enrichment scores in Para-inflammation signature. The t-SNE map showing AUC scores of selected gene signatures (left); the box plot showing AUC scores of selected gene signatures in each cell type (middle) or sample group (right). (C)Heatmap for enrichment scores of hallmark gene sets in each macrophage subtype. (D) The bar plot showing GSVA scores of hallmark gene sets between FHP-specific macrophages and other macrophages.

Supplementary figure 7. Single-cell RNA-seq analysis reveals disease-specific macrophage subpopulations in FHP lungs. Related to Figure 2. (A) The volcano plot showing DEGs that are upregulated (red) or downregulated (blue) in

FHP-specific macrophages. (B) Violin plots displaying the expression levels of the representative genes in each macrophage subcluster. (C) The bubble plot showing expression levels of cytokine and chemokine-related genes between FHP group and control group. (D) The bar plot showing GO analysis of upregulated DEGs in (A).

Supplementary figure 8. Single-cell RNA-seq analysis reveals disease-specific macrophage subpopulations in FHP lungs. Related to Figure 2. (A) Expression levels of PLA2G7 are projected onto the t-SNE map. (B) The volcano plot showing DEGs that are upregulated (red) or downregulated (blue) in PLA2G7^{high} macrophages. (C) The bar plot showing GO analysis of upregulated DEGs in (B).

Supplementary figure 9. Single-cell RNA-seq analysis reveals disease-specific macrophage subpopulations in FHP lungs. Related to Figure 2. (A) The t-SNE map showing the distribution of CCL4^{high} and CCL4^{low} macrophages. (B) The t-SNE maps showing the distribution of CCL4^{high} and CCL4^{low} macrophages across sample origins. (C) Multiplex immunofluorescence images of CD68 and CCL4 in lung tissues from the patient with FHP. Scale bar=10 μm. White arrow, CD68^{+} CCL4^{+} cells.

Supplementary figure 10. Single-cell RNA-seq analysis reveals disease-specific macrophage subpopulations in FHP lungs. Related to Figure 2. (A-B) Developmental trajectory of macrophages inferred by Monocle2. Cells are colored based on pseudotime states (A) or cell subclusters (B). (C-D) Lollipop plots showing the expression levels of the representative genes in each sample (upper) or macrophage subtype (lower) based on pseudotime trajectory.

Supplementary figure 11. Single-cell RNA-seq analysis reveals disease-specific macrophage subpopulations in FHP lungs. Related to Figure 2. (A) AUC value heatmap for the regulon activity of transcription factor motifs in each subtype. (B) The t-SNE map of macrophage subtype distribution of Figure 2K. (C) t-SNE visualization of AUC values of STAT1 motif in macrophages.

Supplementary figure 12. Single-Cell transcriptional analysis reveals heterogeneity of alveolar epithelial cells in FHP patients. Related to Figure 3. (A) t-SNE visualization of 13 alveolar epithelial cell subclusters. (B) The expression levels of top 3 marker genes in cell subclusters. (C) Average cell numbers (left) and relative percentage (right) of cell subtypes across samples. (D) Violin plots displaying the expression levels of the representative genes in each cell subtype (upper) or each sample (lower).

Supplementary figure 13. Single-Cell transcriptional analysis reveals heterogeneity of alveolar epithelial cells in FHP patients. Related to Figure 3. (A) Heatmap for enrichment scores of hallmark gene sets in each alveolar epithelial cell subtype. (B) AUCell analyses of the relative gene set enrichment scores in Lung Basal Cell signature (upper) and EMT signature (lower).

Supplementary figure 14. Single-Cell transcriptional analysis reveals heterogeneity of alveolar epithelial cells in FHP patients. Related to Figure 3. (A) Expression levels of KRT17 are projected onto the t-SNE map. (B) The volcano plot showing DEGs that are upregulated (red) or downregulated (blue) in KRT17^{high} cells. (C) The bar plot showing GO analysis of upregulated DEGs in (B).

Supplementary figure 15. Single-Cell transcriptional analysis reveals heterogeneity of alveolar epithelial cells in FHP patients. Related to Figure 3. Spliced-unspliced phase portraits of KRT17.

Supplementary figure 16. Single-Cell transcriptional analysis reveals heterogeneity of alveolar epithelial cells in FHP patients. Related to Figure 3. (A) The AUCell score distribution for the SOX4_extended motif. (B) t-SNE visualization of AUC values of SOX4_extended motif in alveolar epithelial cells.

Supplementary figure 17. Single-Cell transcriptional analysis unravels heterogeneity of fibroblasts during FHP. Related to Figure 4. (A) t-SNE visualization of 4 fibroblast subclusters. (B) The bubble plot showing GO analysis of DEGs in each fibroblast subtype. (C) Heatmap for enrichment scores of hallmark gene sets in each fibroblast subtype.

Supplementary figure 18. Single-Cell transcriptional analysis unravels heterogeneity of fibroblasts during FHP. Related to Figure 4. Dot plots showing the expression pattern of the representative genes in each fibroblast subtype (A) based on pseudotime trajectory (B).

Supplementary figure 19. Single-Cell transcriptional analysis unravels heterogeneity of fibroblasts during FHP. Related to Figure 4. AUC value heatmap for the regulon activity of transcription factor motifs in each fibroblast subtype.

Supplementary figure 20. Single-Cell transcriptional analysis unveils functional heterogeneity of T cells and NK cells in patients with FHP. Related to Figure 5. (A) t-SNE visualization of 14 subclusters. (B) The expression levels of top 3 marker genes in cell subtypes. (C) The bubble plot showing GO analysis of DEGs in each cell subtype. (D) Heatmap for enrichment scores of Immport immune-related gene signatures in each cell subtypes. (E-H) AUCell analyses of the relative gene set enrichment scores in CD8+ T cell signature (E), HLA signature (F), MHC-1 signature (G), and Cytokines/Chemokines/Receptors signature (H). (I) AUC value heatmap for the regulon activity of transcription factor motifs in each cell subtype.

Supplementary figure 21. Integrated analysis in scRNA and bulk RNA-seq data. Related to Figure 6. Heatmap for expression levels of overlapping DEGs identified in Figure 6F (A) or Figure 6H (B).

Supplementary table legends

Supplementary table1-M1 and M2 signatures.

Supplementary table2-gene signatures.
