# Supplementary material for: Characterizing cellular heterogeneity in fibrotic hypersensitivity pneumonitis by single-cell transcriptional analysis
Source: Cell Death Discov. 2022 Jan 28;8:38. doi: 10.1038/s41420-022-00831-x (PMC8795750; doi:10.1038/s41420-022-00831-x)
Supplement: Supplementary file 25 — Supplementary figures 1-21 [file 41420_2022_831_MOESM25_ESM.pdf]

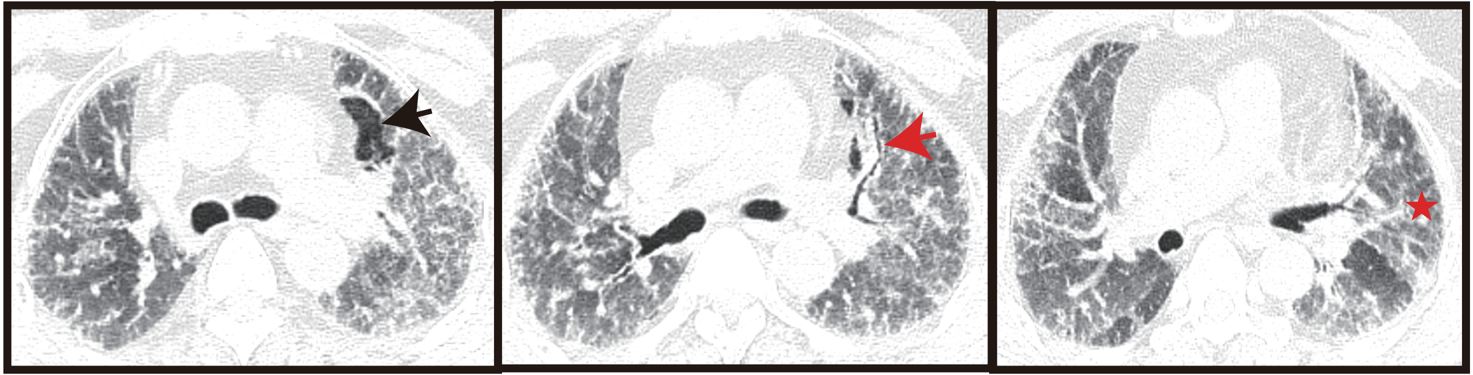

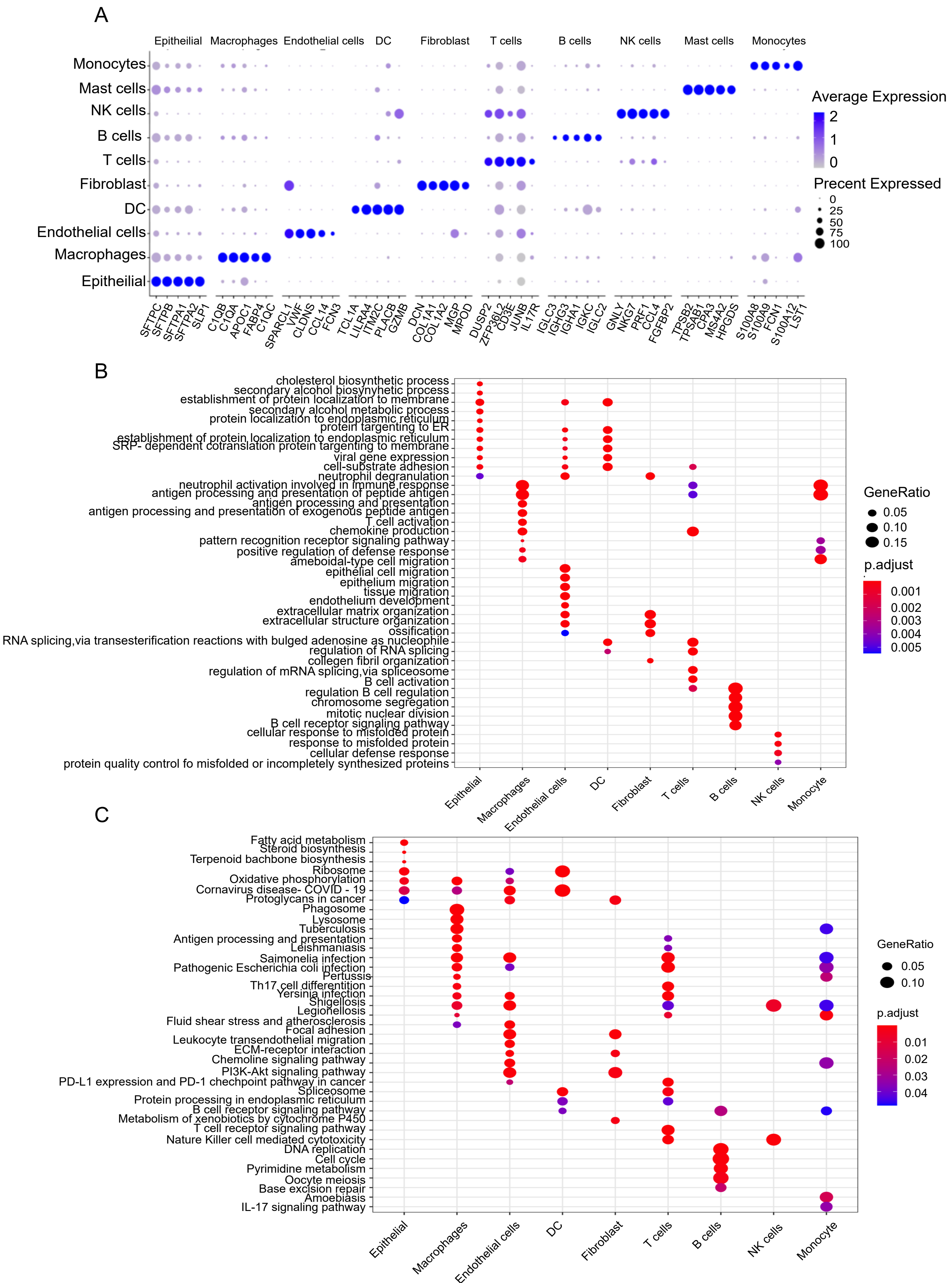
A
Epitheilial
Macrophages
Endothelial cells
DC
Fibroblast
T cells
B cells
NK cells
Mast cells
Monocytes
Monocytes
Mast cells
NK cells
B cells
T cells
Fibroblast
DC
Endothelial cells
Macrophages
Epitheilial
Average Expression
2
1
0
Precent Expressed
0
25
50
75
100
SFTPC
SFTPB
SFTPA1
SFTPA2
SLP1
C1QB
C1QA
APOC1
FABP4
C1QC
SPARCL1
VWF
CLDNS
CCL14
FCN3
TCL1A
LILRA4
ITM2C
PLACB
GZMB
DCN
COL1A1
COL1A2
MGP
MPOD
DUSP2
ZFP36L2
CD3E
JUNB
IL17R
IGLC3
IGHG3
IGHA1
IGKC
IGLC2
GNLY
NKG7
PRF1
CCL4
FGFBP2
TPSB2
TPSAB1
CPA3
MS4A2
HPGDS
S100A8
S100A9
FCN1
S100A12
LST1
B
cholesterol biosynthetic process
secondary alcohol biosynyhetic process
establishment of protein localization to membrane
secondary alcohol metabolic process
protein localization to endoplasmic reticulum
protein targenting to ER
establishment of protein localization to endoplasmic reticulum
SRP- dependent cotranslation protein targenting to membrane
viral gene expression
cell-substrate adhesion
neutrophil degranulation
neutrophil activation involved in immune response
antigen processing and presentation of peptide antigen
antigen processing and presentation
antigen processing and presentation of exogenous peptide antigen
T cell activation
chemokine production
pattern recognition receptor signaling pathway
positive regulation of defense response
ameboidal-type cell migration
epithelial cell migration
epithelium migration
tissue migration
endothelium development
extracellular matrix organization
extracellular structure organization
ossification
RNA splicing,via transesterification reactions with bulged adenosine as nucleophile
regulation of RNA splicing
collegen fibril organization
regulation of mRNA splicing,via spliceosome
B cell activation
regulation B cell regulation
chromosome segregation
mitotic nuclear division
B cell receptor signaling pathway
cellular response to misfolded protein
response to misfolded protein
cellular defense response
protein quality control fo misfolded or incompletely synthesized proteins
Epithelial
Macrophages
Endothelial cells
DC
Fibroblast
T cells
B cells
NK cells
Monocyte
GeneRatio
0.05
0.10
0.15
p.adjust
0.001
0.002
0.003
0.004
0.005
C
Fatty acid metabolism
Steroid biosynthesis
Terpenoid bachbone biosynthesis
Ribosome
Oxidative phosphorylation
Cornavirus disease- COVID - 19
Protoglycans in cancer
Phagosome
Lysosome
Tuberculosis
Antigen processing and presentation
Leishmaniasis
Saimonelia infection
Pathogenic Escherichia coli infection
Pertussis
Th17 cell differentition
Yersinia infection
Shigellosis
Legionellosis
Fluid shear stress and atherosclerosis
Focal adhesion
Leukocyte transendothelial migration
ECM-receptor interaction
Chemoline signaling pathway
PI3K-Akt signaling pathway
PD-L1 expression and PD-1 chechpoint pathway in cancer
Spliceosome
Protein processing in endoplasmic reticulum
B cell receptor signaling pathway
Metabolism of xenobiotics by cytochrome P450
T cell receptor signaling pathway
Nature Killer cell mediated cytotoxicity
DNA replication
Cell cycle
Pyrimidine metabolism
Oocyte meiosis
Base excision repair
Amoebiasis
IL-17 signaling pathway
Epithelial
Macrophages
Endothelial cells
DC
Fibroblast
T cells
B cells
NK cells
Monocyte
GeneRatio
0.05
0.10
p.adjust
0.01
0.02
0.03
0.04

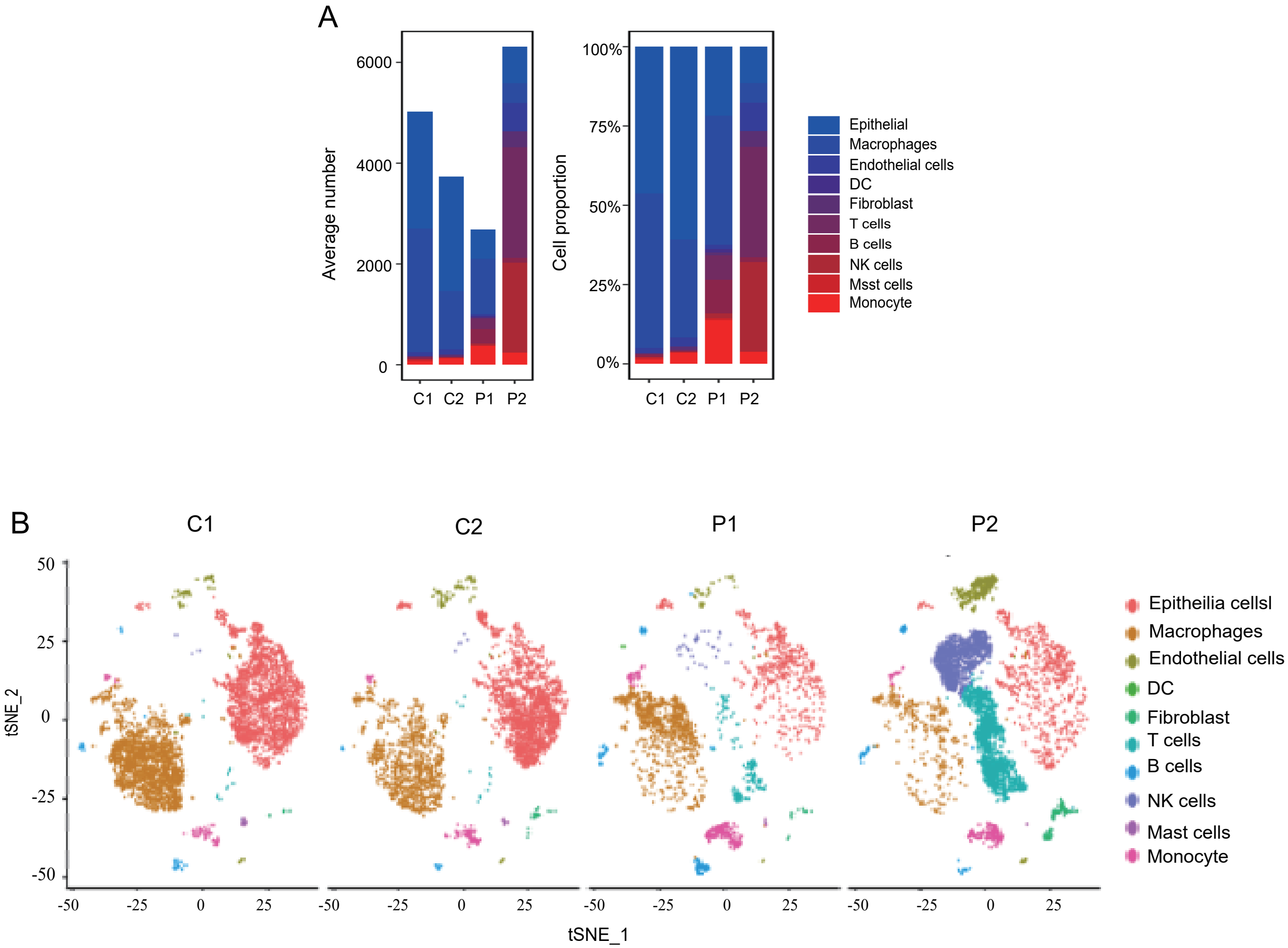

A
Average number
6000
4000
2000
0
C1
C2
P1
P2
Cell proportion
100%
75%
50%
25%
0%
Epithelial
Macrophages
Endothelial cells
DC
Fibroblast
T cells
B cells
NK cells
Msst cells
Monocyte
B
tSNE_2
tSNE_1
50
25
0
-25
-50
Epitheilia cellsl
Mast cells

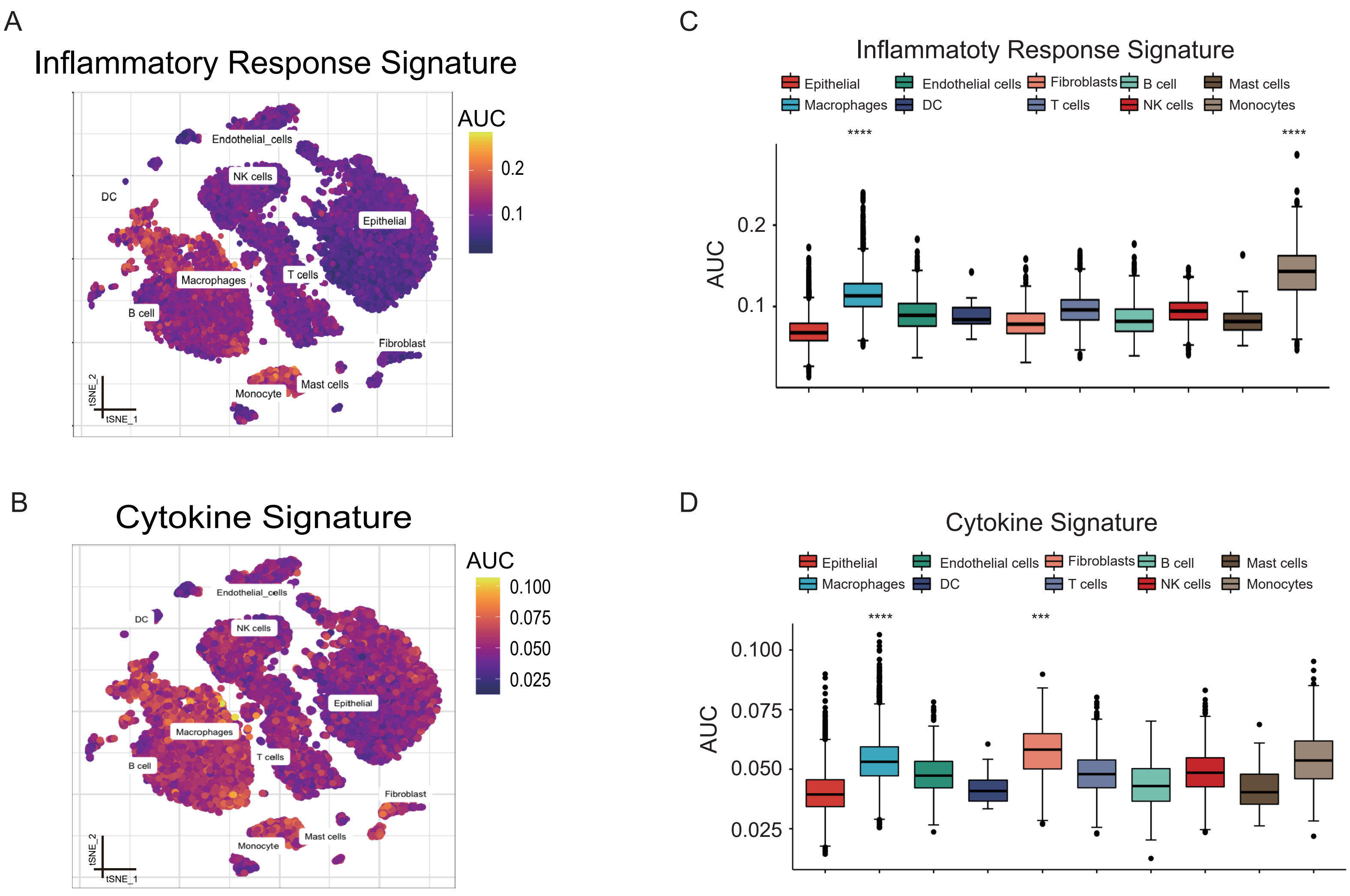
A
Inflammatory Response Signature
AUC
0.2
0.1
Endothelial_cells
NK cells
DC
Epithelial
Macrophages
T cells
B cell
Fibroblast
Mast cells
Monocyte
tSNE_2
tSNE_1
B
Cytokine Signature
AUC
0.100
0.075
0.050
0.025
Endothelial_cells
DC
NK cells
Epithelial
Macrophages
T cells
B cell
Fibroblast
Mast cells
Monocyte
tSNE_2
tSNE_1
C
Inflammatoty Response Signature
Epithelial
Endothelial cells
Fibroblasts
B cell
Mast cells
Macrophages
DC
T cells
NK cells
Monocytes
****
****
AUC
0.2
0.1
D
Cytokine Signature
Epithelial
Endothelial cells
Fibroblasts
B cell
Mast cells
Macrophages
DC
T cells
NK cells
Monocytes
****
***
AUC
0.100
0.075
0.050
0.025

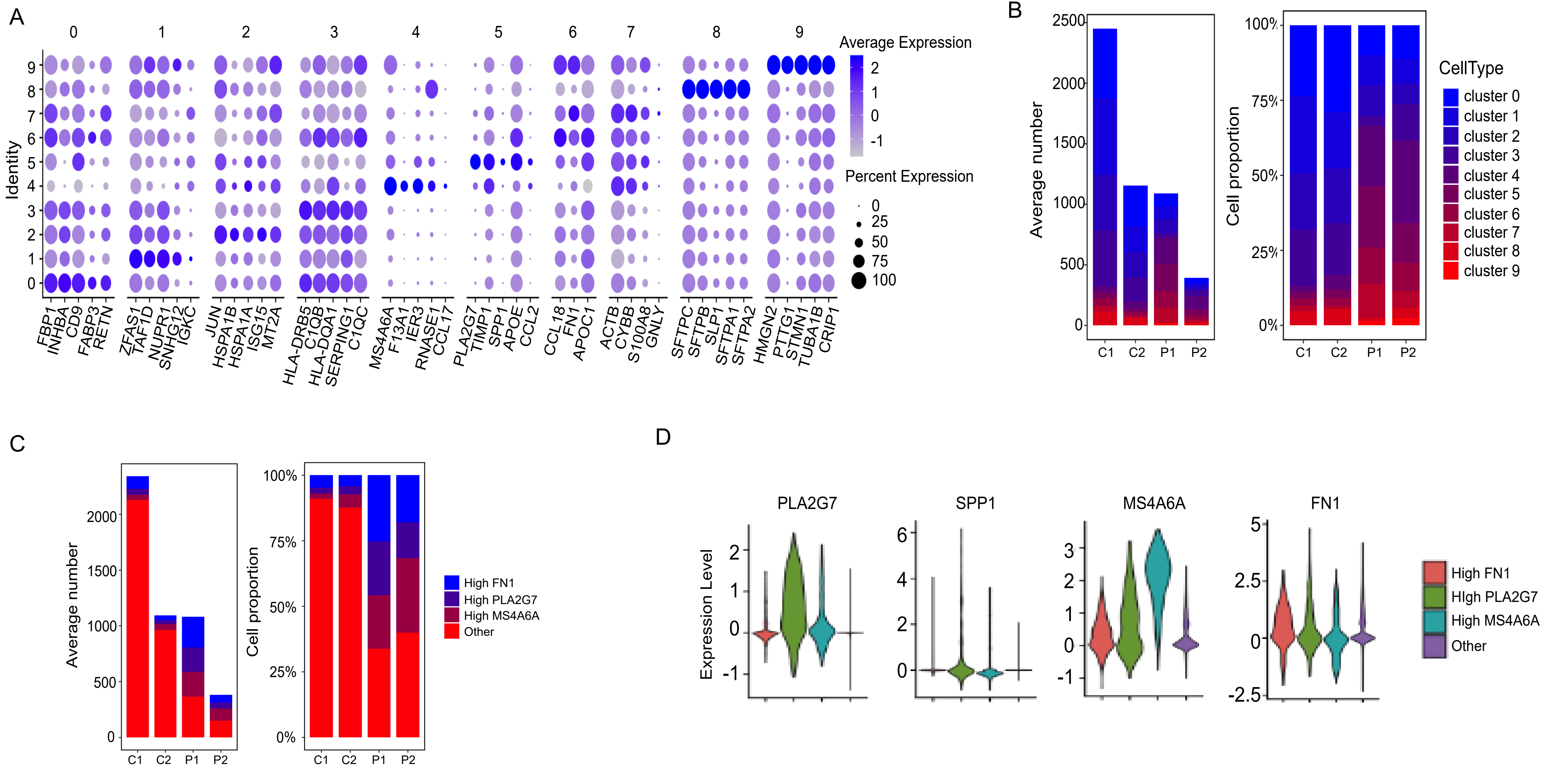

A
0
1
2
3
4
5
6
7
8
9
Identity
Average Expression
2
1
0
-1
Percent Expression
0
25
50
75
100
FBP1
INHBA
CD9
FABP3
RETN
ZFAS1
TAF1D
NUPR1
SNHG12
IGKC
JUN
HSPA1B
HSPA1A
ISG15
MT2A
HLA-DRB5
C1QB
HLA-DQA1
SERPING1
C1QC
MS4A6A
F13A1
IER3
RNASE1
CCL17
PLA2G7
TIMP1
SPP1
APOE
CCL2
CCL18
FN1
APOC1
ACTB
CYBB
S100A8
GNLY
SFTPC
SFTPB
SLP1
SFTPA1
SFTPA2
HMGN2
PTTG1
STMN1
TUBA1B
CRIP1
B
Average number
2500
2000
1500
1000
500
0
C1
C2
P1
P2
Cell proportion
100%
75%
50%
25%
0%
CellType
cluster 0
cluster 1
cluster 2
cluster 3
cluster 4
cluster 5
cluster 6
cluster 7
cluster 8
cluster 9
C
Average number
2000
1500
1000
500
0
Cell proportion
100%
75%
50%
25%
0%
High FN1
High PLA2G7
High MS4A6A
Other
D
PLA2G7
SPP1
MS4A6A
FN1
Expression Level
High FN1
HIgh PLA2G7
High MS4A6A
Other

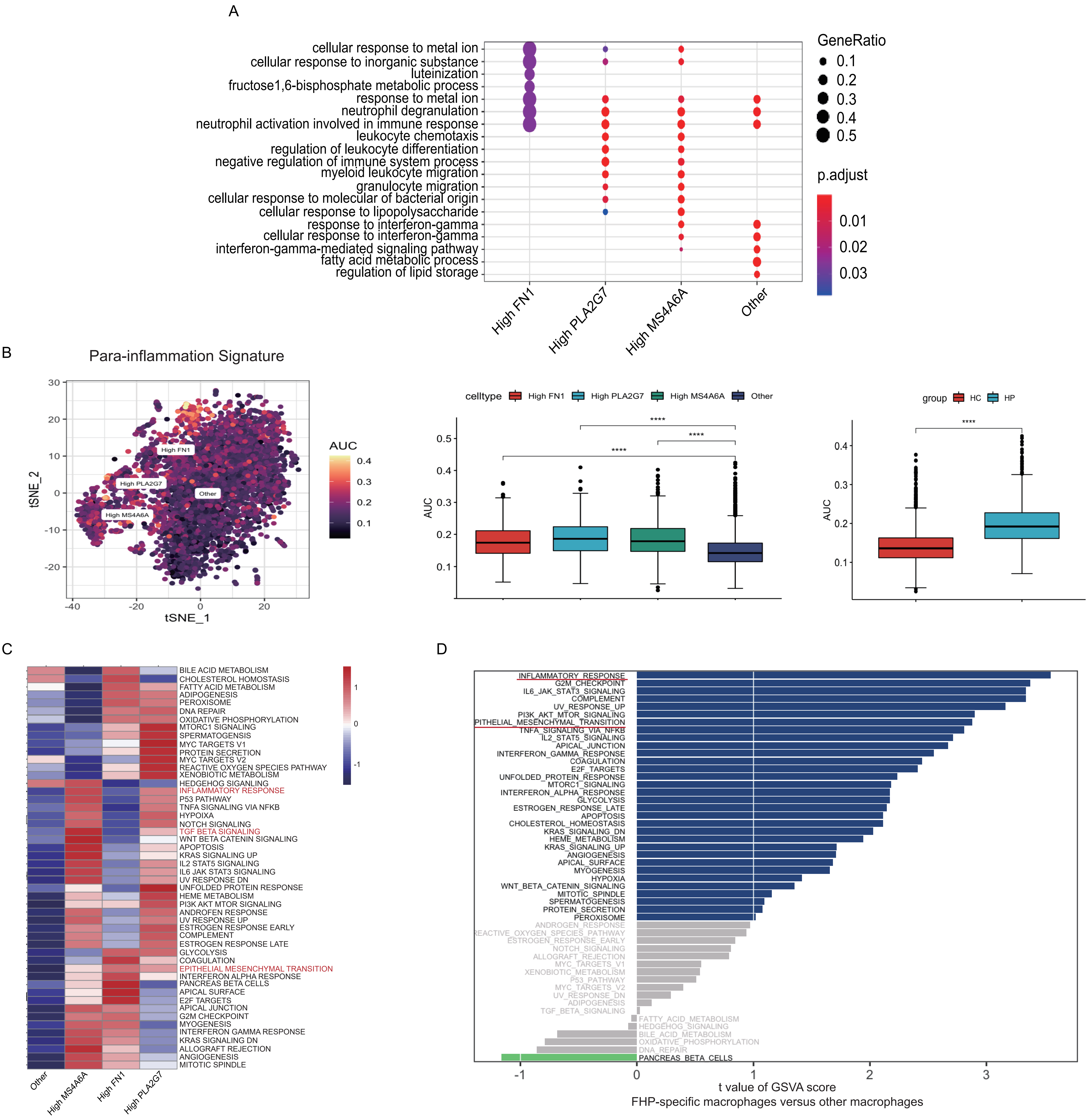
A
cellular response to metal ion
cellular response to inorganic substance
luteinization
fructose1,6-bisphosphate metabolic process
response to metal ion
neutrophil degranulation
neutrophil activation involved in immune response
leukocyte chemotaxis
regulation of leukocyte differentiation
negative regulation of immune system process
myeloid leukocyte migration
granulocyte migration
cellular response to molecular of bacterial origin
cellular response to lipopolysaccharide
response to interferon-gamma
cellular response to interferon-gamma
interferon-gamma-mediated signaling pathway
fatty acid metabolic process
regulation of lipid storage
High FN1
High PLA2G7
High MS4A6A
Other
GeneRatio
0.1
0.2
0.3
0.4
0.5
p.adjust
0.01
0.02
0.03
B
Para-inflammation Signature
tSNE_2
tSNE_1
High FN1
High PLA2G7
High MS4A6A
Other
AUC
0.4
0.3
0.2
0.1
celltype
High FN1
High PLA2G7
High MS4A6A
Other
****
AUC
group
HC
HP
C
BILE ACID METABOLISM
CHOLESTEROL HOMOSTASIS
FATTY ACID METABOLISM
ADIPOGENESIS
PEROXISOME
DNA REPAIR
OXIDATIVE PHOSPHORYLATION
MTORC1 SIGNALING
SPERMATOGENSIS
MYC TARGETS V1
PROTEIN SECRETION
MYC TARGETS V2
REACTIVE OXYGEN SPECIES PATHWAY
XENOBIOTIC METABOLISM
HEDGEHOG SIGANLING
INFLAMMATORY RESPONSE
P53 PATHWAY
TNFA SIGNALING VIA NFKB
HYPOIXA
NOTCH SIGNALING
TGF BETA SIGNALING
WNT BETA CATENIN SIGNALING
APOPTOSIS
KRAS SIGNALING UP
IL2 STAT5 SIGNALING
IL6 JAK STAT3 SIGNALING
UV RESPONSE DN
UNFOLDED PROTEIN RESPONSE
HEME METABOLISM
PI3K AKT MTOR SIGNALING
ANDROFEN RESPONSE
UV RESPONSE UP
ESTROGEN RESPONSE EARLY
COMPLEMENT
ESTROGEN RESPONSE LATE
GLYCOLYSIS
COAGULATION
EPITHELIAL MESENCHYMAL TRANSITION
INTERFERON ALPHA RESPONSE
PANCREAS BETA CELLS
APICAL SURFACE
E2F TARGETS
APICAL JUNCTION
G2M CHECKPOINT
MYOGENESIS
INTERFERON GAMMA RESPONSE
KRAS SIGNALING DN
ALLOGRAFT REJECTION
ANGIOGENESIS
MITOTIC SPINDLE
Other
High MS4A6A
High FN1
High PLA2G7
D
INFLAMMATORY_RESPONSE
G2M_CHECKPOINT
IL6_JAK_STAT3_SIGNALING
COMPLEMENT
UV_RESPONSE_UP
PI3K_AKT_MTOR_SIGNALING
PITHELIAL_MESENCHYMAL_TRANSITION
TNFA_SIGNALING_VIA_NFKB
IL2_STAT5_SIGNALING
APICAL_JUNCTION
INTERFERON_GAMMA_RESPONSE
COAGULATION
E2F_TARGETS
UNFOLDED_PROTEIN_RESPONSE
MTORC1_SIGNALING
INTERFERON_ALPHA_RESPONSE
GLYCOLYSIS
ESTROGEN_RESPONSE_LATE
APOPTOSIS
CHOLESTEROL_HOMEOSTASIS
KRAS_SIGNALING_DN
HEME_METABOLISM
KRAS_SIGNALING_UP
ANGIOGENESIS
APICAL_SURFACE
MYOGENESIS
HYPOXIA
WNT_BETA_CATENIN_SIGNALING
MITOTIC_SPINDLE
SPERMATOGENESIS
PROTEIN_SECRETION
PEROXISOME
ANDROGEN_RESPONSE
REACTIVE_OXYGEN_SPECIES_PATHWAY
ESTROGEN_RESPONSE_EARLY
NOTCH_SIGNALING
ALLOGRAFT_REJECTION
MYC_TARGETS_V1
XENOBIOTIC_METABOLISM
P53_PATHWAY
MYC_TARGETS_V2
UV_RESPONSE_DN
ADIPOGENESIS
TGF_BETA_SIGNALING
FATTY_ACID_METABOLISM
HEDGEHOG_SIGNALING
BILE_ACID_METABOLISM
OXIDATIVE_PHOSPHORYLATION
DNA_REPAIR
PANCREAS_BETA_CELLS
t value of GSVA score
FHP-specific macrophages versus other macrophages

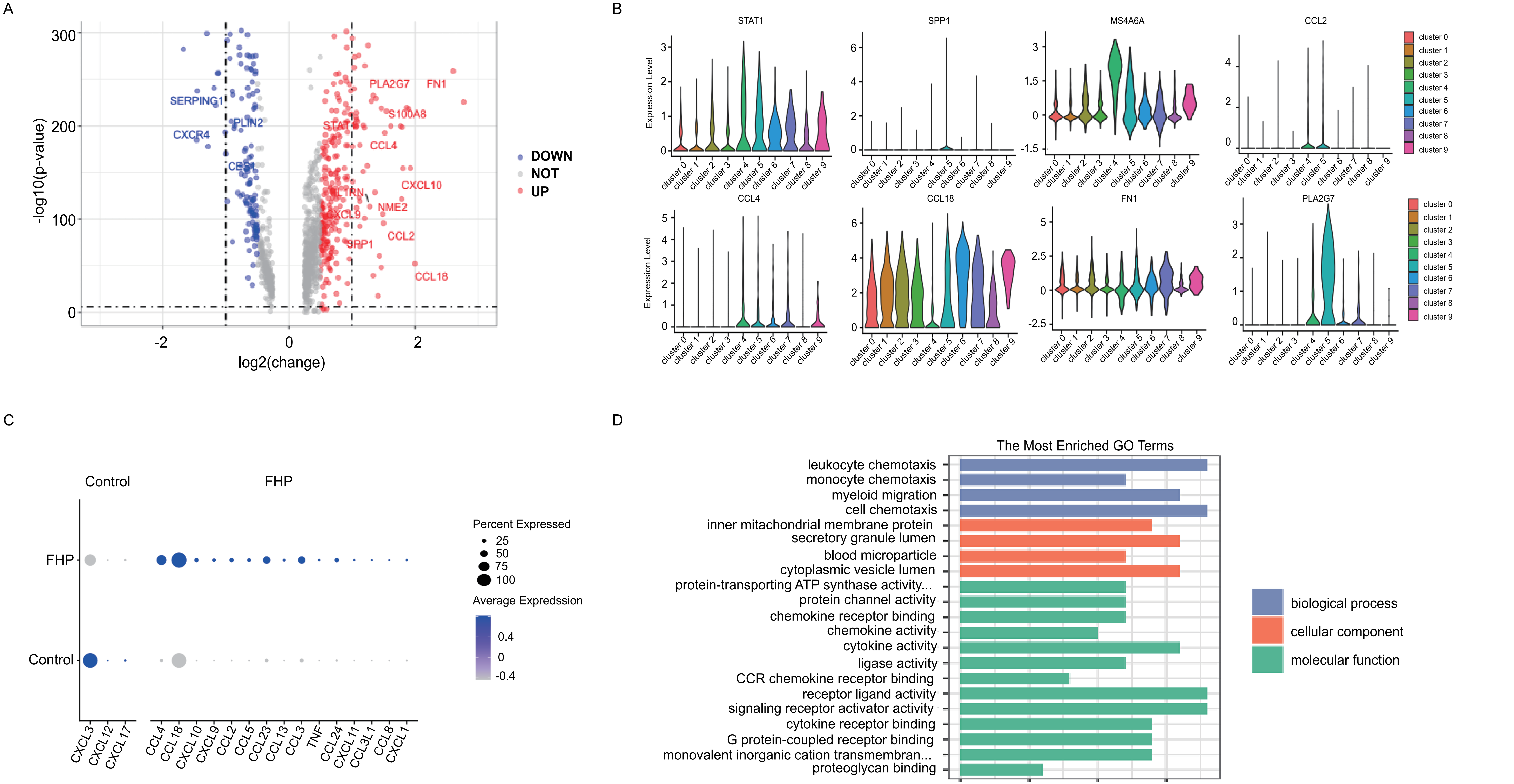

A
-log10(p-value)
log2(change)
DOWN
NOT
UP
SERPING1
CXCR4
PLIN2
PLA2G7
FN1
S100A8
CCL4
CXCL10
NME2
CCL2
SPP1
CCL18
B
STAT1
SPP1
MS4A6A
CCL2
CCL4
CCL18
FN1
PLA2G7
Expression Level
cluster 0
cluster 1
cluster 2
cluster 3
cluster 4
cluster 5
cluster 6
cluster 7
cluster 8
cluster 9
C
Control
FHP
Percent Expressed
25
50
75
100
Average Expredssion
0.4
0
-0.4
CXCL3
CXCL12
CXCL17
CCL4
CCL18
CXCL10
CXCL9
CCL2
CCL5
CCL23
CCL13
CCL3
TNF
CCL24
CXCL11
CCL3L1
CCL8
CXCL1
D
The Most Enriched GO Terms
leukocyte chemotaxis
monocyte chemotaxis
myeloid migration
cell chemotaxis
inner mitachondrial membrane protein
secretory granule lumen
blood microparticle
cytoplasmic vesicle lumen
protein-transporting ATP synthase activity...
protein channel activity
chemokine receptor binding
chemokine activity
cytokine activity
ligase activity
CCR chemokine receptor binding
receptor ligand activity
signaling receptor activator activity
cytokine receptor binding
G protein-coupled receptor binding
monovalent inorganic cation transmembran...
proteoglycan binding
0.0
2.5
5.0
7.5
biological process
cellular component
molecular function

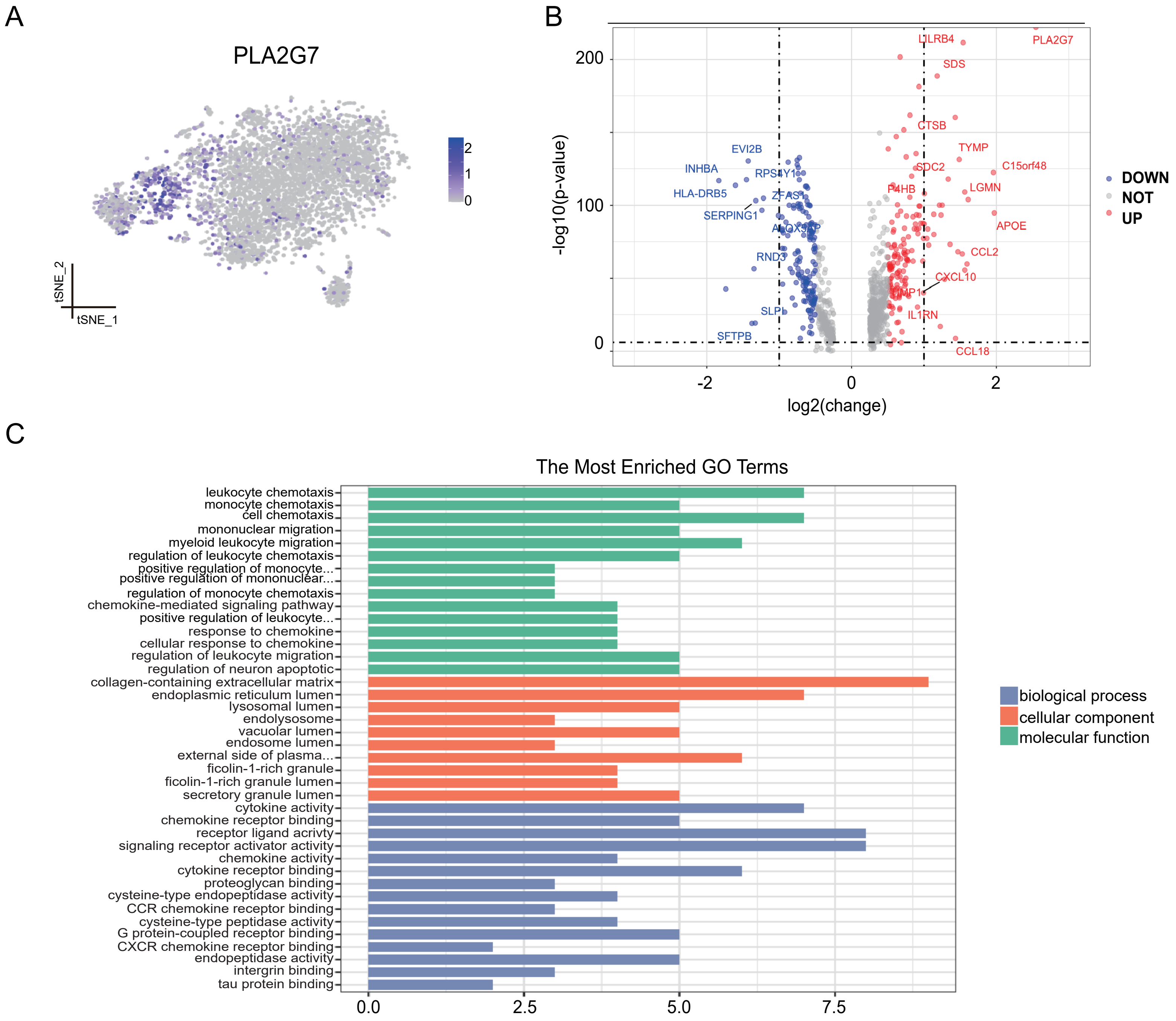

A
PLA2G7
2
1
0
tSNE_2
tSNE_1
B
200
100
0
-log10(p-value)
-2
0
2
log2(change)
LILRB4
PLA2G7
SDS
CTSB
TYMP
C15orf48
SDC2
LGMN
P4HB
APOE
CCL2
CXCL10
TIMP1
IL1RN
CCL18
EVI2B
INHBA
RPS4Y1
HLA-DRB5
ZFAS1
SERPING1
ALOX5AP
RND3
SLPI
SFTPB
DOWN
NOT
UP
C
The Most Enriched GO Terms
leukocyte chemotaxis
monocyte chemotaxis
cell chemotaxis
mononuclear migration
myeloid leukocyte migration
regulation of leukocyte chemotaxis
positive regulation of monocyte...
positive regulation of mononuclear...
regulation of monocyte chemotaxis
chemokine-mediated signaling pathway
positive regulation of leukocyte...
response to chemokine
cellular response to chemokine
regulation of leukocyte migration
regulation of neuron apoptotic
collagen-containing extracellular matrix
endoplasmic reticulum lumen
lysosomal lumen
endolysosome
vacuolar lumen
endosome lumen
external side of plasma...
ficolin-1-rich granule
ficolin-1-rich granule lumen
secretory granule lumen
cytokine activity
chemokine receptor binding
receptor ligand acrivty
signaling receptor activator activity
chemokine activity
cytokine receptor binding
proteoglycan binding
cysteine-type endopeptidase activity
CCR chemokine receptor binding
cysteine-type peptidase activity
G protein-coupled receptor binding
CXCR chemokine receptor binding
endopeptidase activity
intergrin binding
tau protein binding
0.0
2.5
5.0
7.5
biological process
cellular component
molecular function

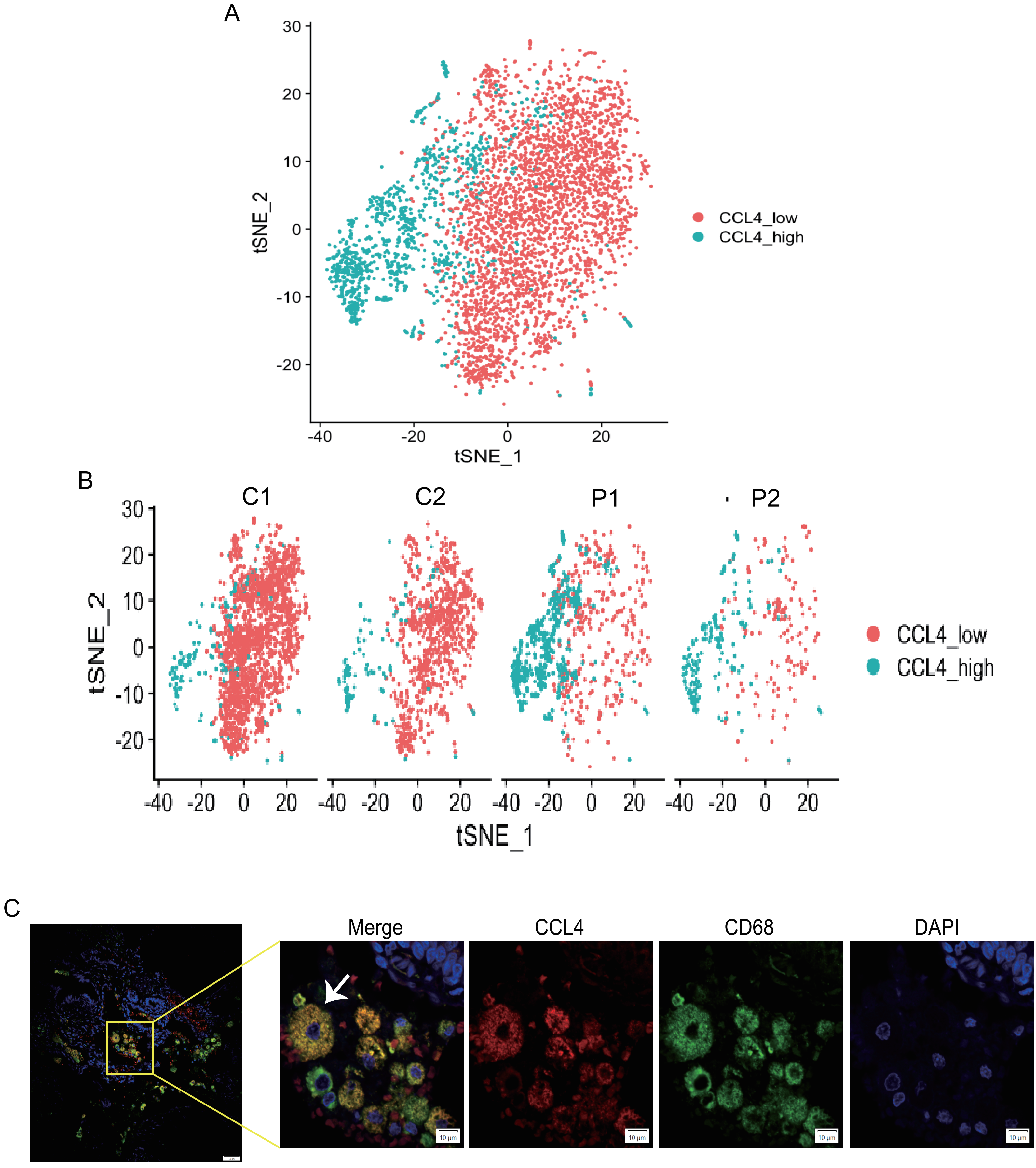
A
tSNE_2
tSNE_1
CCL4_low
CCL4_high
B
C1
C2
P1
P2
tSNE_2
tSNE_1
CCL4_low
CCL4_high
C
Merge
CCL4
CD68
DAPI
10 μm

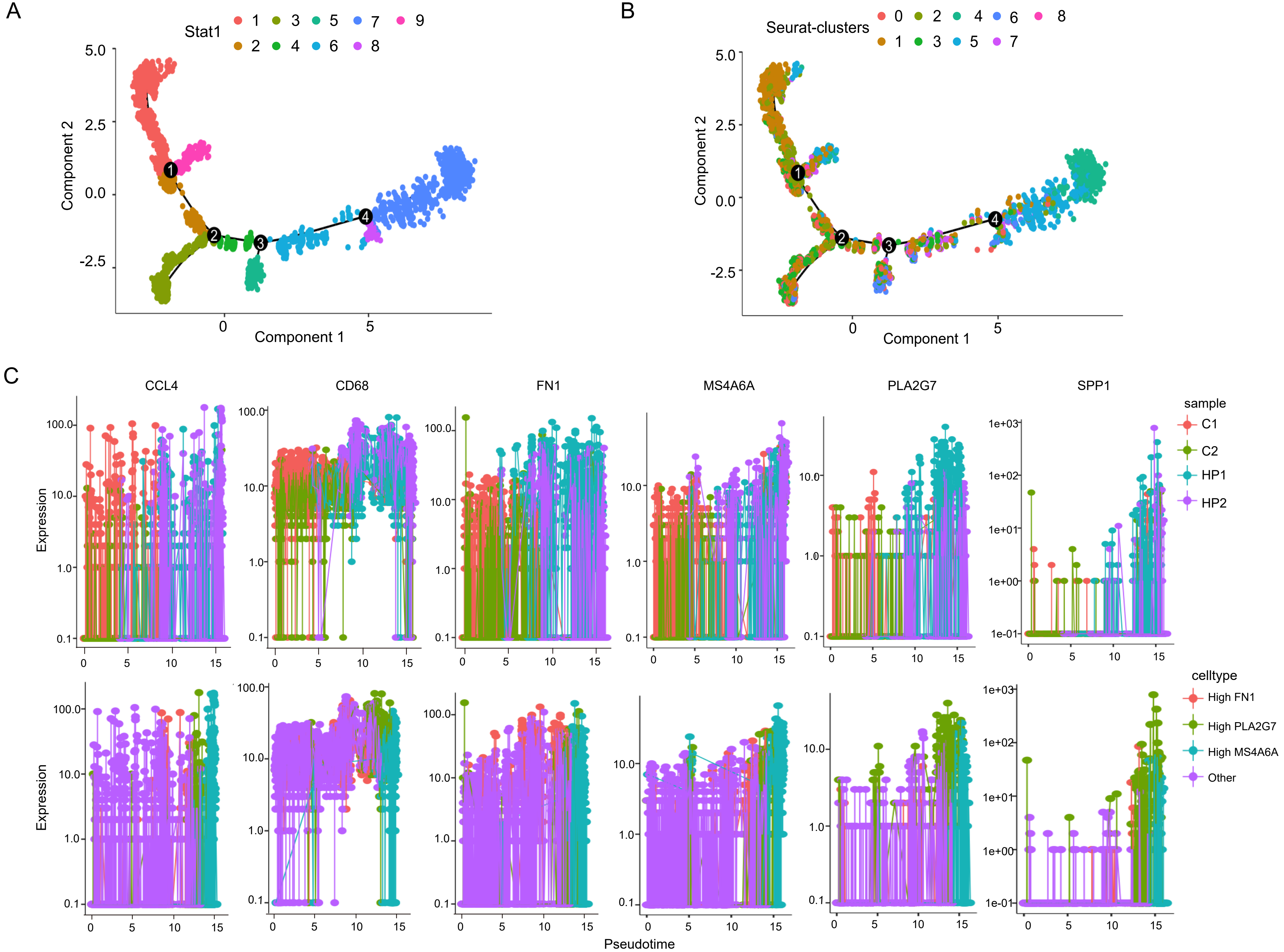

A
Stat1
1 2 3 4 5 6 7 8 9
Component 2
Component 1
B
Seurat-clusters
0 1 2 3 4 5 6 7 8
Component 2
Component 1
C
CCL4
CD68
FN1
MS4A6A
PLA2G7
SPP1
Expression
sample
C1
C2
HP1
HP2
celltype
High FN1
High PLA2G7
High MS4A6A
Other
Pseudotime

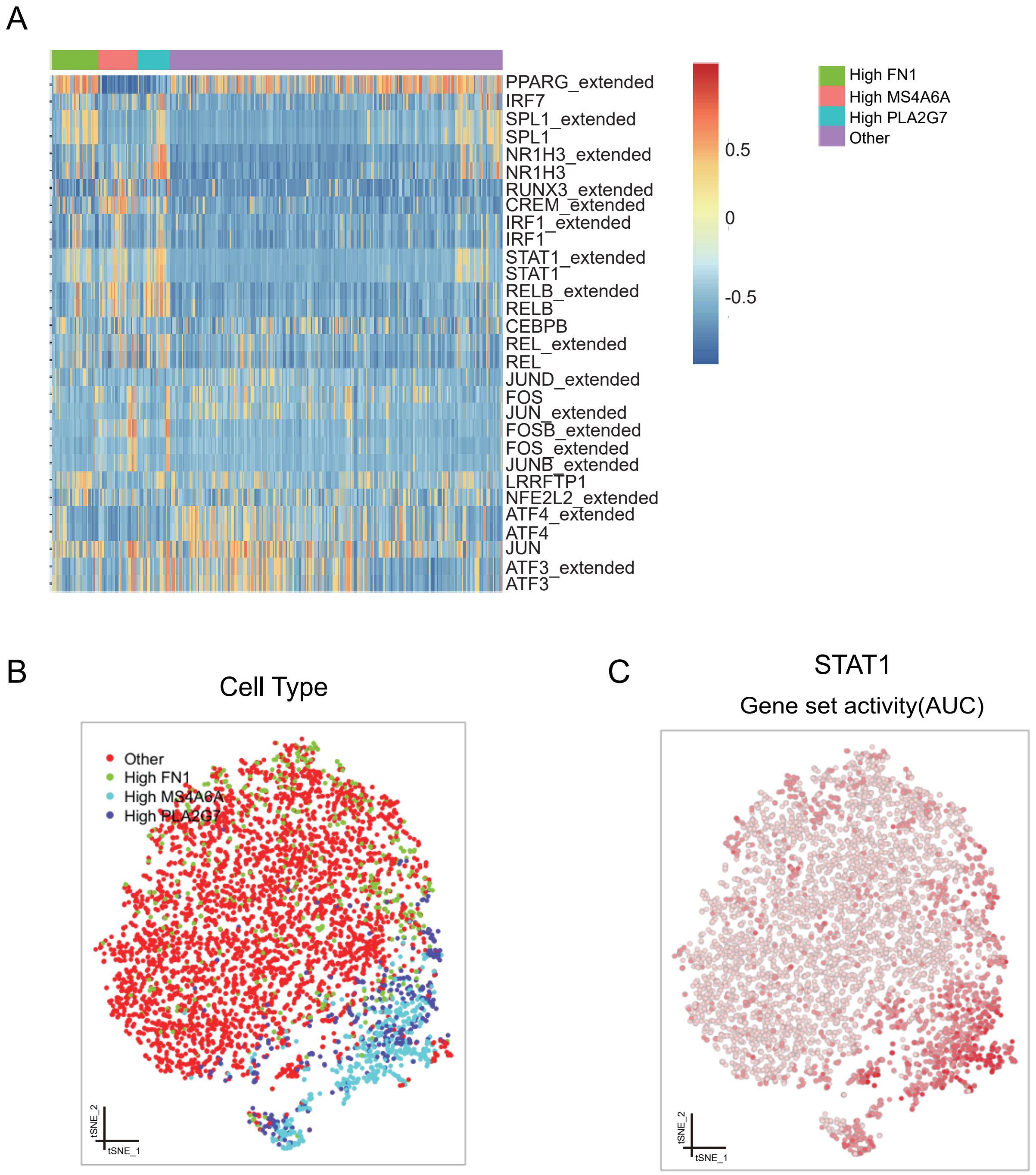
A
PPARG_extended
IRF7
SPL1_extended
SPL1
NR1H3_extended
NR1H3
RUNX3_extended
CREM_extended
IRF1_extended
IRF1
STAT1_extended
STAT1
RELB_extended
RELB
CEBPB
REL_extended
REL
JUND_extended
FOS
JUN_extended
FOSB_extended
FOS_extended
JUNB_extended
LRRFTP1
NFE2L2_extended
ATF4_extended
ATF4
JUN
ATF3_extended
ATF3
0.5
0
-0.5
High FN1
High MS4A6A
High PLA2G7
Other
B
Cell Type
Other
High FN1
High MS4A6A
High PLA2G7
tSNE_2
tSNE_1
C
STAT1
Gene set activity(AUC)
tSNE_2
tSNE_1

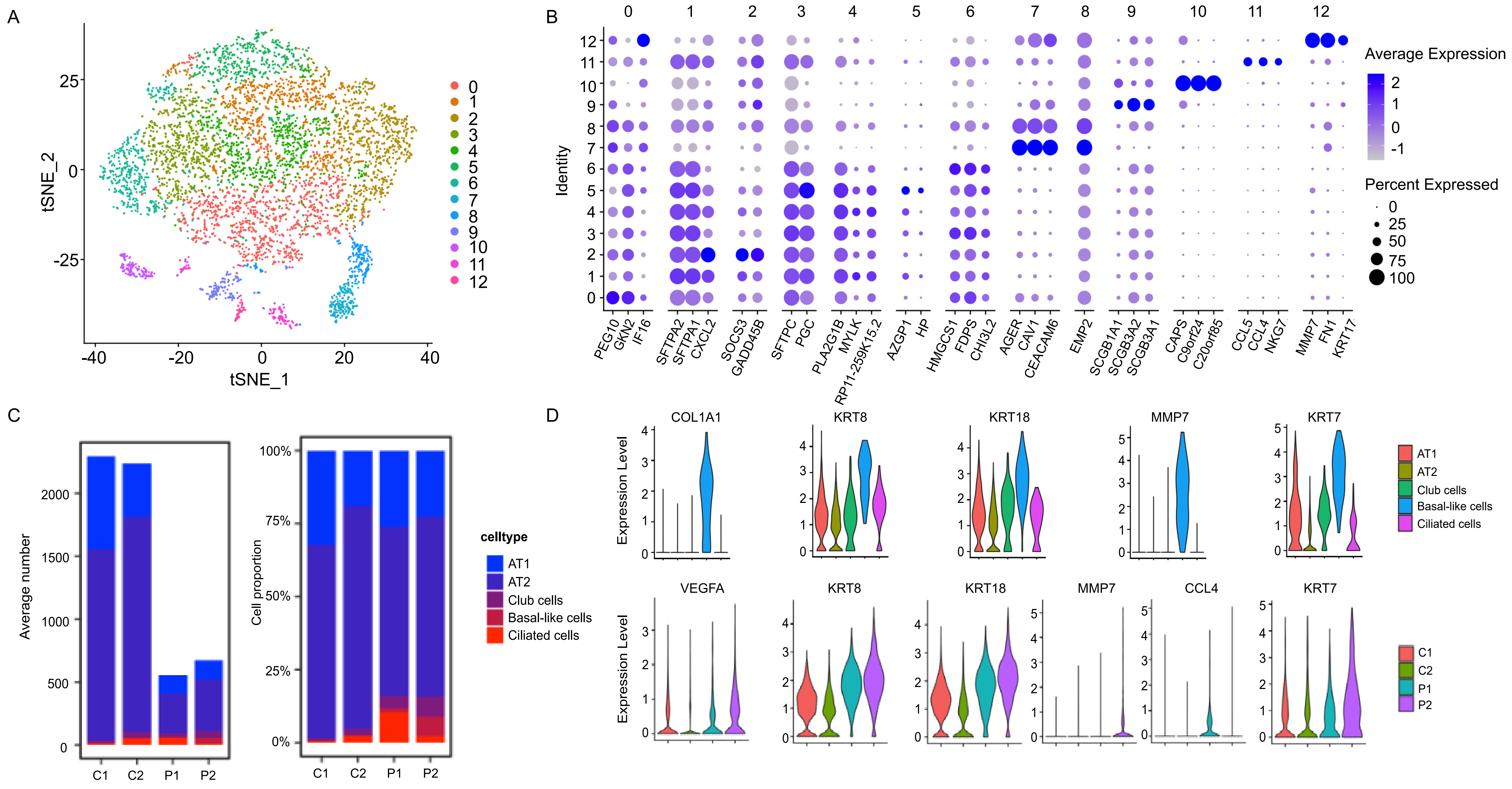

A
tSNE_1
tSNE_2
B
Identity
Average Expression
Percent Expressed
PEG10 GKN2 IF16 SFTPA2 SFTPA1 CXCL2 SOCS3 GADD45B SFTPC PGC PLA2G1B MYLK RP11-259K15.2 AZGP1 HP HMGCS1 FDPS CHI3L2 AGER CAV1 CEACAM6 EMP2 SCGB1A1 SCGB3A2 SCGB3A1 CAPS C9orf24 C20orf85 CCL5 CCL4 NKG7 MMP7 FN1 KRT17
C
Average number
Cell proportion
celltype
AT1
AT2
Club cells
Basal-like cells
Ciliated cells
C1
C2
P1
P2
D
Expression Level
COL1A1
KRT8
KRT18
MMP7
KRT7
VEGFA
CCL4

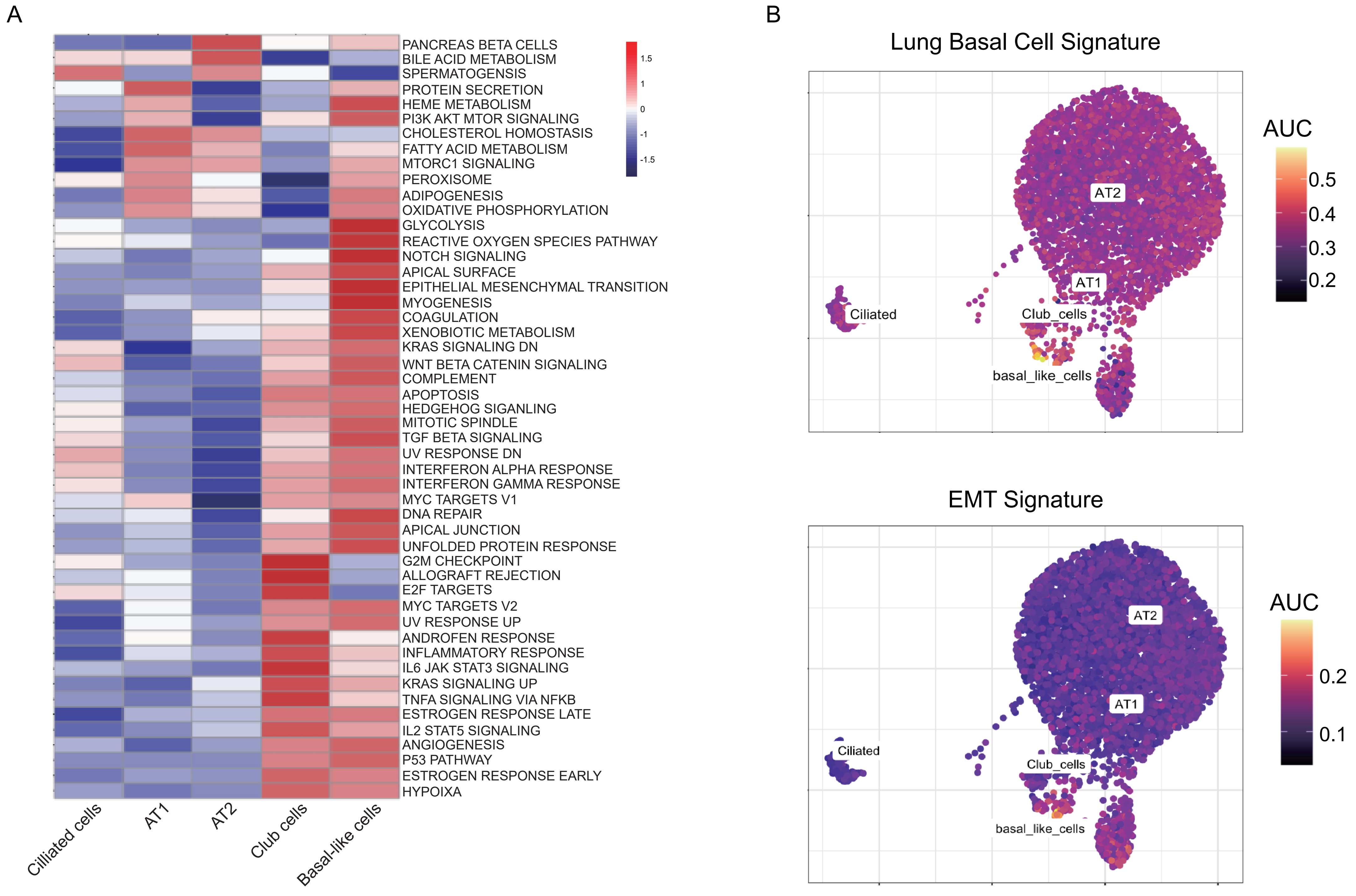
A
PANCREAS BETA CELLS
BILE ACID METABOLISM
SPERMATOGENSIS
PROTEIN SECRETION
HEME METABOLISM
PI3K AKT MTOR SIGNALING
CHOLESTEROL HOMOSTASIS
FATTY ACID METABOLISM
MTORC1 SIGNALING
PEROXISOME
ADIPOGENESIS
OXIDATIVE PHOSPHORYLATION
GLYCOLYSIS
REACTIVE OXYGEN SPECIES PATHWAY
NOTCH SIGNALING
APICAL SURFACE
EPITHELIAL MESENCHYMAL TRANSITION
MYOGENESIS
COAGULATION
XENOBIOTIC METABOLISM
KRAS SIGNALING DN
WNT BETA CATENIN SIGNALING
COMPLEMENT
APOPTOSIS
HEDGEHOG SIGANLING
MITOTIC SPINDLE
TGF BETA SIGNALING
UV RESPONSE DN
INTERFERON ALPHA RESPONSE
INTERFERON GAMMA RESPONSE
MYC TARGETS V1
DNA REPAIR
APICAL JUNCTION
UNFOLDED PROTEIN RESPONSE
G2M CHECKPOINT
ALLOGRAFT REJECTION
E2F TARGETS
MYC TARGETS V2
UV RESPONSE UP
ANDROFEN RESPONSE
INFLAMMATORY RESPONSE
IL6 JAK STAT3 SIGNALING
KRAS SIGNALING UP
TNFA SIGNALING VIA NFKB
ESTROGEN RESPONSE LATE
IL2 STAT5 SIGNALING
ANGIOGENESIS
P53 PATHWAY
ESTROGEN RESPONSE EARLY
HYPOIXA
1.5
1
0
-1
-1.5
Cilliated cells
AT1
AT2
Club cells
Basal-like cells
B
Lung Basal Cell Signature
AUC
0.5
0.4
0.3
0.2
AT2
AT1
Ciliated
Club_cells
basal_like_cells
EMT Signature
AUC
0.2
0.1
AT2
AT1
Ciliated
Club_cells
basal_like_cells

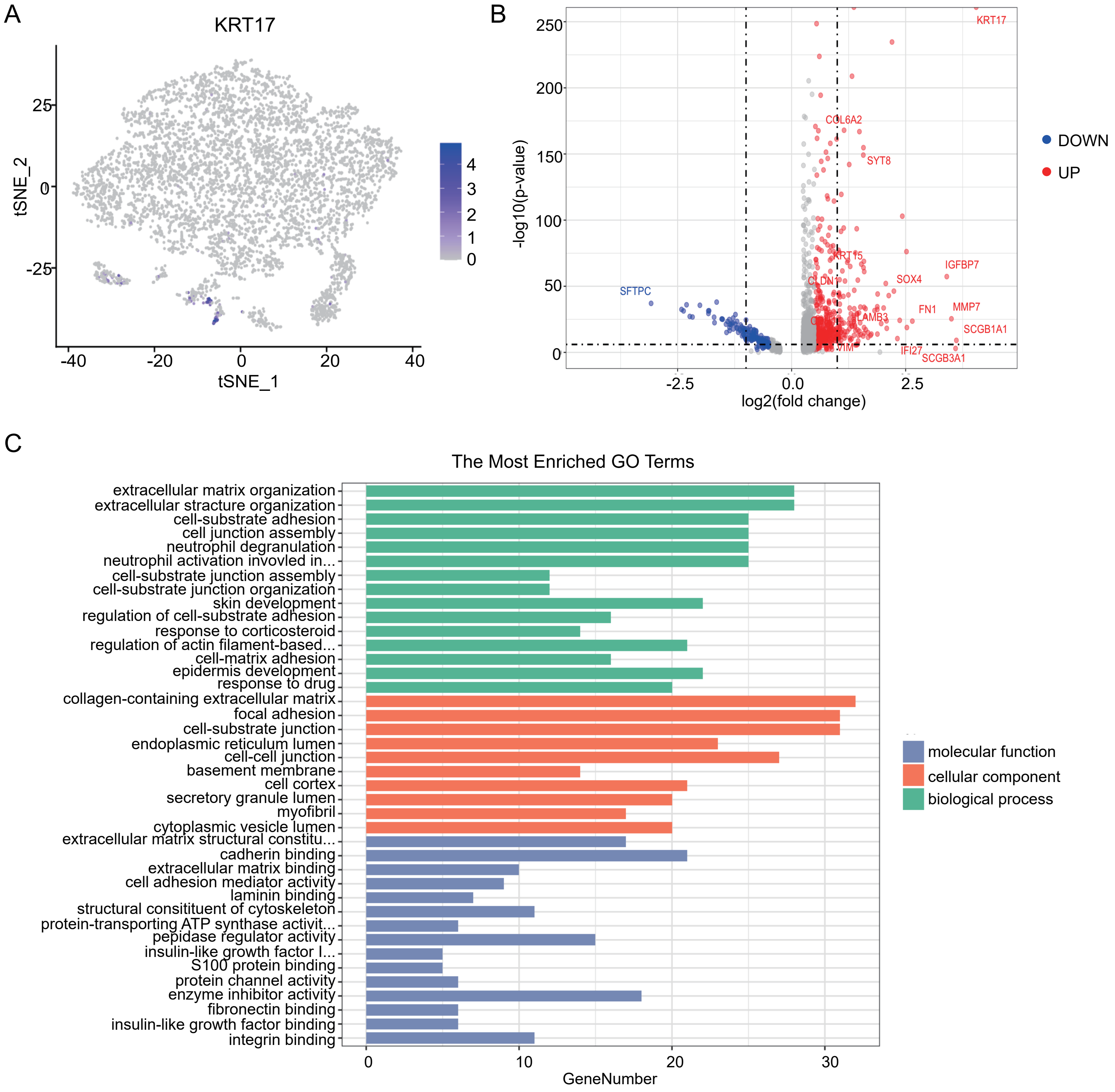

A
KRT17
tSNE_2
tSNE_1
B
-log10(p-value)
log2(fold change)
DOWN
UP
SFTPC
KRT17
COL6A2
SYT8
KRT15
CLDN1
IGFBP7
SOX4
FN1
MMP7
LAMB3
SCGB1A1
VIM
IFI27
SCGB3A1
C
The Most Enriched GO Terms
extracellular matrix organization
extracellular structure organization
cell-substrate adhesion
cell junction assembly
neutrophil degranulation
neutrophil activation invovled in...
cell-substrate junction assembly
cell-substrate junction organization
skin development
regulation of cell-substrate adhesion
response to corticosteroid
regulation of actin filament-based...
cell-matrix adhesion
epidermis development
response to drug
collagen-containing extracellular matrix
focal adhesion
cell-substrate junction
endoplasmic reticulum lumen
cell-cell junction
basement membrane
cell cortex
secretory granule lumen
myofibril
cytoplasmic vesicle lumen
extracellular matrix structural constitu...
cadherin binding
extracellular matrix binding
cell adhesion mediator activity
laminin binding
structural consitituent of cytoskeleton
protein-transporting ATP synthase activit...
pepidase regulator activity
insulin-like growth factor I...
S100 protein binding
protein channel activity
enzyme inhibitor activity
fibronectin binding
insulin-like growth factor binding
integrin binding
GeneNumber
molecular function
cellular component
biological process

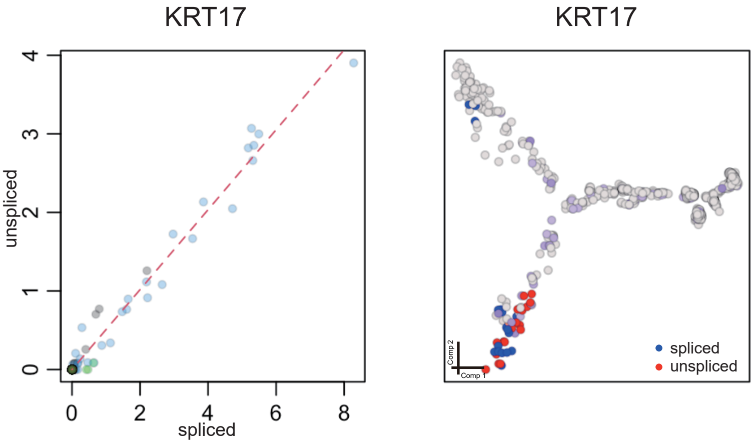
KRT17
unspliced
spliced
0
2
4
6
8
0
1
2
3
4
KRT17
Comp 2
Comp 1
spliced
unspliced

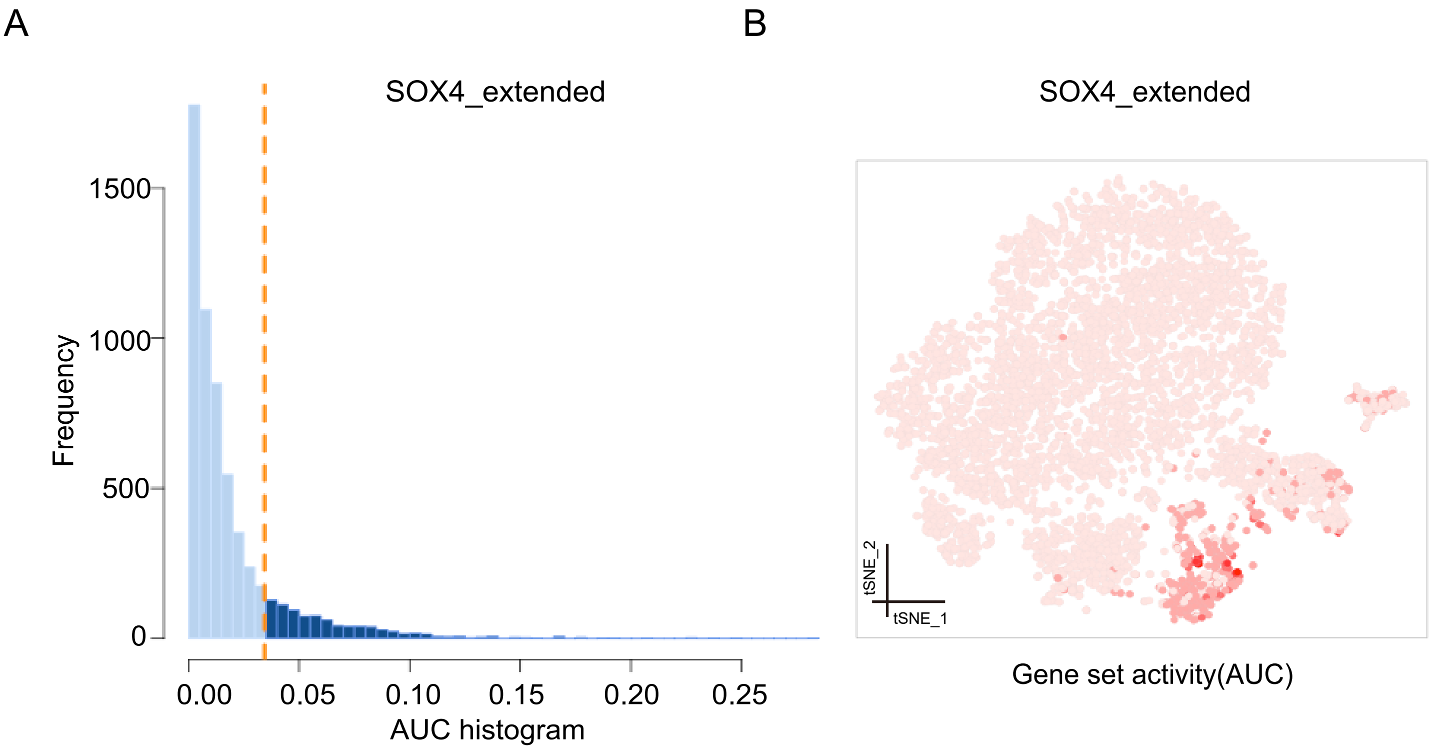

A
SOX4_extended
1500
1000
500
0
Frequency
0.00
0.05
0.10
0.15
0.20
0.25
AUC histogram
B
SOX4_extended
tSNE_2
tSNE_1
Gene set activity(AUC)

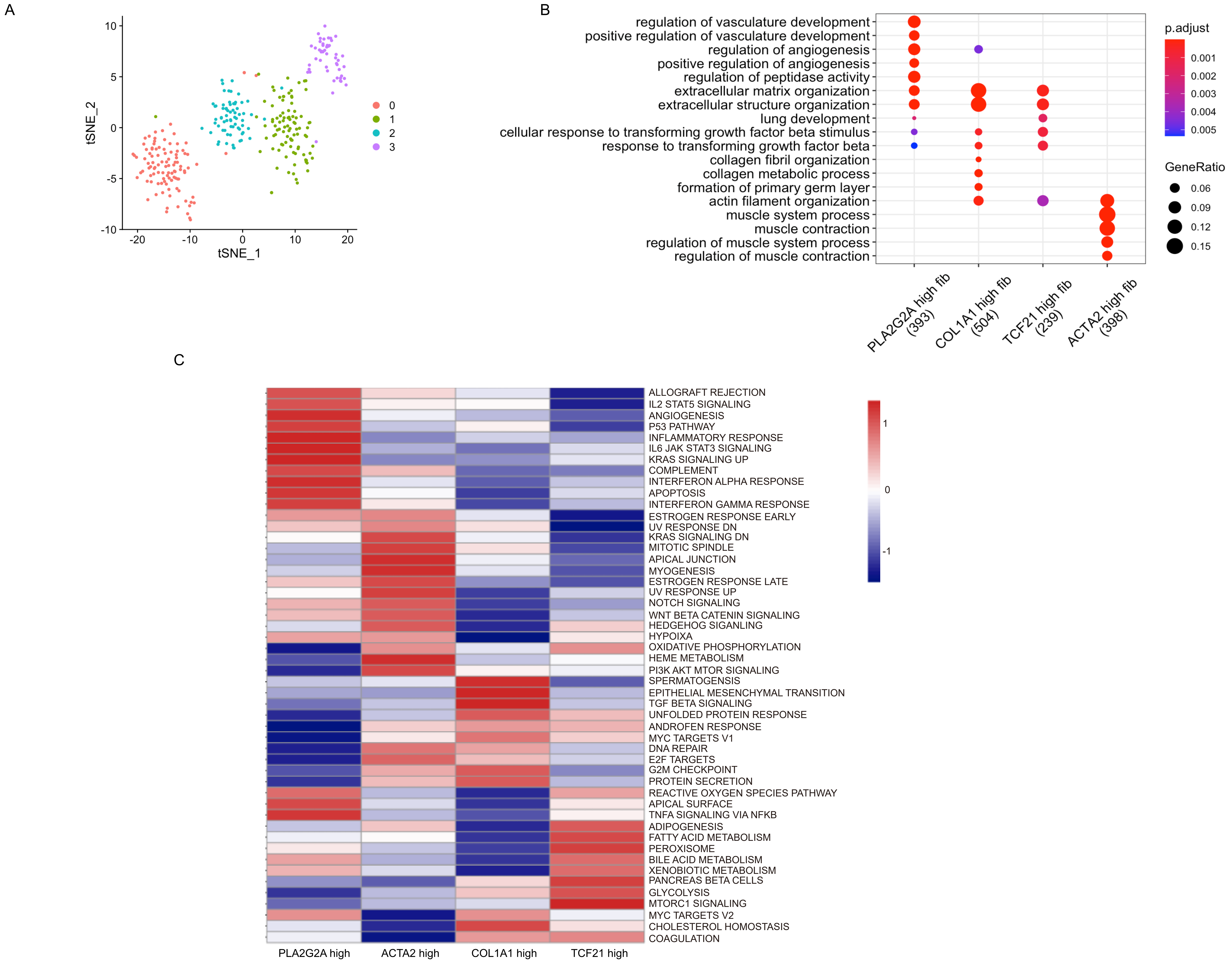

A
tSNE_2
tSNE_1
10
5
0
-5
-10
-20
-10
0
10
20
0
1
2
3
B
regulation of vasculature development
positive regulation of vasculature development
regulation of angiogenesis
positive regulation of angiogenesis
regulation of peptidase activity
extracellular matrix organization
extracellular structure organization
lung development
cellular response to transforming growth factor beta stimulus
response to transforming growth factor beta
collagen fibril organization
collagen metabolic process
formation of primary germ layer
actin filament organization
muscle system process
muscle contraction
regulation of muscle system process
regulation of muscle contraction
PLA2G2A high fib (393)
COL1A1 high fib (504)
TCF21 high fib (239)
ACTA2 high fib (398)
p.adjust
0.001
0.002
0.003
0.004
0.005
GeneRatio
0.06
0.09
0.12
0.15
C
ALLOGRAFT REJECTION
IL2 STAT5 SIGNALING
ANGIOGENESIS
P53 PATHWAY
INFLAMMATORY RESPONSE
IL6 JAK STAT3 SIGNALING
KRAS SIGNALING UP
COMPLEMENT
INTERFERON ALPHA RESPONSE
APOPTOSIS
INTERFERON GAMMA RESPONSE
ESTROGEN RESPONSE EARLY
UV RESPONSE DN
KRAS SIGNALING DN
MITOTIC SPINDLE
APICAL JUNCTION
MYOGENESIS
ESTROGEN RESPONSE LATE
UV RESPONSE UP
NOTCH SIGNALING
WNT BETA CATENIN SIGNALING
HEDGEHOG SIGANLING
HYPOIXA
OXIDATIVE PHOSPHORYLATION
HEME METABOLISM
PI3K AKT MTOR SIGNALING
SPERMATOGENSIS
EPITHELIAL MESENCHYMAL TRANSITION
TGF BETA SIGNALING
UNFOLDED PROTEIN RESPONSE
ANDROFEN RESPONSE
MYC TARGETS V1
DNA REPAIR
E2F TARGETS
G2M CHECKPOINT
PROTEIN SECRETION
REACTIVE OXYGEN SPECIES PATHWAY
APICAL SURFACE
TNFA SIGNALING VIA NFKB
ADIPOGENESIS
FATTY ACID METABOLISM
PEROXISOME
BILE ACID METABOLISM
XENOBIOTIC METABOLISM
PANCREAS BETA CELLS
GLYCOLYSIS
MTORC1 SIGNALING
MYC TARGETS V2
CHOLESTEROL HOMOSTASIS
COAGULATION
PLA2G2A high
ACTA2 high
COL1A1 high
TCF21 high
1
0
-1

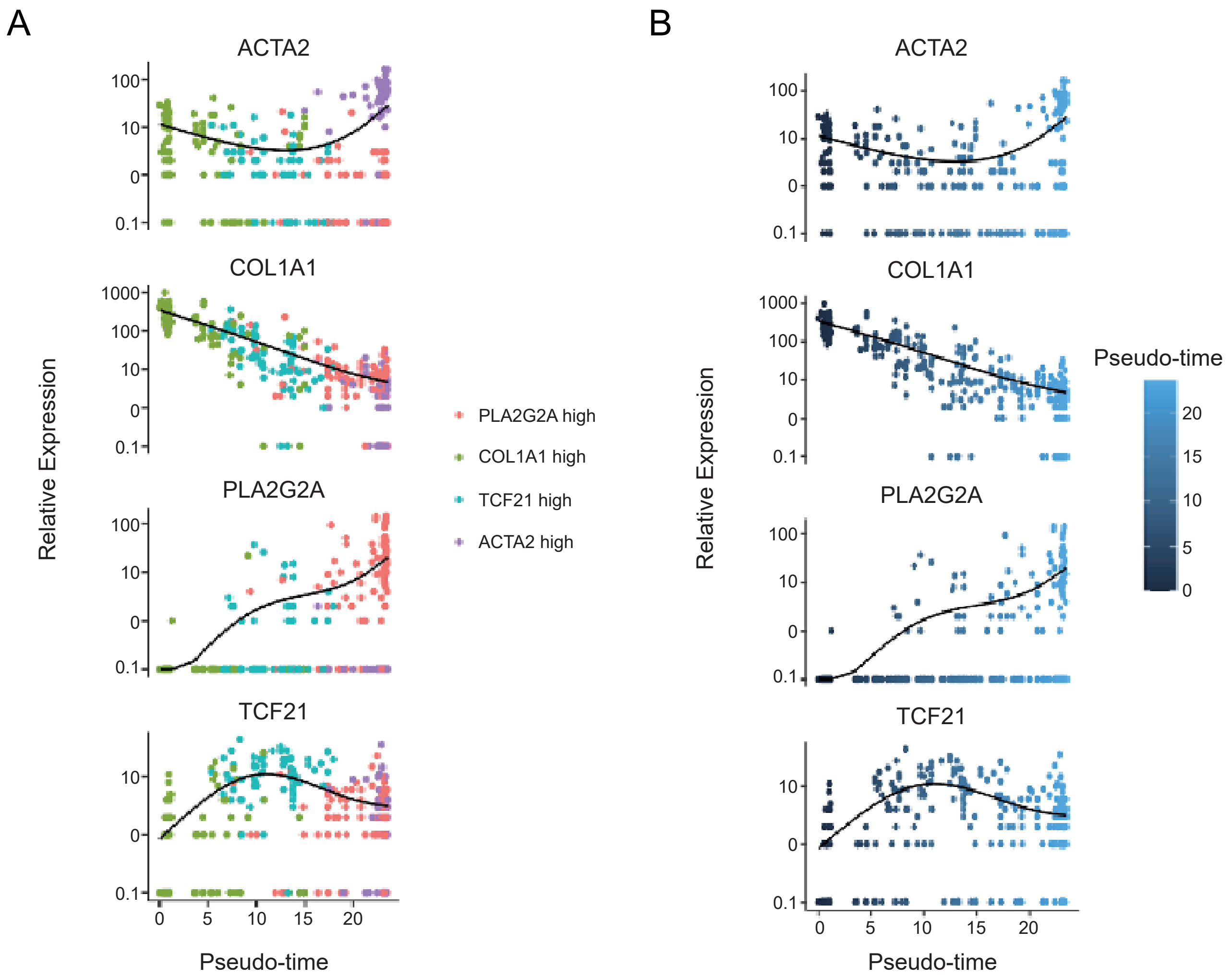
A
ACTA2
COL1A1
PLA2G2A
TCF21
Relative Expression
Pseudo-time
PLA2G2A high
COL1A1 high
TCF21 high
ACTA2 high
B
ACTA2
COL1A1
PLA2G2A
TCF21
Relative Expression
Pseudo-time
Pseudo-time

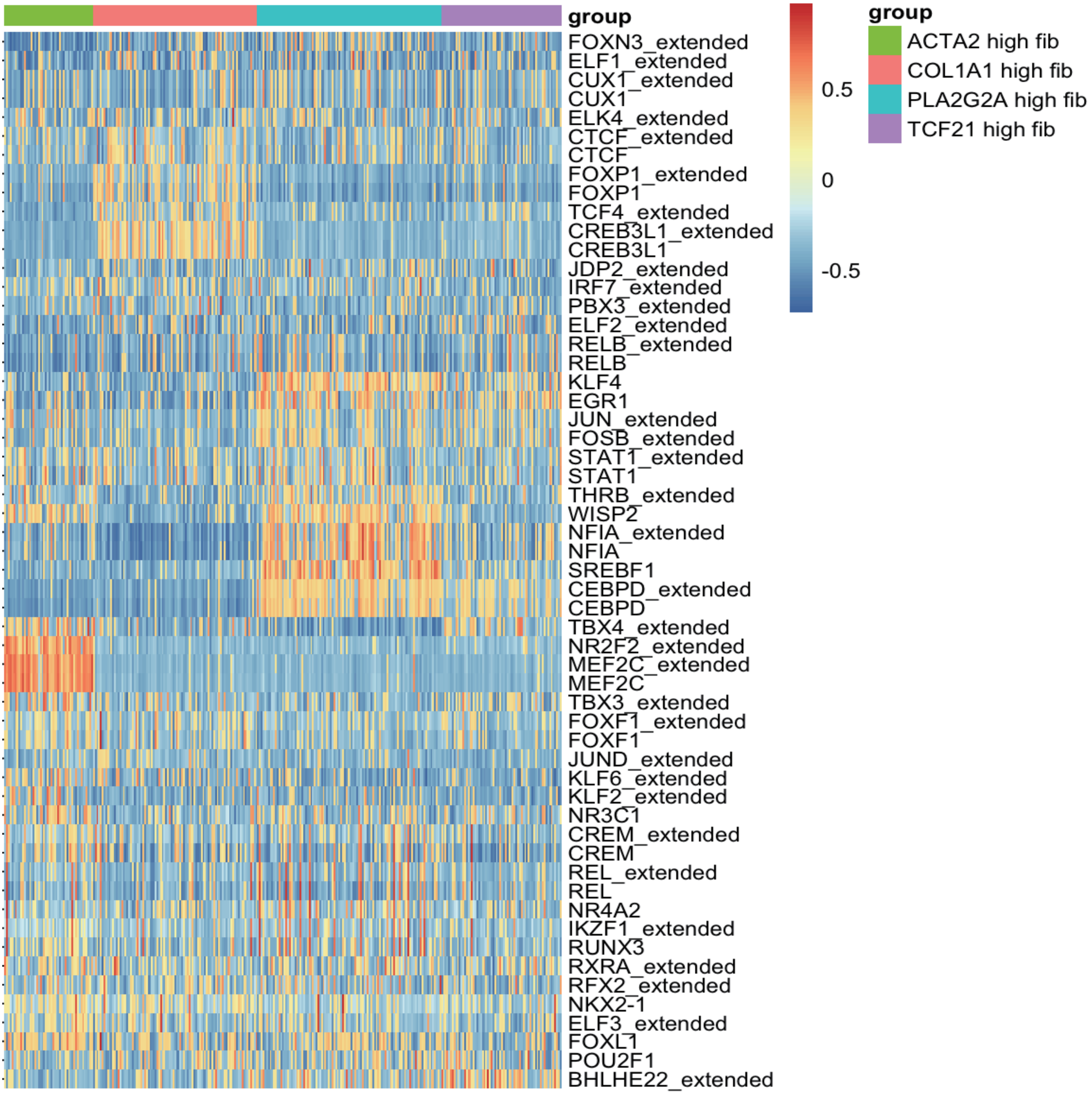
group
FOXN3_extended
ELF1_extended
CUX1_extended
CUX1
ELK4_extended
CTCF_extended
CTCF
FOXP1_extended
FOXP1
TCF4_extended
CREB3L1_extended
CREB3L1
JDP2_extended
IRF7_extended
PBX3_extended
ELF2_extended
RELB_extended
RELB
KLF4
EGR1
JUN_extended
FOSB_extended
STAT1_extended
STAT1
THRB_extended
WISP2
NFIA_extended
NFIA
SREBF1
CEBPD_extended
CEBPD
TBX4_extended
NR2F2_extended
MEF2C_extended
MEF2C
TBX3_extended
FOXF1_extended
FOXF1
JUND_extended
KLF6_extended
KLF2_extended
NR3C1
CREM_extended
CREM
REL_extended
REL
NR4A2
IKZF1_extended
RUNX3
RXRA_extended
RFX2_extended
NKX2-1
ELF3_extended
FOXL1
POU2F1
BHLHE22_extended
0.5
0
-0.5
group
ACTA2 high fib
COL1A1 high fib
PLA2G2A high fib
TCF21 high fib

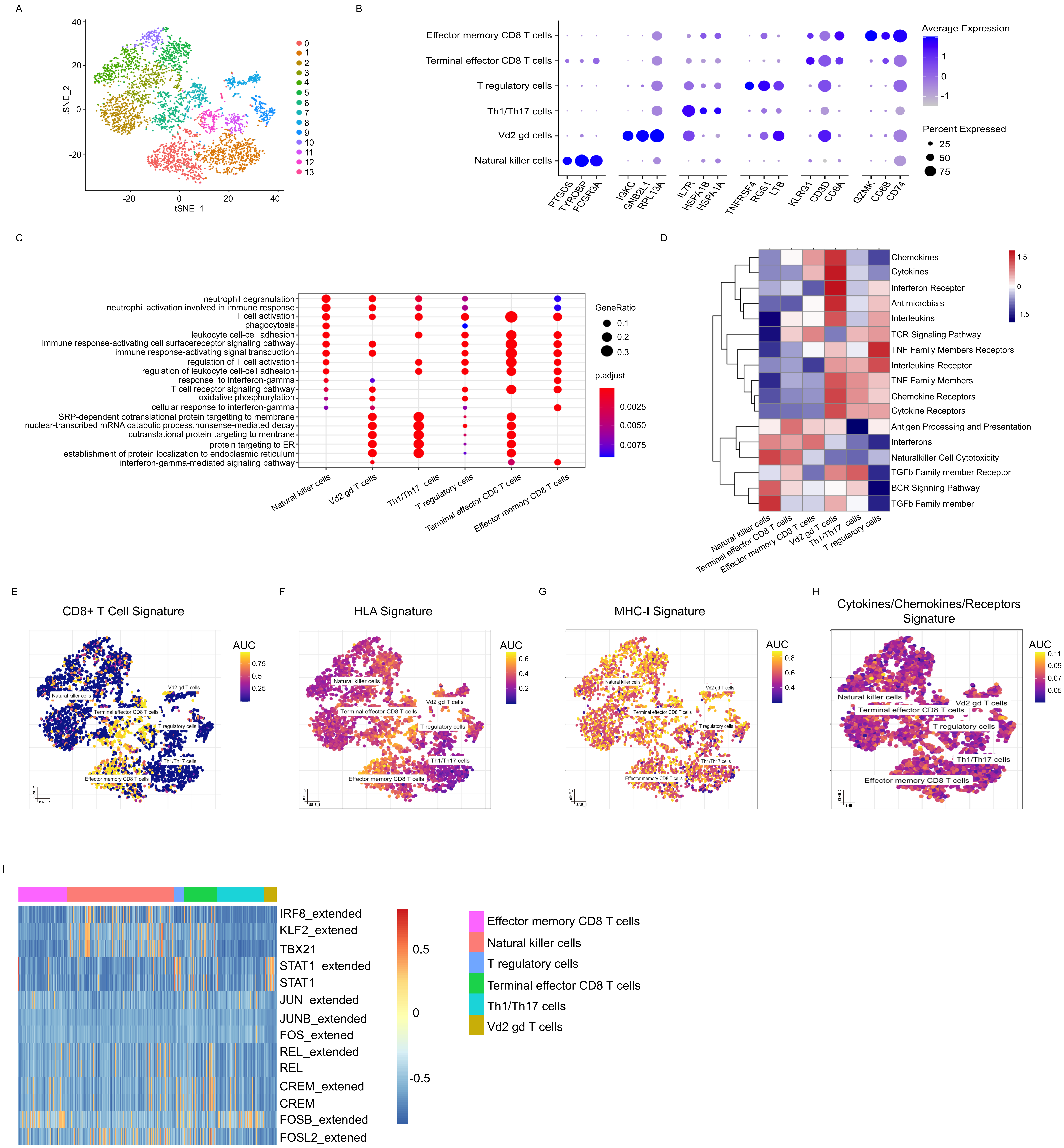
A
B
Effector memory CD8 T cells
Terminal effector CD8 T cells
T regulatory cells
Th1/Th17 cells
Vd2 gd T cells
Natural killer cells
Average Expression
Percent Expressed
PTGDS TYROBP FCGR3A IGKC GNB2L1 RPL13A IL7R HSPA1B HSPA1A TNFRSF4 RGS1 LTB KLRG1 CD3D CD8A GZMK CD8B CD74
C
neutrophil degranulation
neutrophil activation involved in immune response
T cell activation
phagocytosis
leukocyte cell-cell adhesion
immune response-activating cell surface receptor signaling pathway
immune response-activating signal transduction
regulation of T cell activation
regulation of leukocyte cell-cell adhesion
response to interferon-gamma
T cell receptor signaling pathway
oxidative phosphorylation
cellular response to interferon-gamma
SRP-dependent cotranslational protein targetting to membrane
nuclear-transcribed mRNA catabolic process,nonsense-mediated decay
cotranslational protein targeting to mentrane
protein targeting to ER
establishment of protein localization to endoplasmic reticulum
interferon-gamma-mediated signaling pathway
GeneRatio
p.adjust
D
Chemokines
Cytokines
Inferferon Receptor
Antimicrobials
Interleukins
TCR Signaling Pathway
TNF Family Members Receptors
Interleukins Receptor
TNF Family Members
Chemokine Receptors
Cytokine Receptors
Antigen Processing and Presentation
Interferons
Naturalkiller Cell Cytotoxicity
TGFb Family member Receptor
BCR Signning Pathway
TGFb Family member
E
CD8+ T Cell Signature
F
HLA Signature
G
MHC-I Signature
H
Cytokines/Chemokines/Receptors Signature
AUC
I
IRF8_extended
KLF2_extened
TBX21
STAT1_extended
STAT1
JUN_extended
JUNB_extended
FOS_extened
REL_extended
REL
CREM_extened
CREM
FOSB_extended
FOSL2_extened
Effector memory CD8 T cells
Natural killer cells
T regulatory cells
Terminal effector CD8 T cells
Th1/Th17 cells
Vd2 gd T cells

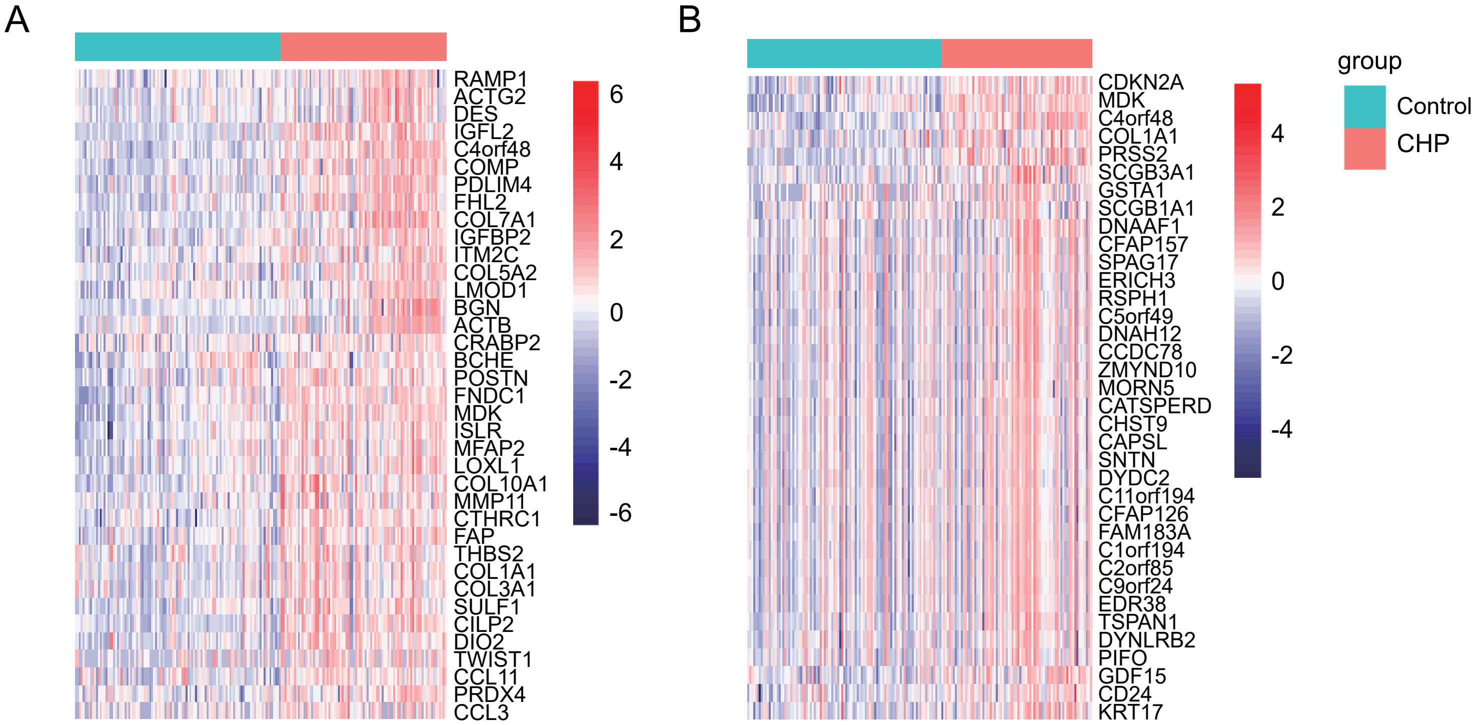
A
RAMP1
ACTG2
DES
IGFL2
C4orf48
COMP
PDLIM4
FHL2
COL7A1
IGFBP2
ITM2C
COL5A2
LMOD1
BGN
ACTB
CRABP2
BCHE
POSTN
FNDC1
MDK
ISLR
MFAP2
LOXL1
COL10A1
MMP11
CTHRC1
FAP
THBS2
COL1A1
COL3A1
SULF1
CILP2
DIO2
TWIST1
CCL11
PRDX4
CCL3
6
4
2
0
-2
-4
-6
B
CDKN2A
MDK
C4orf48
COL1A1
PRSS2
SCGB3A1
GSTA1
SCGB1A1
DNAAF1
CFAP157
SPAG17
ERICH3
RSPH1
C5orf49
DNAH12
CCDC78
ZMYND10
MORN5
CATSPERD
CHST9
CAPSL
SNTN
DYDC2
C11orf194
CFAP126
FAM183A
C1orf194
C2orf85
C9orf24
EDR38
TSPAN1
DYNLRB2
PIFO
GDF15
CD24
KRT17
4
2
0
-2
-4
group
Control
CHP
